# A systems study reveals concurrent activation of AMPK and mTOR by amino acids

**DOI:** 10.1038/ncomms13254

**Published:** 2016-11-21

**Authors:** Piero Dalle Pezze, Stefanie Ruf, Annika G. Sonntag, Miriam Langelaar-Makkinje, Philip Hall, Alexander M. Heberle, Patricia Razquin Navas, Karen van Eunen, Regine C. Tölle, Jennifer J. Schwarz, Heike Wiese, Bettina Warscheid, Jana Deitersen, Björn Stork, Erik Fäßler, Sascha Schäuble, Udo Hahn, Peter Horvatovich, Daryl P. Shanley, Kathrin Thedieck

**Affiliations:** 1 Institute for Cell and Molecular Biosciences (ICaMB), Newcastle University, Newcastle upon Tyne NE2 4HH, UK; 2 Centre for Integrated Systems Biology of Ageing and Nutrition, Newcastle University Institute for Ageing, Newcastle upon Tyne NE4 5PL, UK; 3 Babraham Institute, Babraham Research Campus, Cambridge CB22 3AT, UK; 4 Department of Bioinformatics and Molecular Genetics (Faculty of Biology), Institute for Biology 3, Albert-Ludwigs-University Freiburg, 79104 Freiburg, Germany; 5 Research Training Group (RTG) 1104, Albert-Ludwigs-University Freiburg, 79104 Freiburg, Germany; 6 Department of Pediatrics, University of Groningen, University Medical Center Groningen (UMCG), 9713 AV Groningen, The Netherlands; 7 Department of Neuroscience, School of Medicine and Health Sciences, Carl von Ossietzky University Oldenburg, 26111 Oldenburg, Germany; 8 Spemann Graduate School of Biology and Medicine (SGBM), University of Freiburg, 79104 Freiburg, Germany; 9 Department of Biochemistry and Functional Proteomics, Faculty of Biology, University of Freiburg, 79104 Freiburg, Germany; 10 Institute of Pharmacology and Toxicology, University of Ulm, 89081 Ulm, Germany; 11 BIOSS Centre for Biological Signalling Studies, University of Freiburg, 79104 Freiburg, Germany; 12 Institute of Molecular Medicine I, Medical Faculty, Heinrich-Heine-University, 40225 Düsseldorf, Germany; 13 Jena University Language & Information Engineering (JULIE) Lab, Friedrich-Schiller-University Jena, 07743 Jena, Germany; 14 Faculty of Mathematics and Natural Sciences, Department of Pharmacy, Analytical Biochemistry, University of Groningen, 9713 AV Groningen, The Netherlands

## Abstract

Amino acids (aa) are not only building blocks for proteins, but also signalling molecules, with the mammalian target of rapamycin complex 1 (mTORC1) acting as a key mediator. However, little is known about whether aa, independently of mTORC1, activate other kinases of the mTOR signalling network. To delineate aa-stimulated mTOR network dynamics, we here combine a computational–experimental approach with text mining-enhanced quantitative proteomics. We report that AMP-activated protein kinase (AMPK), phosphatidylinositide 3-kinase (PI3K) and mTOR complex 2 (mTORC2) are acutely activated by aa-readdition in an mTORC1-independent manner. AMPK activation by aa is mediated by Ca^2+^/calmodulin-dependent protein kinase kinase β (CaMKKβ). In response, AMPK impinges on the autophagy regulators Unc-51-like kinase-1 (ULK1) and c-Jun. AMPK is widely recognized as an mTORC1 antagonist that is activated by starvation. We find that aa acutely activate AMPK concurrently with mTOR. We show that AMPK under aa sufficiency acts to sustain autophagy. This may be required to maintain protein homoeostasis and deliver metabolite intermediates for biosynthetic processes.

## Abstract (web summary)

mTORC1 is known to mediate the signalling activity of amino acids. Here, the authors combine modelling with experiments and find that amino acids acutely stimulate mTORC2, IRS/PI3K and AMPK, independently of mTORC1. AMPK activation through CaMKKβ sustains autophagy under non-starvation conditions.

## Graphical abstract

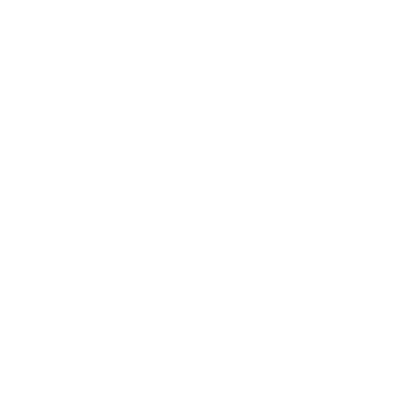

The serine/threonine kinase mammalian target of rapamycin (mTOR) is a central regulator of metabolism and cellular growth, and resides in two multiprotein complexes, mTORC1 and mTORC2 (refs 1, 2, 3) that form part of a wider signalling network (Fig. 1a). mTORC1 contains the specific interactors Raptor (regulatory associated protein of mTOR) and PRAS40 (proline-rich AKT/PKB substrate 40 kDa), whereas mTOR in mTORC2 specifically binds to Rictor (rapamycin-insensitive companion of mTOR) and mSin1 (mammalian stress-activated map kinase-interacting protein 1)^1^. Comparatively little is known about mTORC2, which is activated by insulin in an Akt-independent manner^2^,^4^. In contrast, the signalling cascade via which insulin activates mTORC1 is well described^5^ and involves the IR (insulin receptor) and IRS1 (insulin receptor substrate 1), PI3K (phosphatidylinositide 3-kinase), PDK1 (phosphoinositide-dependent kinase 1), Akt and the heterotrimeric tuberous sclerosis protein complex (TSC1–TSC2), consisting of the proteins TSC1, TSC2 and TBC1D7. TSC1–TSC2 harbours a GTPase-activating protein function towards the small GTPase Rheb (Ras homologue enriched in brain). Akt inhibits TSC1–TSC2, which leads to de-repression of Rheb and activation of mTORC1 at the lysosomes^1^,^3^. The best characterized amino acids (aa) input to the mTOR signalling network is mediated via the Ragulator-Rag GTPase (Ras-related GTPase) complex that recruits mTORC1 to lysosomal membranes, where mTORC1 can be further activated by insulin^5^,^6^. Hence, aa are the primary input to mTORC1 that is necessary for it to be sensitive to insulin.

mTORC1 activates anabolic processes, including protein synthesis and inhibits catabolic processes such as autophagy, which safeguards the cell to maintain protein homoeostasis^1^,^3^,^5^,^7^. mTORC1 exerts these effects by phosphorylating several substrates, including the kinases p70-S6K (ribosomal protein S6 kinase) and ULK1 (Unc-51-like kinase-1). mTORC1 phosphorylates p70-S6K at threonine 389 (p70-S6K-pT389), which enhances protein synthesis^8^. Furthermore, mTORC1 and p70-S6K exert negative-feedback effects on the IR and IRS, rendering the IR-PI3K-Akt signalling axis refractory to insulin^5^. We refer to the sum of these inhibitory mechanisms as ‘negative-feedback loop' (NFL). In addition, positive feedbacks from mTORC1 to IRS have been described^9^,^10^,^11^,^12^,^13^.

mTORC1 phosphorylates ULK1 at serine 757 (ULK1-pS757) to inhibit ULK1-dependent autophagy^14^,^15^. Of note, ULK1 is also phosphorylated by AMPK at S317 (ULK1-pS317), and this event stimulates ULK1-dependent autophagy^14^,^15^. AMPK is typically considered to be activated by nutrient and energy shortage, whereas mTORC1 responds to nutrient readdition and insulin. Therefore, mTORC1 and AMPK are often perceived as antagonists that suppress or induce autophagy under nutrient sufficiency or shortage, respectively^15^. This notion is further strengthened by the fact that under conditions of low-energy (high AMP/ATP ratio) AMPK phosphorylates and activates TSC1–TSC2, thereby inhibiting mTORC1 (refs 5, 15). Yet, it has been more recently noted that autophagy is also required during nutrient readdition, to provide aa and metabolite intermediates for biosynthetic processes^16^. An activating role of AMPK during aa stimulation has so far to the best of our knowledge not been reported, and it remains open whether AMPK sustains autophagy also during nutrient sufficiency.

In this study, we explored if aa signal exclusively via mTORC1, or if other mTOR network components respond independently to aa. As aa activate mTOR in muscle cells, whereas growth factors appear as a secondary input^17^,^18^, we opted for murine C2C12 myocytes as a muscle-related system. We adopted a data-driven systems approach to build hypotheses on potential further aa inputs into the mTOR network in an unbiased manner, based on dynamic time-course data only. For this reason, we started with a computational network structure, in which we incorporated only the well-documented direct aa input to mTORC1 (ref. 6). We built on our earlier modelling work^4^,^19^ to test whether simulations of the dynamic network response to aa could match the measured time-course data, or which additional inputs would improve the fit between them. Strikingly, a network model assuming only a single aa input to the network via mTORC1 did not reproduce our experimental calibration data. In a stepwise strategy, we added further aa inputs *in silico,* and additional network connections via IRS/PI3K, mTORC2 and AMPK markedly improved the model fit with our experimental data. We experimentally confirmed these findings and found that aa acutely activate AMPK via the Ca^2+^/calmodulin-dependent protein kinase kinase β (CaMKKβ). We report that under aa sufficiency, AMPK does not inhibit mTORC1, but sustains ULK1 activity and autophagy.

## Results

### Modelling predicts multiple aa inputs to the mTOR network

**Figure 1:**
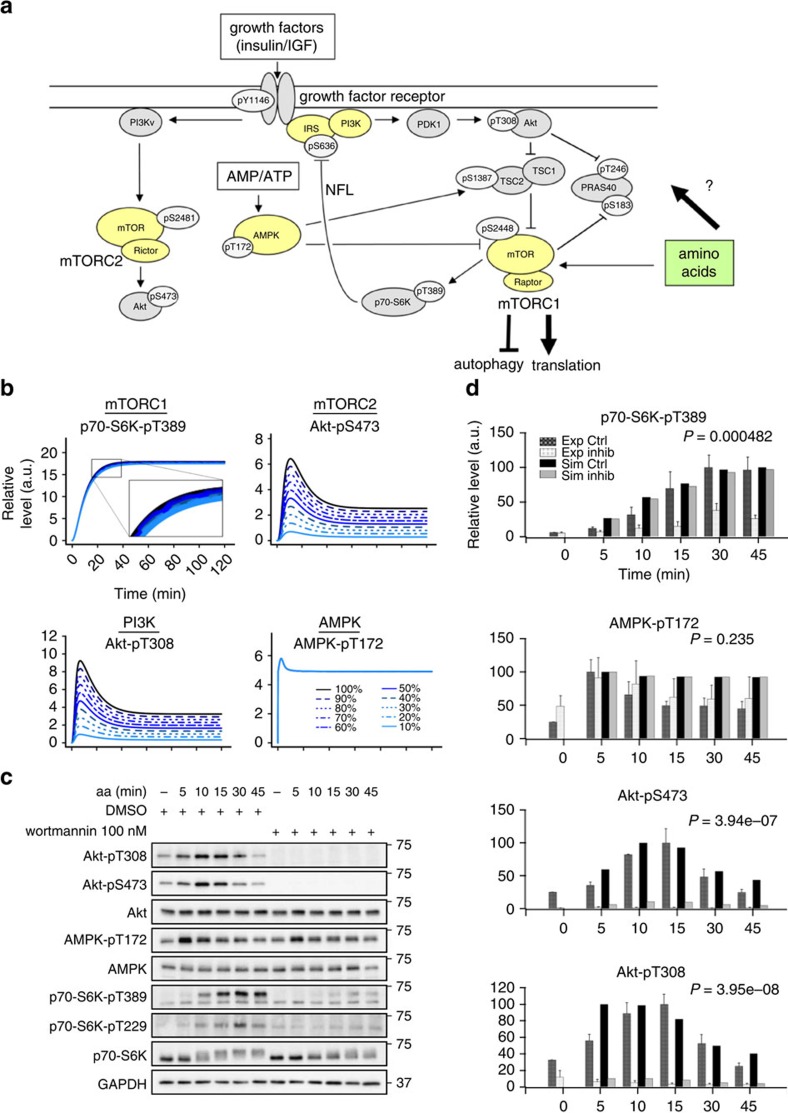
Amino acids activate AMPK independently of PI3K. (**a**) Schematic representation of the mTOR-AMPK network. Amino acids (aa) activate mTORC1. Do they also activate other parts of the network? Nutrients (aa), growth factors and energy (AMP/ATP ratio) impinge on the mTOR (mammalian target of rapamycin) signalling cascade. Growth factors such as insulin activate the IR (insulin receptor). The IR binds IRS1 (insulin receptor substrate 1) which acts as an adaptor for PI3K (phosphoinositide 3-kinase). PI3K generates phosphatidylinositol (3,4,5)-trisphosphate, which binds and translocates PDK1 and Akt/PKB to the plasma membrane. Here, Akt is activated by PDK1 and subsequently inhibits the heteromeric TSC1–TSC2 (tuberous sclerosis protein) complex. TSC1–TSC2 serves as a GTPase-activating protein (GAP) for the small GTPase Rheb (Ras homologue enriched in brain) that activates mTORC1. mTORC1 phosphorylates p70-S6K (p70-S6-kinase), and regulates anabolic processes such as translation and catabolic processes including autophagy. A negative-feedback loop (NFL) from mTORC1 and p70-S6K to IR and IRS1 renders upstream insulin signalling refractory to the insulin input. AMP levels rise when energy demand enhances ATP conversion to ADP. AMP and the kinases LKB1 (liver kinase B1), and CaMKKβ (Ca^2+^/ calmodulin-dependent protein kinase kinase β) activate AMPK (AMP-activated protein kinase), which inhibits mTORC1 by phosphorylating TSC2 and the mTORC1 component Raptor. Next to growth factors and the AMP/ATP ratio, aa activate mTORC1, and this is mediated by the Rag GTPases. (**b**) Simulated response of p70-S6K-pT389, Akt-pS473, Akt-pT308 and AMPK-pT172 to aa stimulation in a system with PI3K perturbation (PI3K activity 10 to 100%; experimental equivalent: PI3K inhibition with wortmannin). (**c**) Aa enhance AMPK-pT172 during PI3K inhibition with wortmannin. Shown are immunoblot results of aa-stimulated C2C12 cells in the presence or absence of 100 nM wortmannin. Data are representative of three experiments. (**d**) Quantitative representations of simulated (PI3K inhibition: residual activity 10% as shown in **b**) and experimentally determined dynamics of p70-S6K-pT389, AMPK-pT172, Akt-pS473 and Akt-pT308 upon stimulation with aa with or without wortmannin. Shown are the mean and s.e.m., *N*=3. Statistical significance between control and treatment in experimental quantitations across the time course was detected by repeated measures analysis of variance (ANOVA). Exp Ctrl, experimental control condition (DMSO); Exp Inhib, experimental PI3K inhibition (wortmannin); Sim Ctrl, simulated control condition; Sim Inhib, simulated PI3K perturbation.

In the present study, we used computational modelling as a means of hypothesis building to steer experiments addressing the regulation of the mTOR network by aa. Our previous model^19^, which we used as a starting point, simulated mTOR network dynamics in response to combined insulin and aa stimulation (model structure in Supplementary Fig. 1). To integrate aa as a discrete input, we calibrated our dynamic model with time-course data from C2C12 myocytes that were stimulated with insulin plus aa (Supplementary Fig. 2a), or with aa only (Supplementary Fig. 2b). Cells were lysed at different time points post stimulation from 1 to 120 min, and readouts across the mTOR network (Fig. 1a) were detected and quantified across at least three experiments as described^4^,^19^. The detected readouts and the core model structure covered the following signalling connections (Fig. 1a; additional detail in Supplementary Fig. 1): the insulin signal is transduced via IR autophosphorylation at Y1146 (refs 20, 21), IRS, PI3K and PDK1 that phosphorylates Akt at T308 (ref. 22). Akt activation, monitored by phosphorylation of its substrate PRAS40 at T246, inhibits the TSC1–TSC2 complex by phosphorylation at T1462 (ref. 22); and TSC1–TSC2 inhibits mTORC1 activity^22^, as monitored by the phosphorylation of its substrates PRAS40-S183 and p70-S6K-T389, and the p70-S6K substrate mTOR-S2448 (ref. 22). Akt-pS473 is the *bona fide* mTORC2 activity readout^22^. Autophosphorylation on mTOR-S2481 reflects either mTORC1 or mTORC2 activity, depending on the complex stoichiometries in different cell types^4^,^22^,^23^. p70-S6K inhibits IRS both by direct phosphorylation at S636/639 and by suppression of IRS gene expression^24^,^25^,^26^. In addition, we have recently shown that AMPK is connected to mTORC1 via another IRS-dependent NFL^19^. Activation of AMPK, monitored by phosphorylation of AMPK-T172, inhibits mTORC1 by activating TSC2 via phosphorylation at S1387 and multiple phosphorylation of Raptor itself^15^. We introduced the following enhancements: we expanded the detail of Akt and PRAS40 to include all possible combinations of key phosphorylation states. The TSC1–TSC2 species could be phosphorylated by Akt-pT308 or by AMPK. The former leads to the inactivation of TSC1–TSC2 and activation of mTORC1, whereas the latter promotes TSC1–TSC2-dependent inhibition on mTORC1. IRS and PI3K were considered separately to enable simulation of treatment with the PI3K inhibitor wortmannin. We included two PI3K species, PI3K_PDK1 that binds IRS and activates Akt, and PI3K_variant that we have shown to activate mTORC2 in an Akt-independent manner^4^ (Supplementary Fig. 1).

We modelled the aa input as directly connected to the network via mTORC1, which is activated by the Rag GTPases^5^. The profile of the dynamics elicited by combined insulin and aa input was reflected by simulation with the model based on the established network structure (Supplementary Fig. 3a). However, simulations based on this network structure could not match the experimental data for aa stimulation alone (Supplementary Fig. 3b) which was particularly evident for AMPK (AMPK-pT172, TSC2-pS1387), IRS/PI3K (Akt-pT308), Akt (PRAS40-pT246) and mTORC2 (Akt-pS473). The quality of the fit between this model and our data reported a value of ∼1,415 using the Akaike information criterion (AIC)^27^ (Table 1, single aa input). We speculated that other network components might respond to aa independently of mTORC1. Thus, we calculated the AIC after introducing a second aa input via each network component in addition to mTORC1. This led to the generation of 12 models, each characterized by a specific second aa input (Table 1, double aa input). We considered that a model with *n* aa inputs represented a significant improvement with respect to the best model having *n*−1 aa inputs, when the AIC of the former was at least 1% less than the AIC of the latter (black rows in Table 1). This threshold of 1% was chosen to avoid including solutions that could arise from numerical approximation in parameter estimation. When testing models with two aa inputs, we found that the model assuming aa inputs on mTORC1 and IRS reported an AIC of ∼953, improving the model fit as compared to the model with one single aa input. The AIC for the models with an additional aa input at TSC1–TSC2-pT1462, p70-S6K-pT389 or PRAS40-pS183 did not improve by at least 1% (AIC>1,401, red text in Table 1), and these models were consequently discarded. We next investigated whether a third aa-responsive component could further improve the fit. This was achieved by testing all the 36 models (Table 1, triple aa input) created by adding third aa inputs to each model with AIC improvement from the previous group. We found that the model assuming aa inputs on mTORC1, IRS-p and mTORC2 showed an AIC of ∼798, improving the fit quality with respect to the best-computed model having two aa inputs. At this level, most of the model combinations did not present an improvement (AIC>943, red) with respect to the best model of the previous group (double aa input to mTORC1 and IRS1-p), and only seven models obtained an improvement in terms of AIC. We repeated the procedure considering quadruple aa input models (Table 1, quadruple aa input) selected from combinations of these remaining models. We found that only two models reported an AIC improvement with respect to the best model of the previous group (AIC≤790). These two models included aa inputs on IRS-p, AMPK-pT172 and mTORC2 (AIC∼680), or IRS-p, AMPK-pT172 and PI3K_Variant (AIC∼723), in addition to the aa input on mTORC1. We focused on the first of these two models for the following three reasons: (a) no data on the species PI3K_Variant is currently available, (b) the PI3K_Variant was modelled as upstream of mTORC2 only and therefore its aa-dependent activation also propagated the signal to mTORC2 and (c) the first model returned a better fit quality. The complete definition of the best-fit model (AIC∼680) is provided in Supplementary Tables 1–4. The list of the replaced reactions generating all the intermediate models is shown in Supplementary Table 3. Details for parameter estimation and identifiability are reported in Supplementary Table 4, Supplementary Figs 4–12, and further described in the methods section.

In summary, the model-based analysis of our data yielded hypotheses on further aa inputs to the mTOR network via IRS/PI3K, AMPK and mTORC2 that are not mediated by mTORC1 (Fig. 1a). When we compared the model simulations for the network structure with the quadruple aa input to our experimental data, we found that the simulations qualitatively reproduced the response dynamics of the mTOR network to insulin plus aa (Supplementary Fig. 13a), as well as to aa alone (Supplementary Fig. 13b) for all readouts across the network. We next investigated the sensitivity of the network to each of the inputs, beginning with the inputs not mediated by mTORC1.

### PDK1 is critical to render p70-S6K PI3K sensitive

To analyse the contribution of PI3K to mTOR network activation by aa, we inhibited PI3K by wortmannin. We generated time-course data following aa stimulation by model simulations (Fig. 1b) and experiments (Fig. 1c). The simulation predicted aa responsiveness of the PI3K/PDK1 readout Akt-pT308 and the mTORC2 readout Akt-pS473, which were strongly suppressed by wortmannin. This is because the two PI3K variants, which can both be inhibited by wortmannin, are upstream of PDK1, Akt and mTORC2 (ref. 4). In contrast, the AMPK response to aa was predicted to remain largely unaffected by PI3K inhibition (Fig. 1b). These predictions were confirmed by our experimental data. Whereas Akt-pT308, and Akt-pS473 inductions by aa were inhibited by wortmannin, AMPK remained responsive to aa (Fig. 1c,d). Thus, the AMPK response to aa is not mediated by PI3K.

**Figure 2:**
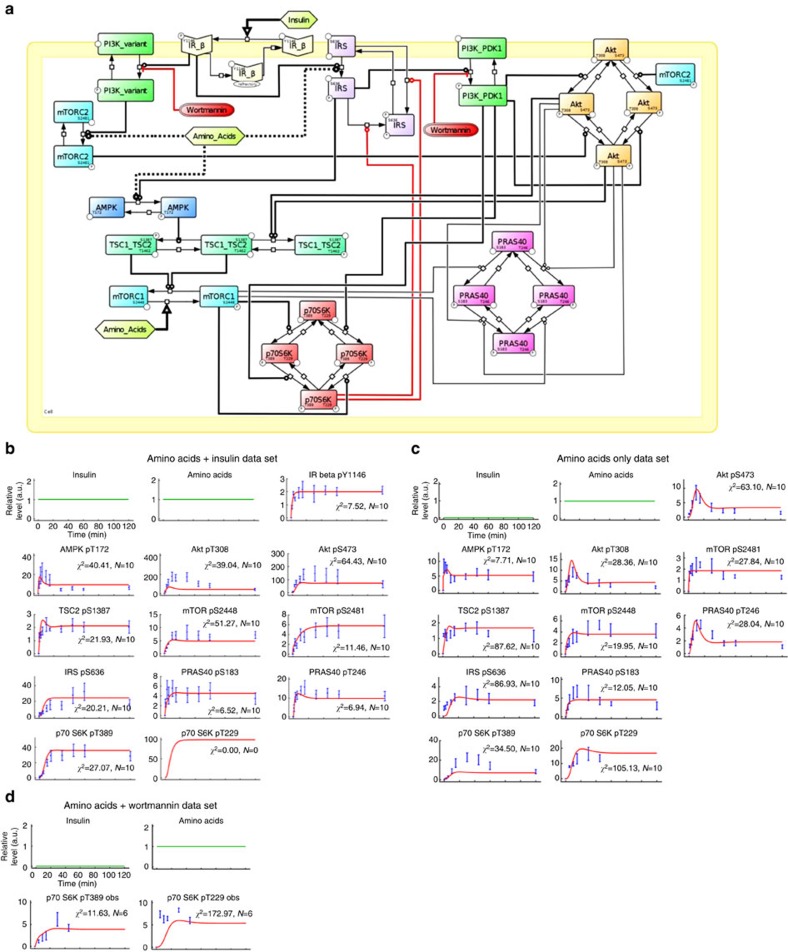
mTOR network model with four amino acid inputs and an extended p70-S6K module fits the experimental data. (**a**) Graphical model of the mTOR network activated by insulin and four amino acid inputs (Table 1). (**b**) Comparison between the simulated time courses (solid lines) and the experimental time courses (points and dotted error bars represent mean and s.e.m.) within 0 to 120 min of aa and insulin stimulation. The values on the *y* axes are relative intensities that are specific for each readout and cannot be compared among readouts. (**c**) As in **b**, in the absence of insulin. Stimulation with aa only. (**d**) As in **b**, in the absence of insulin, in the presence of wortmannin.

**Figure 3:**
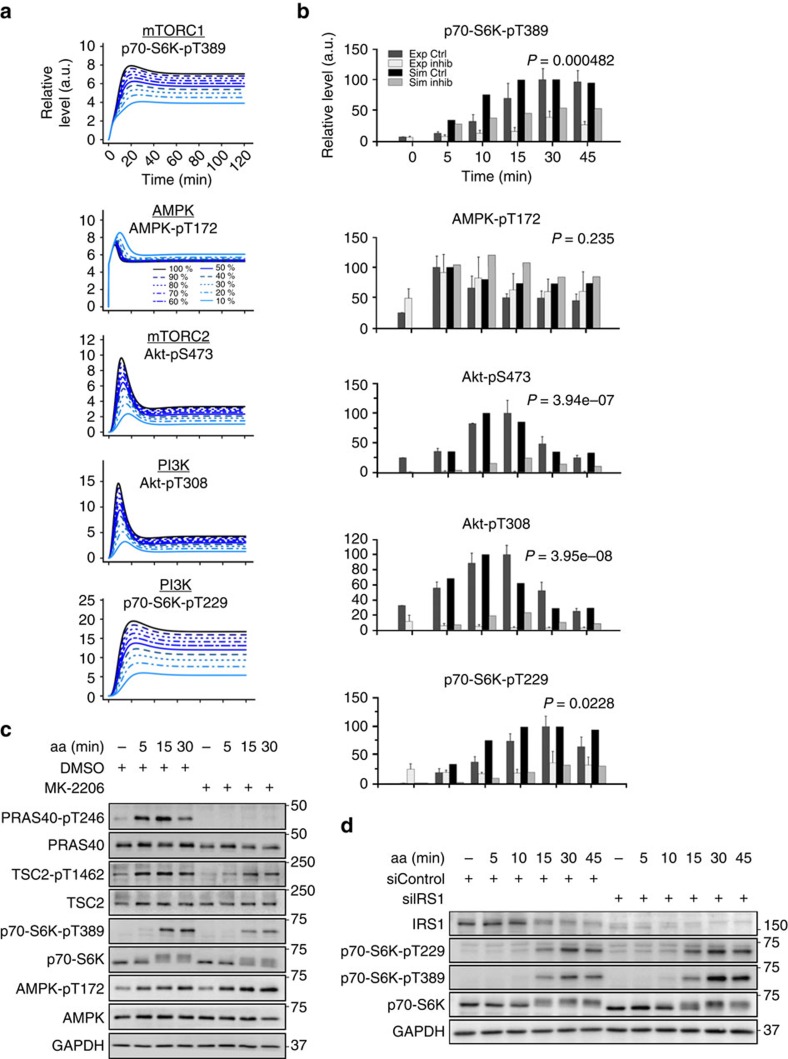
Simulations and experiments show that amino acids (aa) activate mTORC1 via the PI3K-Akt signalling axis in an IRS1-independent manner. (**a**) Simulated time courses corresponding to the perturbation as shown in Fig. 1b, but using the model including the new p70-S6K extended module. The simulated time courses for p70-S6K-pT389 and pT229 also incorporate the corresponding experimental data that was used for parameter estimation (Fig. 1d). The simulated time courses for Akt and AMPK represent predictions using this new model. (**b**) Equivalent quantitative representations as shown in Fig. 1d, but using the model including the new p70-S6K extended module. Simulated quantifications are based on data from **a** of this figure. Experimental data are the same as shown in Fig. 1d and reported here to compare it with the new model. Statistical significance between control and treatment in experimental quantitations across the time course was detected by repeated measures analysis of variance (ANOVA). Exp Ctrl, experimental control condition (DMSO); Exp Inhib, experimental PI3K inhibition (wortmannin); Sim Ctrl, simulated control condition; Sim Inhib, simulated PI3K perturbation. Shown are the mean and s.e.m., *N*=3. (**c**) The allosteric Akt inhibitor MK-2206 reduces mTORC1 activation by aa. Aa-enhanced p70-S6K-pT389 is reduced upon Akt inhibition with MK-2206, whereas AMPK-T172 remains phosphorylated. Shown are immunoblot results of aa-stimulated C2C12 cells in the presence or absence of 1 μM MK-2206. Data are representative of three experiments. (**d**) IRS1 knockdown does not inhibit mTOR signalling upon aa stimulation. Aa readdition enhances p70-S6K-pT389 and pT229 in control C2C12 myocytes transfected with non-targeting siRNA (siControl) or with IRS1 knockdown (siIRS1). Shown are immunoblot results of aa-stimulated C2C12 cells with and without IRS1 knockdown. Data are representative of three experiments.

The simulations correctly reproduced the behaviour of Akt and AMPK in response to wortmannin, but we noted that the experimental inhibition of p70-S6K-pT389 by wortmannin was much more prominent in the experimental data (Fig. 1c) than predicted by the model (Fig. 1b). A comparison of data obtained from simulations, and experiments for control and inhibition with wortmannin at different time points are provided in two complementary formats: as bar graphs in Fig. 1d and as scatter and line plots in Supplementary Fig. 14. Bar graphs help draw attention to the contrast between control and inhibition between simulation and experiment, whereas the scatter and line format highlights model fit to data. In either representation, it is clear that the model predictions for inhibition with wortmannin for p70-S6K-pT389 are not reproduced in the data, highlighting a poor sensitivity of the p70-S6K module to PI3K inhibition. Why did our model correctly reproduce the wortmannin response of Akt, but not of p70-S6K? Akt and p70-S6K are both AGC kinases that need to be phosphorylated twice for full activation: by PDK1 within the so-called T-loop, and by a phosphoinositide-dependent protein kinase 2 (PDK2) within the hydrophobic motif^28^. Although the PDK1 is identical for all AGC kinases, the identity of the PDK2 differs between AGC kinases. In the case of Akt-S473, mTORC2 is considered the major PDK2, and mTORC1 acts as the PDK2 on p70-S6K-T389: these connections were both present in our computational model. However, whereas Akt-pT308 was directly connected to PI3K, to account for the action of PDK1, there was no such connection for the PDK1 target site p70-S6K-T229 (ref. 28). We reasoned that the sensitivity of p70-S6K to PI3K in our model may be improved with inclusion of this mechanism. Thus, we added a connection between p70-S6K-T229 and PI3K-PDK1 to our model (Fig. 2a), and we recorded p70-S6K-T229 phosphorylation kinetics (Fig. 1c, quantitation in Fig. 2c,d). For model calibration, the p70-S6K-pT229 data in response to aa with and without perturbation by wortmannin were used, in addition to the full set of kinetic data in response to aa+insulin, or aa only (details on parameter estimation and identifiability analysis provided in Supplementary Tables 5–7, Supplementary Fig. 15 and methods). The comparison between simulations and data for all readouts across the network revealed a good fit for both the calibration data sets (Fig. 2b,c) and for the kinetic data upon aa stimulation that was part of the wortmannin perturbation data set (Fig. 2d). Comparison of Supplementary Fig. 13b with Fig. 2c shows that inclusion of the direct PI3K/PDK1-p70-S6K link into the model decreased the fit for some readouts, such as Akt-pT308, Akt-pS473 and AMPK-pT172 in response to aa+insulin. Yet, we judged it more important that the fit was much improved for p70-S6K-pT389 (Fig. 2d), as compared with a model without the PDK1-p70-S6K link (Supplementary Fig. 13c). The simulation of p70-S6K-pT389 now showed sensitivity to a simulated PI3K inhibition (Fig. 3a), within the same range as in our wortmannin experiments (Figs 3b and 1c; Supplementary Fig. 16). The effects of aa stimulation and wortmannin in terms of increase or decrease of readouts, including Akt-pT308, Akt-pS473 and AMPK-pT172, remained consistent between simulations and measured data across the time course. The model predicted a gradual reduction for p70-S6K-pT229 upon gradual PI3K inhibition (Fig. 3a), and this inhibitory effect was confirmed experimentally (Figs 3b and 1c; Supplementary Fig. 16). The AMPK-pT172 simulation showed a slight increase upon PI3K inhibition, possibly as a result of a more pronounced mTORC1-dependent NFL inhibition. Although the experimental data recapitulated this tendency, the effect was too small to be statistically significant (Fig. 3b; Supplementary Fig. 16). The conclusion remained that the AMPK response to aa is not mediated by PI3K, whereas PI3K mediates aa signals to Akt, mTORC1 and p70-S6K.

Wortmannin is a broad spectrum PI3K inhibitor that blocks not only class I PI3Ks, upstream of Akt, but also the class III PI3K vps34. We used here a wortmannin concentration of 100 nM, at and below which this inhibitor is specific for class I PI3Ks (ref. 29); but off-target effects cannot be excluded. Vps34 has been suggested to mediate a direct stimulatory aa signal to mTORC1 (ref. 30), independently of class I PI3Ks and Akt. To validate if aa signalling to mTORC1 requires Akt, we used the allosteric Akt inhibitor MK-2206 (ref. 31) in combination with aa stimulation for 5, 15 and 30 min (Fig. 3c). MK-2206 inhibited Akt, as monitored by the reduction of the Akt readouts PRAS40-pT246 and TSC2-pT1462. MK-2206 also inhibited phosphorylation of the mTORC1 substrate p70-S6K-T389, whereas aa-enhanced AMPK phosphorylation at T172 remained unaffected. This suggests that Akt, downstream of class I PI3Ks, indeed mediates an aa signal to mTORC1, but not to AMPK.

IRS1, upstream of PI3K, not only receives negative-feedback signals from mTORC1, but also positive-feedback regulation of IRS1 by mTORC1 has been reported^9^,^10^,^11^,^12^,^13^. Hence, mTORC1 could activate class I PI3K via IRS1 in response to aa. To test this possibility, we targeted IRS1 by short interfering RNA (siRNA) in combination with aa stimulation for 5 up to 45 min (Fig. 3d). The knockdown was efficient as monitored by the reduced IRS1 levels. However, aa-enhanced phosphorylation of the PDK1 substrate site p70-S6K-pT229, downstream of PI3K, remained unaltered by the IRS1 knockdown. This suggests that PI3K activation by aa does not require IRS1, and that positive feedback from mTORC1 to IRS1 does not mediate aa-enhanced class I PI3K signalling in C2C12 cells. Furthermore, phosphorylation of the mTORC1 substrate p70-S6K-T389 was not reduced in cells without IRS1, suggesting that IRS1 does not mediate mTORC1 activation by aa. In summary, we conclude that aa signal to mTORC1 via class I PI3K and Akt, whereas AMPK activation by aa is PI3K and Akt independent.

### Amino acids activate AMPK independently of mTORC2

**Figure 4:**
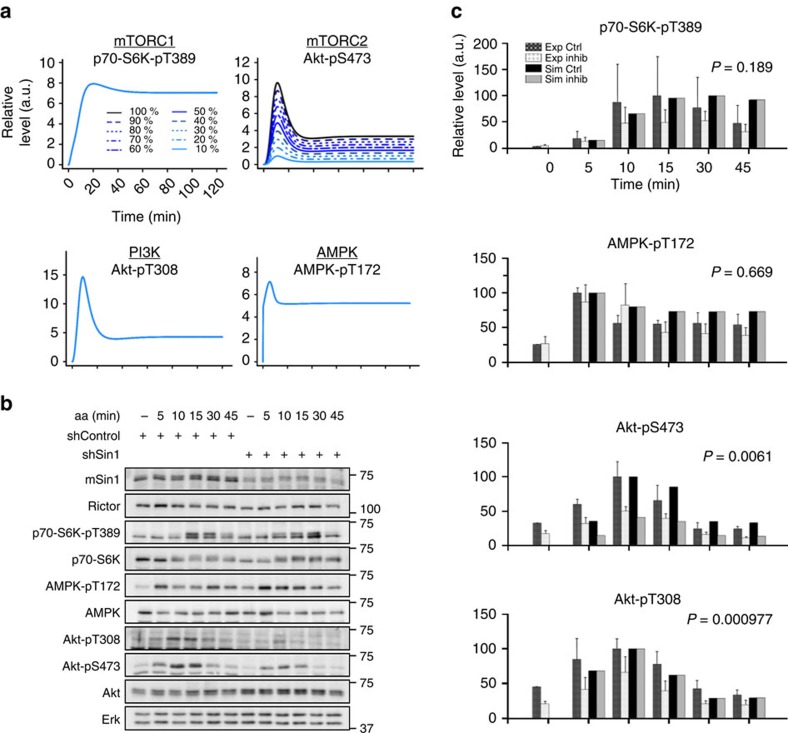
Amino acids (aa) activate mTORC1 and AMPK independently of mTORC2. (**a**) Simulated response of p70-S6K-pT389, Akt-pS473, Akt-pT308 and AMPK-pT172 to aa induction in a system with mTORC2 inhibition (mTORC2 activity 10 to 100%; experimental equivalent: mSin1 knockdown). (**b**) Aa induce p70-S6K-pT389 and AMPK-pT172, when mTORC2 is inhibited by mSin1 knockdown (shSin1). Representative immunoblot results of aa-stimulated C2C12 cells in the absence or presence of shSin1 (4 days). Data are representative of three experiments. (**c**) Quantitative representations of simulated (mTORC2 inhibition: reduction to 40%, as shown in **a**) and experimentally determined dynamics of p70-S6K-pT389, AMPK-pT172, Akt-pS473 and Akt-pT308 upon stimulation with aa with mTORC2 inhibition using shSin1. Data are the mean and s.e.m., *N*=3. Statistical significance between control and treatment in experimental quantitations across the time course was detected by repeated measures analysis of variance (ANOVA). Exp Ctrl, experimental control condition (shControl); Exp Inhib, experimental mTORC2 inhibition (shSin1); Sim Ctrl, simulated control condition; Sim Inhib, simulated mTORC2 perturbation.

We next analysed if the observed responses of Akt-pS473, Akt-pT308 and AMPK-pT172 to aa were positively connected to each other, for example, via feedback mechanisms, or if they represent separate aa inputs to the mTOR network. We inhibited mTORC2 *in silico* (Fig. 4a) and experimentally (Fig. 4b), using an inducible mSin1 knockdown (shSin1) C2C12 cell line and recorded time-course data upon aa stimulation. The model simulation predicted that if the four inputs (mTORC1, mTORC2, PI3K and AMPK) were unrelated, mTORC2 inhibition (reflected by a reduction of Akt-pS473) should not affect aa stimulation of p70-S6K-pT389, AMPK-pT172 or Akt-pT308 (Fig. 4a). Indeed, we observed that upon mTORC2 inhibition by shSin1, Akt-pS473 was reduced, whereas p70-S6K-pT389 and AMPK-pT172 remained responsive to aa (Fig. 4b,c; Supplementary Fig. 17). In contrast to our simulation, Akt-pT308 was reduced by mTORC2 inhibition. This may be due to the fact that this readout not only reflects PI3K activity, but that Akt-T308 phosphorylation efficiency depends also in part on the phosphorylation at S473 (refs 32, 33). For completeness, a comparison between the prediction of the model without p70-S6K-pT229 module and experimental data upon mTORC2 inhibition is provided in Supplementary Fig. 18, showing consistent results.

In summary, the AMPK and mTORC1 responses to aa are not mediated by mTORC2, whereas we cannot experimentally exclude an mTORC2 interdependence with the PI3K/PDK1/Akt axis due to the interdependence of Akt-pS473 (mediated by mTORC2) and Akt-pT308 (mediated by PDK1). As both phosphorylation sites depend on PI3K-generated phosphatidylinositol (3,4,5)-trisphosphate binding to Akt, they do not ultimately allow to distinguish between mTORC2 and PI3K activity^4^. Of note, aa have been previously suggested to activate class I PI3K and mTORC2^34^; however, this study also used Akt-pT308 and Akt-pS473 as readouts and therefore does not allow a conclusion on the interdependence of PI3K and mTORC2 activation by aa.

**Figure 5:**
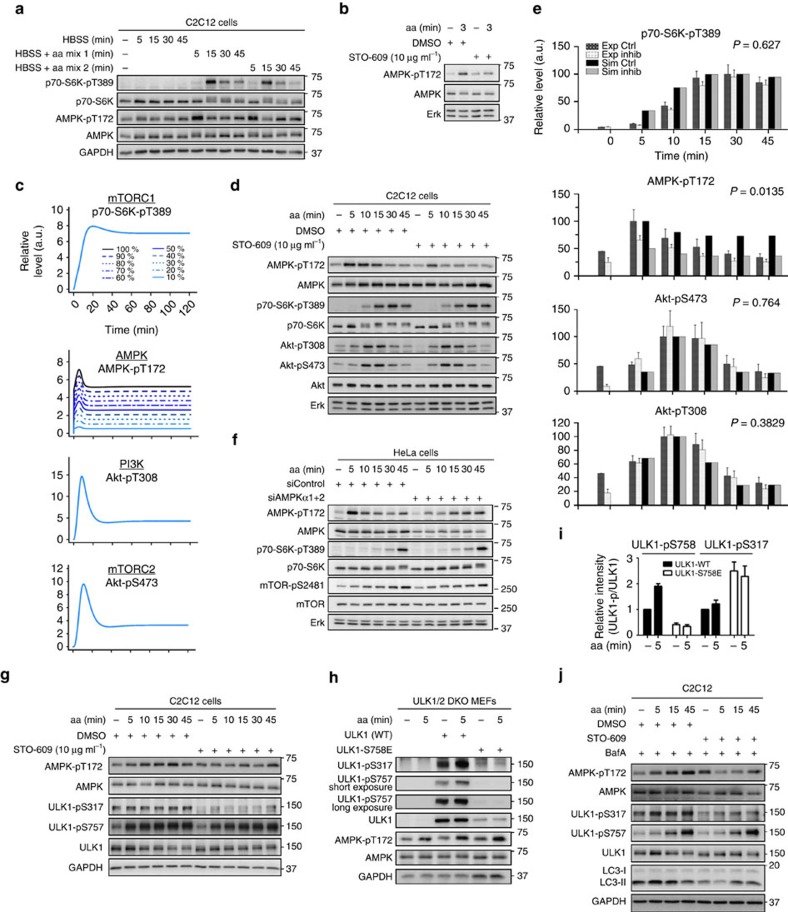
Amino acids (aa) activate mTORC1, mTORC2 and PI3K independently of AMPK, and phosphorylation of the AMPK substrate ULK1 is CaMKKβ dependent. (**a**) Different aa mixes enhance AMPK-T172 and p70-S6K-T389 phosphorylation. C2C12 cells were starved in HBSS (16 h), with subsequent readdition of HBSS+aa mix 1, HBSS+aa mix 2 or HBSS only. *N*=3. aa mix 1: Arg, Asn, Asp, Cys, Glu, Gln, Gly, His, OH-Pro, Ile, Leu, Lys, Met, Phe, Pro, Ser, Thr, Trp, Tyr, Val; aa mix 2: Arg, Cys, Glu, His, Ile, Leu, Lys, Met, Phe, Thr, Trp, Tyr, Val. (**b**) AMPK-pT172 induction by aa is inhibited by the CaMKKβ inhibitor STO-609. Aa-stimulated C2C12 cells were analysed with or without 10 μg ml^−1^ STO-609. *N*=3. (**c**) Simulated response of p70-S6K-pT389, Akt-pS473, Akt-pT308 and AMPK-pT172 to aa stimulation and AMPK perturbation (AMPK activity 10 to 100%; experimental equivalent: STO-609). (**d**) Aa readdition enhances p70-S6K-pT389, Akt-pT308 and Akt-pS473 in the presence of STO-609. Aa-stimulated C2C12 cells were analysed with or without 10 μg ml^−1^ STO-609. *N*=4. (**e**) Quantitative representations of simulated (AMPK inhibition 50%, corresponds to **c**) and measured dynamics of p70-S6K-pT389, AMPK-pT172, Akt-pS473 and Akt-pT308 upon aa, with or without STO-609. Shown are the mean and s.e.m., *N*=4. Statistical significance between control and treatment in experimental quantitations across the time course was detected by repeated measures analysis of variance (ANOVA). Exp Ctrl, experimental control condition (DMSO); Exp Inhib, experimental AMPK perturbation (STO-609); Sim Ctrl, simulated control condition; Sim Inhib, simulated AMPK perturbation. (**f**) Aa readdition enhances p70-S6K-pT389, Akt-pT308 and mTOR-pS2481 in cells with AMPK knockdown (siAMPKα1+2). Aa-stimulated HeLa cells were analysed with or without siAMPKα1+2 (2 days). *N*=3. (**g**) Aa enhance ULK1-pS757 (mTORC1 substrate site), and ULK1-pS317 (AMPK substrate site) remains phosphorylated. STO-609 reduces ULK1-pS317 but not ULK1-pS757. Aa-stimulated C2C12 cells were analysed with or without 10 μg ml^−1^ STO-609. *N*=3. (**h**) ULK1 can be phosphorylated at the AMPK substrate site serine 317, when the mTORC1 substrate site (ULK1-S758 in human, corresponding to S757 in mouse) is mutated to glutamate (ULK1-S758E). ULK1/2 double-knockout (DKO) mouse embryonic fibroblasts (MEFs) were left untransfected, or stably transfected with human wild-type ULK1 (ULK1-WT), or ULK1-S758E. *N*=6. (**i**) Relative quantitation of data shown in **h**. Signals for ULK1-pS317 and ULK1-pS758 in ULK1-WT and ULK1-S758E were quantified and normalized to ULK1 total levels. Values for ULK1-WT, starvation condition were set to 1. Mean values and s.e.m., *N*=6. (**j**) STO-609 reduces LC3-II autophagy marker levels in aa-stimulated cells. Aa-stimulated C2C12 cells were analysed with or without 10 μg ml^−1^ STO-609. All cells were pre-treated with Bafilomycin A_1_ (BafA) for 60 min. *N*=3.

The finding that AMPK activation by aa is not mediated by the PI3K/PDK1/Akt axis, or mTORC2 was intriguing as it suggested that AMPK is activated by aa, and to the best of our knowledge this activation is not covered in the existing literature. We validated this finding by stimulating C2C12 cells with two other combinations of aa in Hank‘s Balanced Salt Solution (HBSS) medium (Fig. 5a), and observed that these aa mixes also enhanced AMPK-T172 phosphorylation at 5 min post aa readdition, whereas S6K-pT389 was enhanced later at 15 min post aa readdition. This matched our previous observations (Figs 1c, 2c, 3c and 4b; Supplementary Fig. 2b), and we therefore went on to investigate AMPK regulation by aa in further detail.

### AMPK activation by aa requires CaMKKβ

Our simulations and experiments showed that AMPK acutely responds to aa as early as 1 min post aa readdition (Supplementary Fig. 2b). Which molecular component may mediate such a rapid response? Although so far not connected to AMPK activation by aa, CaMKKβ had been reported as an AMPK activator in response to increased cytoplasmic Ca^2+^ levels^15^. Therefore, we tested if the CaMKKβ inhibitor STO-609 suppressed AMPK activation by aa, and indeed STO-609 inhibited aa-stimulated AMPK-T172 phosphorylation (Fig. 5b).

To analyse the possible effect of aa-activated AMPK on the mTOR network, we inhibited AMPK *in silico* (Fig. 5c) and experimentally by STO-609 (Fig. 5d). AMPK-T172 phosphorylation was suppressed by STO-609 at early time points post-aa readdition. In contrast, mTORC2 (Akt-pS473), PI3K (Akt-pT308) and mTORC1 (p70-S6K-pT389) remained aa responsive when AMPK was inhibited (Fig. 5d). We quantified the measured readouts and compared them to the simulated species, which supported our conclusion that inhibition of AMPK does not affect mTORC1, mTORC2 or PI3K activation by aa (Fig. 5e; Supplementary Fig. 19). For completeness, a comparison between the prediction of the model without p70-S6K-pT229 module, and experimental data upon AMPK inhibition is provided (Supplementary Fig. 20), showing consistent results.

As this finding is central to the present study, we aimed to confirm it by further experimental set-ups. In C2C12 cells, we were technically unable to achieve reliable AMPK inhibition by knockdown or expression of a dominant negative AMPK version. We therefore switched to another cell type and performed AMPK knockdown in HeLa cells in combination with an aa time-course analysis (Fig. 5f, quantitation and statistics in Supplementary Fig. 21). In line with our results in C2C12 myocytes, we found that AMPK-T172 phosphorylation was acutely enhanced by aa readdition. Interestingly, HeLa cells lack LKB1 (liver kinase B1), which next to CaMKKβ is the other *bona fide* AMPK inducing kinase^15^. Thus, LKB1 is dispensable for AMPK activation by aa. AMPK knockdown in HeLa cells did not affect the readouts of mTORC2 (mTOR-pS2481) or mTORC1 (p70-S6K-pT389). Thus, the aa input to AMPK is independent from the ones to mTORC1 and mTORC2. Furthermore, the fact that p70-S6K-pT389 was not increased by AMPK knockdown (Fig. 5f) or CaMKKβ inhibition (Fig. 5d) suggests that AMPK—upon aa stimulation—is not a negative regulator of mTORC1, which is in contrast to other physiological conditions, such as energy or nutrient stress^15^.

### Amino acid-activated AMPK keeps ULK1 and autophagy active

As mTORC1 remains active when AMPK is activated by aa, we asked which other AMPK substrates may respond to aa stimulation. AMPK activates the autophagy-driving kinase ULK1 by phosphorylation at S317 (ref. 14). Thus, we tested if this phosphorylation can be detected in aa-stimulated C2C12 cells (Fig. 5g). Indeed, ULK1-S317 phosphorylation was detected both in starved and aa-stimulated cells, and this phosphorylation was inhibited by the CaMKKβ inhibitor STO-609. This suggests that upon aa readdition, AMPK sustains ULK1 activity in a CaMKKβ-dependent manner. mTORC1 inhibits ULK1 by phosphorylating ULK1-S757. We found that aa enhanced ULK1-S757 phosphorylation, and this was insensitive to CaMKKβ inhibition, again validating that mTORC1 activation by aa is insensitive to the CaMKKβ-AMPK signalling axis. As ULK1 phosphorylation by AMPK or mTORC1 has been suggested to be mutually exclusive in glucose-starved cells^14^, we tested in aa-stimulated cells if ULK1 could be still phosphorylated at the AMPK substrate site (S317), when the mTORC1 substrate site (S757 in mouse, corresponding to S758 in human) was phosphorylated (Fig. 5h). For this purpose, we stably transfected ULK1/2 double-knockout (DKO) mouse embryonic fibroblasts (MEFs) with either human wild-type ULK1 (ULK1-WT), or an ULK1 variant in which the mTORC1 substrate site S758 (corresponding to mouse ULK1-S757) was mutated to glutamate, to mimic constitutive phosphorylation at this site (ULK1-S758E). Aa stimulation enhanced ULK1-WT phosphorylation at the mTORC1 substrate site S758, as expected (Fig. 5i). For ULK1-S758E-expressing cells, the anti-ULK1-pS758 antibody did not detect a signal, due to the mutagenesis. Expression levels of ULK1-S758E were much lower than for ULK1-WT, leading to a lower ULK1-pS317 signal for the ULK1-S758E variant. However, when the S317 phosphorylation signal was normalized to the ULK1 total levels, no reduction in ULK1-S317 phosphorylation was observed in ULK1-S758E as compared to ULK1-WT (Fig. 5i). This suggests that in aa-stimulated cells, ULK1 can be phosphorylated by mTORC1 and AMPK at the same time. ULK1 phosphorylates and inhibits AMPK^35^ and we observed that ULK1-S758E expression enhanced AMPK-T172 phosphorylation, as compared with ULK1-WT expression (Fig. 5h). This suggests that ULK1-S758E is less active and that the glutamate mimics an inhibitory phosphorylation. In the present study, ULK1 was not part of our computational model, but it will be intriguing to address negative feedback from ULK1 to AMPK under aa sufficiency in the future.

As ULK1 is inhibited by mTORC1 but activated by AMPK, leading to inhibition or activation of autophagy, respectively^14^, we next asked if AMPK drives autophagy in aa-stimulated cells. For this purpose, we detected the autophagy marker microtubule-associated protein 1 light chain 3 (LC3)^36^ in aa-stimulated C2C12 cells, with or without CamKKβ-AMPK inhibition (Fig. 5j). Unprocessed LC3 (LC3-I) resides in the cytoplasm. During autophagy, LC3-I is conjugated with phoshatidylethanolamine, and referred to as LC3-II. In addition, LC3-II is degraded by lysosomal proteases upon autophagosomal–lysosomal fusion, decreasing the LC3-II signal. To prevent LC3-II degradation in the autophagy assay, we added the v-ATPase inhibitor Bafilomycin A_1_ (BafA) to all media. BafA blocks autophagosomal–lysosomal fusion and LC3-II degradation. Thus, LC3-II accumulation can be reliably detected. In agreement, BafA treatment enhanced LC3-II in aa-stimulated C2C12 cells (Supplementary Fig. 22). We stimulated C2C12 cells with aa for 5–45 min, with and without inhibiting the CaMKKβ-AMPK signalling axis by STO-609. As observed previously (Fig. 5g), STO-609 inhibited AMPK-T172 and ULK1-S317 phosphorylation, but not aa-enhanced phosphorylation of the mTORC1 substrate site ULK1-S757 (Fig. 5j). Of note, STO-609 treatment significantly reduced the autophagy marker LC3-II under starvation and 5 min aa readdition (Fig. 5j, quantitation and statistics in Supplementary Fig. 23), suggesting that under aa sufficiency the CaMKKβ-AMPK signalling axis keeps an ULK1 fraction activated and sustains basal autophagy.

### The CaMKKβ-AMPK axis inhibits c-Jun in aa-stimulated cells

**Figure 6:**
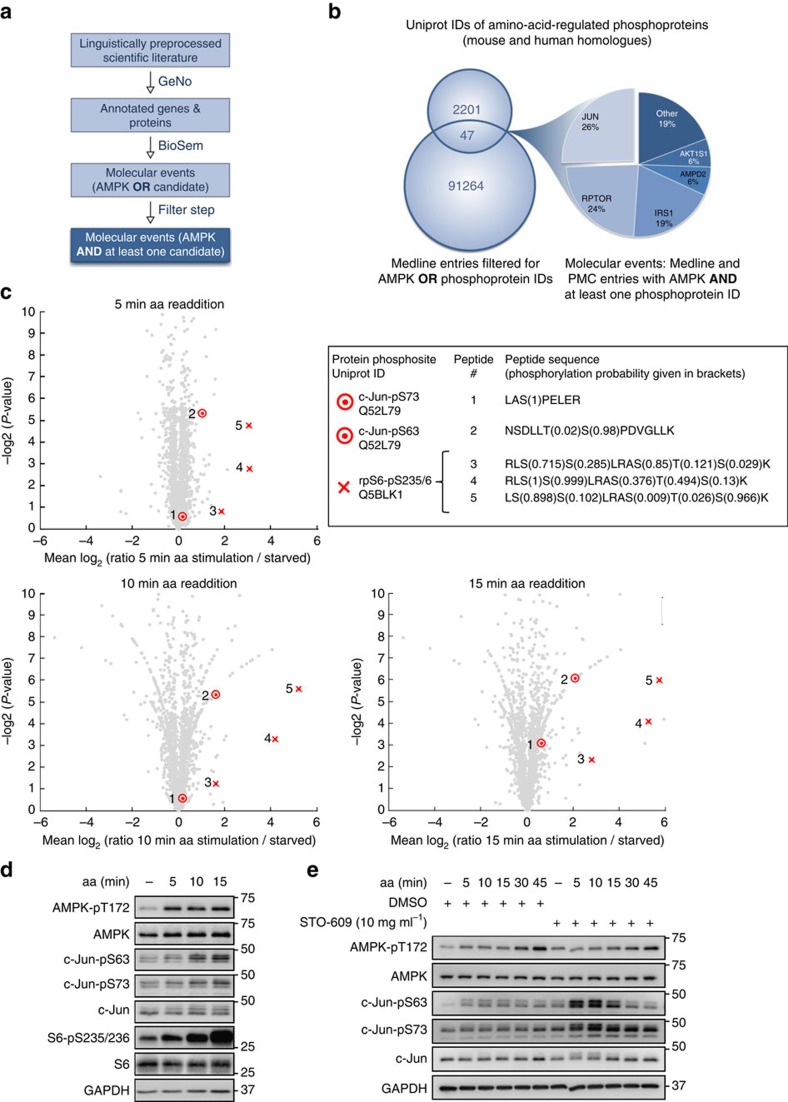
A phosphoproteomics text mining pipeline identifies c-Jun as an amino acid (aa) target that is inhibited by the CaMKKβ-AMPK axis. (**a**) Schematic workflow of text mining pipeline. The tools GeNo^84^ and BioSem^86^ were used to identify gene and protein text occurrences and associated molecular events described in available scientific literature (Medline and PubMed Central, PMC). The resulting event list was filtered for joint occurrences of AMPK and phosphoproteins listed in Supplementary Data 1 and 2 (twofold regulated, see Methods for details). (**b**) Text mining results. The extrapolated candidate list contained 2,201 UniProt IDs (Supplementary Data 3). Linguistic preprocessing and filtering of the Medline and PMC open access literature resulted in 91,264 documents containing either AMPK or one of the phosphoprotein candidates. Molecular event detection by BioSem was broadened to the full-text range, whenever available through PMC open access. Subsequent filtering for AMPK and one of the candidates occurring as event partners yielded 47 molecular events. (**c**) Volcano plot using SILAC ratio of all phosphosites (grey dots) on aa induction at time points 5, 10 and 15 min versus 0 min. Highlighted in red are phosphopeptides containing the phosphosites S6-pS235/236, c-Jun-pS63 and c-Jun-pS73. Primary aa sequence and phosphorylation site localization probabilities (in brackets) of the highlighted phosphopeptides are listed in the table. (**d**) Aa stimulation enhances S6-pS235/236, c-Jun-pS63 and c-Jun-pS73. Shown are immunoblot results of aa-stimulated C2C12 cells. Data are representative of three experiments. (**e**) Aa further enhance c-Jun-pS63 and c-Jun-pS73 when CaMKKβ is inhibited by STO-609. Aa-stimulated C2C12 cells were analysed in the presence or absence of 10 μg ml^−1^ STO-609. Data are representative of three experiments.

To comprehensively analyse aa-stimulated phosphorylation events that depend on AMPK, we adopted a quantitative phosphoproteomics approach in combination with text mining-enhanced data analysis. As AMPK responds acutely to aa, we analysed the phosphoproteome at 5, 10 and 15 min upon aa readdition. 2,517 phosphopeptides containing 2,078 confidently localized phosphosites were identified. Applying a ratio cutoff of 1.5 and a *P*-value cutoff of 0.05 (see Methods and Supplementary Data 1 and 2 for details), 296 phosphopeptides were significantly regulated at at least one time point (Supplementary Data 1 for phosphosites, and Supplementary Data 2 for phosphopeptides). With a twofold cutoff, 108 phosphopeptides remained significantly regulated. We continued the analysis with this more stringent data set. Consistent with a major role of mTOR in aa signalling, the gene ontology (GO) terms mTOR- and mTORC1-mediated signalling were significantly enriched (Supplementary Fig. 24b). To systematically evaluate for each candidate in our proteomics data set whether it could be regulated by AMPK, we adopted an automated Medline and PubMed Central (PMC) wide-text analysis. The text mining pipeline included automated recognition of genes and proteins in abstracts in Medline, and the open access subset of PMC full texts (Fig. 6a). We filtered from the complete text corpus for all abstracts that referenced either AMPK or at least one of the candidates from our proteomics data set. As this data set contained *Mus musculus* UniProt IDs only, the candidate list was extended with the homologous *Homo sapiens* associated UniProt IDs, leading to a total of 2,201 UniProt IDs (Supplementary Data 3). Linguistic preprocessing and filtering of the Medline and PMC open access literature yielded 91,264 documents containing at least one of the candidate UniProt IDs (Fig. 6b). We then employed a molecular event recognizer and identified events (for example, regulation relationship between two genes) in abstracts. Molecular event detection by the BioSem tool was broadened to the full-text range, whenever available from the PMC open access corpus. Finally, we filtered for events that mention candidates from our proteomics data set together with AMPK (Methods), yielding 47 molecular events. Next to the mTORC1 upstream regulator IRS1 and the mTORC1 components Raptor (RPTOR) and PRAS40 (AKT1S1), whose crosstalk with AMPK is known^3^,^19^, c-Jun/Jun appeared in 26% of the resulting molecular events (Fig. 6b; Supplementary Data 3). As c-Jun had to the best of our knowledge not been previously linked with nutrient stimulation, we decided to further analyse the response of c-Jun to aa readdition. Two known activating c-Jun phosphorylation sites were identified with high confidence in our phosphoproteomic data. Fragment spectra of the two c-Jun peptides containing either phosphosite are shown in Supplementary Fig. 24c. We plotted the ratios for all phosphosites at 5, 10 and 15 min aa readdition versus starved cells as volcano plots, and marked the positions of the two identified c-Jun phosphosites (Fig. 6c). As a positive control, we analysed phosphosites of the known mTORC1 downstream target ribosomal protein S6 (S6)^3^. S6 is the substrate of p70-S6K that is upregulated by aa^37^. As expected, we found that S6 phosphorylation was increased by aa at all time points. (Fig. 6c, peptides #3–5). c-Jun phosphorylation at serine 63 (Fig. 6c, peptide #2) was upregulated at all three time points, and further increased at 10 and 15 min of aa readdition. c-Jun phosphorylation at serine 73 (Fig. 6c, peptide #1) was also slightly increased ∼1.54-fold at 15 min aa readdition even though it stayed below the significance threshold. We validated this observation by immunoblotting and found that both c-Jun-pS63 and c-Jun-pS73 were upregulated by aa stimulation (Fig. 6d). In agreement with the mass spectrometry (MS) data (Fig. 6c, peptides #1,2), c-Jun phosphorylation was also in immunoblots more pronounced at S63 than at S73, and phosphorylation of the positive control S6 at serine 235/236 was increased (Fig. 6d). Thus, aa readdition enhances phosphorylation of c-Jun. To test whether this event is regulated by the CaMKKβ-AMPK axis, we combined aa stimulation with CaMKKβ inhibition by STO-609 and analysed c-Jun-pS63 and pS73 (Fig. 6e). Of note, STO-609 increased c-Jun phosphorylation, suggesting that CaMKKβ-AMPK activation by aa inhibits c-Jun activity. This could be mediated by a phosphatase or a kinase that is activated or inhibited by AMPK, respectively. Interestingly, c-Jun has been reported to suppress autophagy^38^, suggesting that c-Jun inhibition by CaMKKβ-AMPK could sustain autophagy under nutrient sufficiency.

### Akt and AMPK remain aa responsive when mTORC1 is inhibited

The best-described mediator of mTORC1 activation by aa are the Rag GTPases, and this mechanism is considered independent of PI3K, AMPK or mTORC2^5^. Nevertheless, regulatory connections between mTORC1 and AMPK are known (Fig. 1a). Therefore, we tested if the newly discovered stimulatory input of aa on AMPK was mTORC1 dependent or not.

**Figure 7:**
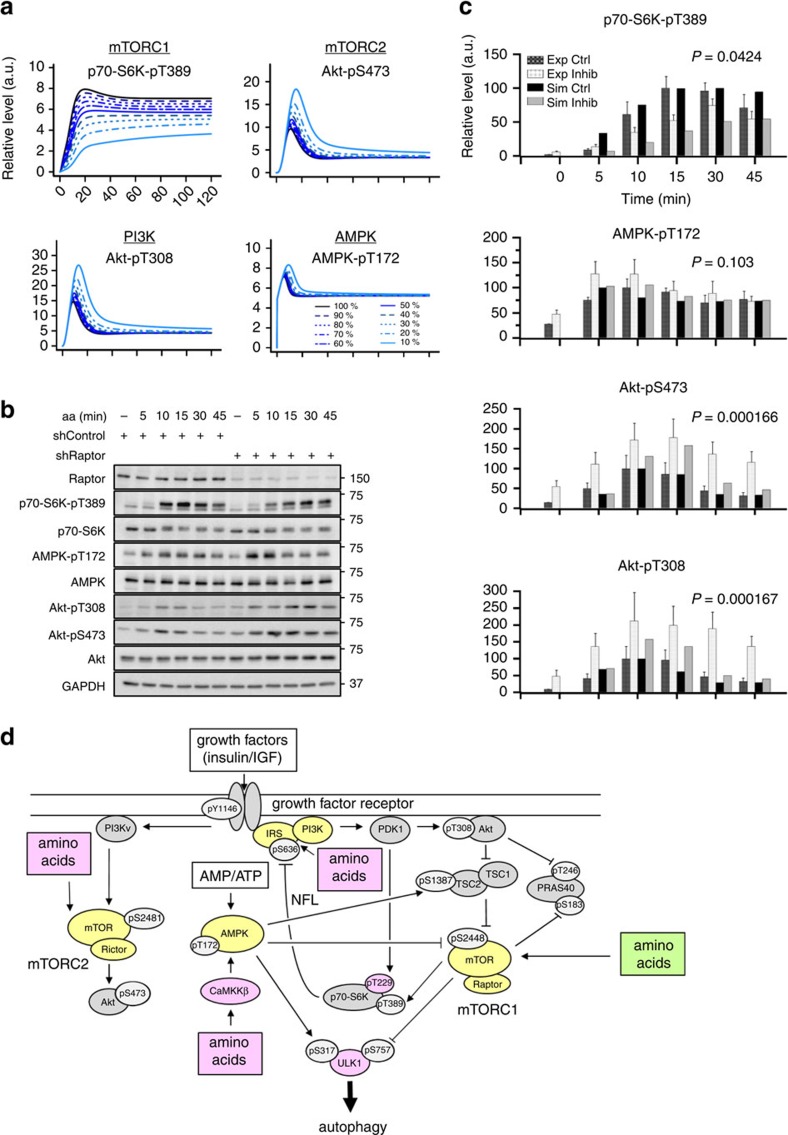
Amino acids (aa) stimulate the mTOR network via cues other than mTORC1. (**a**) Simulated response of p70-S6K-pT389, Akt-pS473, Akt-pT308 and AMPK-pT172 to aa stimulation in a system with mTORC1 perturbation (mTORC1 activity 10 to 100%; experimental equivalent: mTORC1 inhibition by Raptor knockdown). (**b**) Aa readdition enhances Akt-pT308, Akt-pS473 and AMPK-pT172 during mTORC1 inhibition by Raptor knockdown (shRaptor). Representative immunoblot results of aa-stimulated C2C12 cells with and without shRaptor (4 days). Data are representative of four experiments. (**c**) Quantitative representations of simulated (mTORC1 inhibition: reduction to 25%, as shown in **a**) and experimentally determined dynamics of p70-S6K-pT389, AMPK-pT172, Akt-pS473 and Akt-pT308 upon stimulation with aa with mTORC1 inhibition using shRaptor. Data are the mean and s.e.m., *N*=4. Statistical significance between control and treatment in experimental quantitations across the time course was detected by repeated measures analysis of variance (ANOVA). Exp Ctrl, experimental control condition (shControl); Exp Inhib, experimental mTORC1 inhibition (shRaptor); Sim Ctrl, simulated control condition; Sim Inhib, simulated mTORC1 perturbation. (**d**) Schematic representation of the mTOR-AMPK network with four aa inputs (known input green, new inputs magenta). Aa activate (1) mTORC1, (2) PI3K/PDK1, directly activating Akt and p70-S6K, (3) mTORC2 and (4) AMPK via CaMKKβ. Of note, AMPK activation by aa does not inhibit mTORC1, but activates ULK1. mTORC1 inhibits ULK1 in response to aa. Via these events, AMPK and mTORC1 balance ULK1 activity and autophagy under nutrient sufficiency.

Our model predicted that the aa-responsive components AMPK, PI3K and mTORC2 remain aa inducible when mTORC1 is inhibited (Fig. 7a). Our model also suggested that Akt phosphorylation by PDK1 (Akt-pT308) and mTORC2 (Akt-pS473), as well as AMPK activation are even enhanced under mTORC1 inhibition. To test this experimentally (Fig. 7b), we targeted Raptor by shRNA (shRaptor) before aa readdition. Consistent with our simulations, experimental mTORC1 inhibition reduced p70-S6K-T389 phosphorylation, and increased Akt-pS473 and Akt-pT308 as compared with control cells (Fig. 7b,c; Supplementary Fig. 25). Our simulations also predicted a moderate and transient activation of AMPK by mTORC1 inhibition at early time points (Fig. 7a) that we observed in our data at 5 and 10 min post-aa stimulation (Fig. 7b,c; Supplementary Fig. 25), although the effect over the whole time course was not statistically significant (Fig. 7c; Supplementary Fig. 25). For completeness, a comparison between the prediction of the model without p70-S6K-pT229 module, and experimental data on mTORC1 inhibition is provided (Supplementary Fig. 26), showing consistent results.

We confirmed our experimental results by a second means of mTORC1 inhibition using rapamycin. We found that this mTORC1 inhibitor also reduced p70-S6K-T389 phosphorylation, whereas AMPK-pT172, Akt-pS473 and Akt-pT308 remained responsive to aa (Supplementary Fig. 27a,b). In contrast to the Raptor knockdown (Fig. 7b,c), rapamycin did not enhance Akt phosphorylation in aa-stimulated cells. Hence, negative feedback from mTORC1 to Akt is present in aa-stimulated C2C12 cells, but it is rapamycin insensitive. This is consistent with earlier reports on rapamycin-resistant mTORC1 functions^39^,^40^. The fact that IRS1 is not required for PI3K and mTORC1 activation by aa (Fig. 3d) suggests that this negative feedback in aa-stimulated cells is mediated by molecules other than IRS1. The molecular mechanism that mediates negative feedback from mTORC1 to Akt under aa stimulation remains to be determined.

In summary, our data and simulations are consistent with a model, in which direct mTORC1 activation represents an important aa input into the mTOR network, which is supplemented by further, independent inputs activating AMPK, PI3K/Akt and mTORC2.

## Discussion

To date, the aa response of the mTOR network has been clearly assigned to mTORC1, via the Rag GTPases^5^, and several other molecular mechanisms directly impinging on mTORC1 (refs 2, 5, 30). In this study, we used computational modelling as a means to broaden the scope and seek other aa inputs to the mTOR network. There are several other systems modelling studies of the mTOR network (for example, Jain and Bhalla, 2009; ref. 41), but only one has attempted to model multiple aa inputs^42^ and found that a single input via mTORC1 was sufficient to explain a set of reported observations. Using time-course data only, our computational strategy for exploring alternative aa inputs allowed us to comprehensively cover model structures that showed an improvement to the canonical single aa-mTORC1 input. A total of 70 models were quantified and compared, offering an exhaustive analysis that provided hypotheses for new aa inputs. Attesting to the predictive power of our modelling approach, the model-derived hypotheses steered experiments that strongly supported the existence of the additional aa inputs into the mTOR network. Although the branch-n-bound-based approach taken here proved very effective, the large number of inputs we considered and the networks they generated were computationally challenging. The success of our approach depended on an effective strategy for model selection together with reliable and efficient tools for parameter estimation and identification. Discovery phosphoproteomics complemented by computational large-scale text mining strategies proved a powerful means to expand the predictions arising from our small-scale dynamic model simulations to an omics-wide level and to systematically cover the full body of available scientific literature. The issue of how to expand small-scale dynamic modelling approaches to an omics-wide level is one of the major challenges in systems biology. Genome scale models allow to deal with large data sets, but do not offer the level of detail necessary for dynamic signalling network analyses as done in this study.

Our final computational model predicted additional, independent aa inputs to the network via AMPK, mTORC2 and class I PI3K upstream of Akt and mTORC1 (Fig. 7d). In the light of mTORC1 being considered as the central p70-S6K activator upon aa, we were surprised by the strong, direct dependence of p70-S6K-pT389 on PI3K/PDK1 (Fig. 1). The requirement of this link for the reproduction of the measured data by our model highlights the critical importance of the PDK1 input to p70-S6K in response to aa. In support of this notion, a recent study has reported the requirement of PDK1 for mTORC1 and p70-S6K activation by leucine in cardiac myocytes^43^.

Strikingly, AMPK responded acutely and within 1 min to aa, and its activation was independent of the other aa-responsive components mTORC2, PI3K and mTORC1. This suggests aa as novel metabolic activators of AMPK. This finding was unexpected because AMPK is mainly considered to be activated by stress and starvation conditions, including aa withdrawal^44^ although there is conflicting data^45^. This may in turn imply that aa exert an inhibitory effect on AMPK, which has indeed been suggested by several studies^46^,^47^,^48^. It should be noted though that all these studies observed AMPK phosphorylation at the earliest at 15 min post aa addition. At this time point we also observed AMPK repression (Supplementary Fig. 2b), and this is why the earlier studies may have missed the early AMPK inducibility by aa.

How is the aa signal transduced to AMPK? LKB1 is the most commonly reported activating kinase for AMPK that phosphorylates AMPK in the activation loop at T172 (ref. 15). However, AMPK responded to aa not only in C2C12, but also in HeLa cells (Fig. 5f) that lack LKB1 (refs 49, 50). Therefore, we tested the impact of CaMKKβ, another kinase for AMPK^15^, which had been shown in an earlier study to mediate AMPK phosphorylation in response to the glutamate agonist homocysteine sulfinic acid^45^. We found indeed that CaMKKβ inhibition prevented AMPK activation by aa. Of note, CaMKKs have also been shown to act as upstream regulators of the Akt pathway in non-muscle cells^51^, but this was not the case in aa-stimulated C2C12 cells (Fig. 5d,e). The CaMKKβ mediated AMPK activation involves an increase in intracellular Ca^2+^ without requiring a change in AMP or ADP levels^52^. Ca^2+^ release typically happens on very short time scales, which is in agreement with the observed rapid response of AMPK to aa. Aa have been reported to allosterically activate extracellular calcium receptor (CaR) proteins^53^. CaR belong to the G-protein-coupled receptor family. CaR are able to activate intracellular signalling molecules, including cytosolic phospholipase A2 (ref. 54) and mitogen-activated protein kinases^55^ via CaMKKβ. Thus, AMPK may be another kinase that is activated by aa-sensitive CaR via CaMKKβ.

Our data suggest that activation of the CaMKKβ-AMPK axis by aa does not inhibit mTORC1 (Fig. 5d,f,g), but sustains ULK1 activity and autophagy (Fig. 5g,j). Likewise, c-Jun, reported to respond to AMPK^56^ and to inhibit autophagy^38^, was phosphorylated upon aa readdition (Fig. 6d), and hyperphosphorylated when CaMKKβ was inhibited (Fig. 6e). Aa readdition activates mTORC1-dependent translation^57^, which is generally perceived as being associated with an inhibition of autophagy^58^. Why may cells activate autophagy in response to nutrient readdition? It has been noted recently that activation of biosynthetic pathways may require autophagy for several reasons. Firstly, increased protein synthesis inevitably also increases the amount of misfolded proteins^59^, and autophagy contributes to their clearance^60^. Secondly, the upregulation of biosynthetic processes requires increased amounts of metabolite intermediates, which are provided by autophagy as well^16^. Consistent with this, a previous study described active autophagy in the context of active mTORC1 signalling^61^.

In this study, we have analysed the global effect of a mixture of aa on the mTOR signalling network. Thus, we did not differentiate between the relative contributions of different aa to AMPK activation. Future efforts will need to focus on the relative contribution of different aa to this event. Here, the branched-chain aa leucine, as well as glutamine and arginine deserve particular attention, as they all have been identified as being important in the regulation of the mTOR network^5^,^62^,^63^.

## Methods

### Cell lines and tissue culture

Experiments were performed in C2C12 myoblasts (obtained from the department of Ralf Baumeister, University of Freiburg, D^4^,^19^), HeLa α Kyoto cells (obtained from Cecile Arrieumerlou, University of Basel, CH^64^) and Ulk1/2 DKO MEF cells (kindly provided by Tullia Lindsten, Memorial Sloan Kettering Cancer Center, New York City, USA) overexpressing FLAG-ULK1-WT or FLAG-ULK1-S758E. All cells were tested every 3 months for mycoplasma contamination by performing a PCR on the cell supernatant^65^. For retroviral infection of MEFs, human complementary DNAs encoding either FLAG-tagged full-length ULK1 or an ULK1-S758E mutant were cloned into pMSCVpuro (Clontech Laboratories, Takara Bio, 631461). For the production of recombinant retroviruses, Plat-E cells (kindly provided by Toshio Kitamura, Institute of Medical Science, University of Tokyo, Japan) were transfected with 1.9 μg pMSCVpuro-based retroviral vectors using FuGENE 6 transfection reagent (Roche, 11988387001). Ulk1/2 DKO MEF cells^66^ were incubated with retroviral supernatants containing 9 μg ml^−1^ Polybrene (Sigma-Aldrich, H9268-106) and were selected in medium containing 2.5 μg ml^−1^ puromycin (InvivoGen, ant-pr-1). For inducible knockdown of mSin1, C2C12 cells were transduced with lentivirus encoding the specific shRNA (target sequence: 5′-TAATCCAAAACTTAGTGCT-3′) on the tetracycline-sensitive pTRIPZ vector (clone V3THS_375315) obtained from Dharmacon Thermo Fisher Scientific, Waltham, MA, USA. C2C12 cells were transduced with lentivirus according to the manufacturer's instructions. Knockdown was induced with 2 μg ml^−1^ doxycycline (Calbiochem, Merck, Darmstadt, Germany) for 4 days. For knockdown of Raptor, a lentiviral plasmid was obtained from Addgene (pLKO mouse shRNA 2 raptor, #21340, target sequence: 5′-CCGGGCCCGAGTCTGTGAATGTAATCTCGAGATTACATTCACAGACTCGGGCTTTTTG-3′). C2C12 cells were transduced with viral supernatant twice. Cells were collected 4 days after the first transduction. AMPKα1/2 siRNA (human, sc-45312) was purchased from Santa Cruz Biotechnology, Santa Cruz, CA, USA and IRS1 siRNA (mouse, L-040503-02-0005) was purchased from GE Healthcare, Dharmacon. HeLa α Kyoto cells were transfected with 10–20 nM siRNA and Lipofectamine 2000 (Life Technologies, Carlsbad, CA, USA) for 48 h according to the manufacturer's instructions.

### Antibodies and reagents

The anti-GAPDH monoclonal antibody (AB8245) was obtained from Abcam, Cambridge, UK and used at a final dilution of 1:10,000 in TBST (tris-buffered saline-Tween) buffer (138 mM sodium chloride, 2.7 mM potassium chloride, 66 mM tris (pH 7.4), 0.1% Tween-20) with 5% bovine serum albumin (BSA). BSA (K45-001) was obtained from GE Healthcare. The antibody recognizing insulin receptor β (sc-711) was purchased from Santa Cruz Biotechnology and used at a final dilution of 1:1,000 in TBST buffer with 5% BSA. Horseradish peroxidase conjugated goat anti-mouse (#31430) and goat anti-rabbit (#31460) IgG were obtained from Pierce Biotechnology (Thermo Fisher Scientific, Rockford, IL, USA) and used at a final concentration of 0.1 μg ml^−1^ in TBST buffer with 5% BSA. The rabbit polyclonal antibody against p70-S6K (phospho T229, GTX25231-50) was obtained from Biozol, Eching, Germany and used at a final dilution of 1:1,000 in TBST buffer with 5% BSA. All other antibodies were obtained from Cell Signaling Technology, Danvers, MA, USA as described previously^4^,^19^. In addition, ULK1-pS371 (#6887), ULK1-pS757 (#6888), ULK1 (#8054), c-Jun-pS63 (#9261), c-Jun-pS73 (#9164), c-Jun (#9165), S6-pS235/236 (#4856) and S6 (#2317) antibodies were purchased from Cell Signaling Technology. All antibodies from Cell Signaling were used at a final dilution of 1:1,000 in TBST buffer with 5% BSA. Rapamycin (R8781) and wortmannin (W1628) were supplied by Sigma Aldrich, St. Louis, MO, USA. MK-2206 (S1078) was purchased from Selleckchem, Houston, TX, USA. STO-609 (sc-202820) was purchased from Santa Cruz Biotechnology. BafA (#88899-55-2) was supplied by bioaustralis, Smithfield, NSW, Australia. Chemicals were purchased from Carl Roth, Karlsruhe, Germany if not indicated differently.

### Analysis of cell lysates

Cells were starved, stimulated and lysed as described previously^19^. In brief, cells were starved for aa and growth factors in HBSS for 16 h before all stimulation experiments. Cells were stimulated with DMEM containing aa and supplemented with 100 nM insulin (I1882, Sigma Aldrich) or with DMEM only, as indicated. For HBSS+aa stimulation experiments, different aa mixtures were obtained from Sigma Aldrich (catalogue number aa mix 1: R7131 and aa mix 2: M5550) and added 1:50 to HBSS medium. In addition, L-glutamine was added to a final concentration of 3 mM (#25030081, Gibco, Life Technologies). Cells were lysed in RIPA lysis buffer (150 mM sodium chloride, 50 mM Tris [pH 8.0], 1% NP40, 0.5% sodium deoxycholate, 0.1% SDS) supplemented with Complete (#11836145001, Roche, Mannheim, Germany), Phosphatase Inhibitor Cocktail 2 (P5726), Phosphatase Inhibitor Cocktail 3 (P0044) (both Sigma Aldrich). Rapamycin (100 nM), wortmannin (100 nM), MK-2206 (1 μM) or STO-609 (10 μg ml^−1^) were added 30 min before and during the stimulation with DMEM. BafA (100 nM) was added 60 min before and during stimulation with DMEM. Inhibitors were dissolved in dimethyl sulfoxide (DMSO), and controls were treated with DMSO only.

Pierce enhanced chemiluminescence (ECL) western blotting substrate (#32209) or SuperSignal West Femto (#34095), both Thermo Scientific Pierce were used to detect chemiluminescence using a LAS-4000 mini camera system (Fujifilm Life Science Systems; Figs 1c, 4b, 5b, 5f and 7b; and Supplementary Figs 2 and 27a) or LAS-4000 mini camera system (GE Healthcare; all other figures). To detect several proteins of different molecular weights on one membrane, the molecular weight of each protein was determined using the protein all blue standard marker (#161-0393, Bio-Rad, California, USA), and membranes were cut accordingly. The uncropped immunoblots for all figures and Supplementary Figures are shown in Supplementary Figs 28–39.

For graphical representation, raw images taken with the Fujifilm camera were exported as colour TIFF files using the Fujifilm software Multi-Gauge version 3.0, Fujifilm Life Science Systems, and further processed with Adobe Photoshop version CS2. For quantitation, the raw images were analysed with Multi-Gauge version 3.0 software (Fujifilm). Local background was subtracted.

Raw images taken with the LAS-4000 mini system were exported as red green blue (RGB) colour TIFF files using ImageJ, and further processed with Adobe Photoshop version CS5.1. Quantitation of raw image files was performed using ImageQuant TL Version 8.1, GE Healthcare. The rolling ball method was used for background subtraction, and the radius was set to 200.

The obtained values were normalized to the control condition and loading control. The mean and the s.e.m. were plotted and statistics calculated as described below.

### Mathematical model

The final model including the extended p70-S6K module consisted of 31 species and 48 mass-action reactions and was built using ordinary differential equations (ODEs). The species and reactions described the phosphorylation and dephosphorylation protein states of the following modules: IR, IRS, PI3K, Akt, AMPK, TSC1–TSC2, PRAS40, mTORC1, mTORC2 and p70-S6K. The initial protein amount of the unphosphorylated states was fixed a *priori* to a value that avoided phosphorylation level saturation, whereas the initial amount for the phosphorylated states was fixed to 0. The low basal network activity under starvation is neglected by this simplification. The model was linked to immunoblot experimental data by 12 observable variables (Supplementary Tables 1 and 5) covering all the network. The observable variables used for model parameter estimation and as readouts (model output) throughout the manuscript are indicated with the suffix ‘obs' in the model files. For the species Akt, PRAS40 and p70-S6K, the observables represented the total phosphorylation level of the corresponding phosphosite (for example, Akt-pS473-obs was associated to the sum of the species Akt-pS473 and Akt-pT308-pS473, whereas Akt-pT308-obs was linked to the sum of the species Akt-pT308 and Akt-pT308-pS473). Although this design represented all Akt, PRAS40, and p70-S6K phosphorylation states, its expansion was combinatorial in the number of states, adding complexity to the parameter estimation and identification. Using an approach similar to rule-based modelling^67^, we constrained the parameter values of the corresponding first and second reactions (for example, the parameter Akt-pS473-dephos-first, which regulated the dephosphorylation from the state Akt-pS473 to Akt, was constrained to be equal to the parameter Akt-pS473-dephos-second, which regulated the dephosphorylation from the state Akt-pT308-pS473 to Akt-pT308). This process allowed us to express all the internal states of Akt and PRAS40, but maintained a low number of estimated parameters. A legend of all the names (for example, species, parameters, observables and so on) used in the model is provided in Supplementary Tables 1 and 5. A complete list of ODEs is provided in Supplementary Tables 2, 3 and 6.

The Matlab Toolbox PottersWheel^68^ and the PottersWheel identifiability toolboxes MOTA^69^ and PLE^70^ were used for designing and calibrating the kinetic rate constants of the models. Each reaction contained a kinetic rate constant parameter that was estimated within the range (1e−06, 1e+04). The insulin receptor module (three parameters) was independently calibrated by executing two rounds of combined parameter estimation and MOTA identifiability analysis (Supplementary Table 4; Supplementary Fig. 4). Each round consisted in up to 2,500 independent fits with a parameter disturbance noise of 0.4. For each fit a maximum of 250 iterations with *χ*^2^ and parameter tolerances of 1e−05 were run using the TrustRegion optimization algorithm. For each round, parameters were considered as non-identifiable when their correlation coefficient of the parameter tuple and the coefficient of variance were >15% and 0.9%, respectively. Since the s.e. of the experimental time points was often large, a 10% error model of observation was adopted solely for the task of parameter estimation. This improved the approximation of the model versus the data. CVODES integrator was selected and configured with the following parameters: maximum number of steps, 1,500; relative tolerance, 1e−06; and absolute tolerance, 1e−08. MOTA was configured with the parameters: percentageBestFits, 50; minimumPValue, 0.001; percentageOutliers, 100; maxNumberOfParameters, 5. Once the IR was calibrated, the networks (from 32 to 35 parameters) for the 69 model variants, including additional aa inputs, were estimated maintaining the previous algorithm configuration. To reduce the computation time of this task, a cluster of six GNU/Linux computers for a total of 60 cores was adopted. For the model including the aa input connections of mTORC1, mTORC2, AMPK and IRS, and the simplified p70-S6K module (Supplementary Tables 1–4), we estimated and identified the parameters by iterating up to eight rounds of alternated parameter estimation and MOTA identifiability analysis (Supplementary Table 4; Supplementary Figs 4–12). Due to the difficulty of measuring PI3K dynamics experimentally, the PI3K module was not associated to any observable data. Therefore, the parameters describing this protein complex were assumed during the calibration of the model, including a simplified p70-S6K module, to limit parameter non-identifiability. We opted to set these parameter values so that the time course profile for PI3K was close to that for IR. This model was then enhanced to incorporate a more accurate design for the p70-S6K module. To limit the computation time and still achieve an accurate solution, we did not estimate the model entirely, but rather used most of the results from the previous computations (IR and eight additional calibration rounds Supplementary Table 4), but estimated all the eight parameters defining the new p70-S6K module and re-estimated the main four parameters affected by the NFL (IRS, PI3K). The estimation of the parameters for PI3K was considered at this stage to further improve the p70-S6K-dependent NFL. As again, we did not need to re-estimate all the parameters the solution space was sufficiently constrained and limited. In fact, the parameters for PI3K were not completely free as would have been the case for the original calibrations of the rounds 1–8, but were indirectly constrained to the dynamics of Akt, IR and IRS, whose parameters were fixed. This approach allowed us to estimate all these 12 parameters within two additional rounds of parameter estimation.

PLE identifiability analysis was performed a posteriori based on the parameter values of this final model. Almost all the parameters were structurally and practically identifiable except for eight parameters labelled as practically non-identifiable (Supplementary Tables 4 and 7). However, we observed that these either involved parts of the network of secondary importance to our study, such as the refractory state of the IR or PRAS40-T246, or phosphatases for which we did not have any measurement, such as the dephosphorylation of Akt-pT308 or TSC2-pT1462. In addition to the final value of each parameter, we also computed the mean and s.d. from the selected round fits, to assess parameter variability in relation to the model and not only to individual parameter perturbation. In conclusion, we provided evidence that all the model components required for determining our predictions are identifiable.

### Modelling Tasks

Copasi 4.8.35 (ref. 71) was used for simulating the inhibition experiments for 10 levels of protein from 10 to 100% (control) for mTORC1, mTORC2, PI3K and AMPK. As the total level of these proteins remains constant across the time course, this was achieved by modifying initial amounts. Simulations were calculated by running a deterministic algorithm (LSODA), which was configured with the following parameters: duration, 120; interval size, 0.1; intervals, 1,200; integrate reduced model, 0; relative tolerance, 1 × 10−6; absolute tolerance, 1 × 10−12; and maximum internal steps, 10,000. PottersWheel was used to export the models in SBML format^72^ Level 2 Version 4. CellDesigner 4.3 (ref. 73) was used to construct the model network topology in systems biology graphical notation (SBGN)^74^.

### Quantitative SILAC-based phosphoproteomics

Two triple-SILAC approaches were combined to analyse the phosphoproteome of C2C12 cells upon aa stimulation for 0–30 min. Cells were starved for aa for 16 h, before aa readdition. The first approach covered 0, 5 and 30 min aa stimulation. For the second approach 10, 15 and 30 min time points were chosen. Measurements at the 0 min time point (that is, no aa readdition) were used as a reference point for no induction. The fact that the 30 min time point was used for both settings allowed the normalization to this time point and thus a quantitative phosphoproteome analysis across five time points. C2C12 cells were cultivated in SILAC media containing either non-labelled arginine and lysine (light), ^13^C_6_-arginine/D_4_-lysine (medium) or ^13^C_6_^15^N_4_-arginine/^13^C_6_^15^N_2_-lysine (heavy) for at least five passages (1:10), then starved for aa and growth factors for 16 h followed by a stimulation with aa for five different time points. Cells were lysed in sodium deoxycholate lysis buffer and lysates were mixed 1:1:1 followed by tryptic digestion using a trypsin-to-protein ratio of 1:50 at 37 °C for 6 h. In total, five experiments were performed with label switches between the various time points to avoid bias toward a particular label. Zero and 5 min time points included three biological replicates, 10 and 15 min included two biological replicates, while the time point 30 min was included in all experiments and served as normalization. The experimental design of the proteomics study is outlined in the legend sheet of Supplementary Data 1 and 2. The distribution of the SILAC ratios for the data sets acquired at 5, 10 and 15 min of aa stimulation is presented in Supplementary Fig. 24a. The SILAC ratio distribution was highly similar across all three time points, as only a small fraction of all measured phosphosites changed in abundance, whereas the central part remained constant.

Strong cation exchange chromatography and titanium dioxide enrichment were essentially performed as described^75^,^76^. In brief, 30 fractions a 2.1 ml were collected throughout the strong cation exchange chromatography gradient. A measure of 20 μl of each fraction were directly used for liquid chromatography/MS analysis, while the major part of the fractions was subjected to titanium dioxide enrichment. All samples were desalted as described^77^. Liquid chromatography/MS analysis was performed on an LTQ Orbitrap XL (Thermo Fisher Scientific, Bremen, Germany) either coupled to an Agilent 1200 or an Eksigent 2D nanoflow-high-performance liquid chromatography equipped with in-house packed C18 columns of about 20 cm length (Reprosil-Pur 120 C-18-AQ, 3 μm, Dr Maisch, Ammerbuch, Germany) and no pre-column was used. Peptides were separated using a binary solvent system consisting of buffer A (0.5% acetic acid) and buffer B (0.5% acetic acid and 80% acetonitrile). Samples were loaded in 2% buffer B with a flow rate of 500 nl min^−1^ and separated by a gradient from 2 to 35% buffer B in 100 min, followed by a gradient from 35 to 80% in 20 min with a flow rate of 250 nl min^−1^.

Mass spectrometric measurements were performed in the data-dependent mode. The spray voltage was 2.3 kV with no sheath or auxiliary gas flow. The ion-transfer tube temperature was set to 200 °C. MS spectra in the range of *m/z* 350–2,000 were acquired using an automatic gain control of 1 × 10^6^. The five most abundant multiply charged peptides were selected for fragmentation in the linear ion trap using 35% collision energy and an automatic gain control of 5,000. Multistage activation was enabled with a list for neutral losses of one, two and three phosphoric acid masses. Dynamic exclusion of previously selected *m/z* values was enabled with a duration of 45 s and an isolation width of 2.

Raw MS files were processed using MaxQuant 1.3.0.0 (refs 78, 79) and the UniProt mouse protein sequence database (2013.10, containing 51,219 protein sequences)^80^. Parameters of MaxQuant processing and raw and processed files were uploaded to ProteomeXchange^81^ (identifier: PXD003073).

The MaxQuant protein and modified peptide identification summary was obtained from the ‘modificationSpecificPeptides.txt' MaxQuant output file, and the quantitative phosphorylation sites data from the 'Phospho(STY)Sites.txt‘ file, which have been uploaded to ProteomeXchange (see Data availability). MaxQuant output files are provided in Supplementary Data 4. These data were processed with a custom Matlab script that was submitted together with the raw and processed files to ProteomeXChange. The significantly regulated phospho(STY)sites and phosphopeptides resulting from the Matlab processing are presented in Supplementary Data 1 and 2, respectively. In brief, the Matlab script calculated all possible ratios between 5, 10 and 15 min as nominator and 0 min as denominator leading to nine and six ratios for time points of 5, 10 and 15 min, respectively, using the intensity columns of the three label variants according to the experimental design (see experimental design description in Supplementary Data 1and 2). All log_2_ ratios were corrected with the log_2_ ratios median and the entries with at least two existing or non-zero ratios were selected for Volcano filtering. The fold change and one sample *t*-test with null hypothesis that log_2_ fold ratio is 0 were calculated for the filtered data for Volcano plot visualization. Filtering of the data was performed with threshold of 1.5 and 2 for fold change and *t*-test *P*-value of 0.05 without correction for multiple testing. In data obtained by MaxQuant, we assumed that phosphosites with a probability >0.75 are correctly localized.

### Text mining

Supplementary Data 1 (phosphosites) and 2 (phosphopeptides) list the twofold-regulated phosphoproteins for which known molecular links (termed ‘molecular events') with AMPK were sought. Molecular events comprise protein production and breakdown, localization, binding as well as positive and negative regulatory events as defined in the BioNLP Shared Task 2011 on Event Extraction^82^. To mine the scientific literature in an automated manner, Medline as well as the open access subset of PMC were linguistically preprocessed. As of December 2015, Medline comprised 24 million abstract entries and the open access subset of PMC 1 million full-text entries.

The tools utilized for linguistic preprocessing were taken from the JCore repository, and comprise segmentation of text into basic units (sentences and words), acronym recognition and grammatical analysis required for subsequent text mining tools (for example, part of speech tagging)^83^. Next, a semantic analysis to correctly identify the occurrence of genes and proteins in texts, as well as molecular events between those was carried out. The identification of gene and protein name occurrences, as well as the assignment of an appropriate UniProt identifier was accomplished by the GeNo tool^84^. Using UniProt IDs for each identified gene or protein name ensured the unique and canonical identification of the referenced entity and thus served as a disambiguation facility. This normalization step was necessary, because the same name may be used to denote a set of distinct genes or proteins. Before conducting the analysis for events, the Medline and PMC databases were filtered to allow for efficient computability. The filtering was done by identifying those Medline abstracts that contained either an occurrence of AMPK or an item of Supplementary Data 1. The respective PubMed-IDs were mapped to PMC identifiers using the NCBI E-utilities^85^ to add available full texts to the set of relevant documents. This ensured the identification of relevant events even in the absence of one event partner in the respective abstract. Identifying any event between genes and proteins was finally accomplished by employing the BioSem tool^86^.

Given the BioSem-based reported event set, items that expressed an event between AMPK and an element of Supplementary Data 1 were filtered. To achieve this, the initial 204 distinct UniProt IDs delivered by the FASTA entries/Supplementary Data 1 were mapped to their associated gene names by the mapping tool ‘UniProt mapping service' provided at the UniProt website ( http://www.uniprot.org/mapping/). These gene names, in turn, were subsequently mapped to all associated UniProt identifiers for *Homo sapiens* and *Mus musculus* proteins. This resulted in an extended list of 2,201 UniProt entries (Supplementary Data 3, text mining input). For AMPK all UniProt IDs referring to one of its subunits were collected (Supplementary Data 3, text mining input).

The final filtering was consequently accomplished by restricting the BioSem-based event set to those, where an AMPK identifier and an identifier of the extended Supplementary Data 1 and 2 were included (Supplementary Data 3).

### Statistics

The number of biological replicates for each experiment is indicated in the figure legends or methods section. The statistical and programming language R version 2.15.1 (ref. 87) was used to calculate the statistics and generate the model perturbation plots, except for Fig. 5i and Supplementary Fig. 23, where GraphPad Prism Version 5.00 was used to display the quantitations. The s.e.m. was chosen to estimate the statistical variability of the measured samples of experimental time course. Statistical significance between control and treatment in laboratory experimental quantifications was detected by repeated measures analysis of variance (ANOVA) test implemented in R. Model goodness of fit was computed using AIC^27^ as implemented in PottersWheel. Finally, R was also used for the graphic representation of the identifiability matrix computed with PottersWheel plugin MOTA.

### Data availability

The proteomics data have been deposited to the ProteomeXchange Consortium via the PRIDE partner repository with the data set identifier PXD003073. Other data on which the conclusions of this study are based are available from the corresponding authors upon request. The three models generated as a part of this study are provided as Supplementary Softwares: Supplementary Software 1: SBML model including only the canonical aa input on mTORC1. Supplementary Software 2: SBML model including four aa inputs in the network (simple p70-S6K module). Supplementary Software 3: SBML model similar to Model S2, but including a more complex p70-S6K module.

## Additional information

**How to cite this article**: Dalle Pezze, P. *et al*. A systems study reveals concurrent activation of AMPK and mTOR by amino acids. *Nat. Commun.* **7**, 13254 doi: 10.1038/ncomms13254 (2016).

**Publisher's note:** Springer Nature remains neutral with regard to jurisdictional claims in published maps and institutional affiliations.

## Supplementary Material

Supplementary Information Supplementary Figures 1-39, Supplementary Tables 1-7, Supplementary References.

Supplementary Data 1 List of differentially regulated phosphosites. Phosphosites showing fold ratio larger than 2 or 1.5 fold changes (FC) and p-values lower than 0.05 between time points 5 minutes, 10 minutes and 15 minutes after amino acid readdition compared to the starting time (0 minutes).

Supplementary Data 2 List of differentially regulated phosphopeptides. Phosphopeptides showing fold ratio larger than 2 or 1.5 fold changes (FC) and p-values lower than 0.05 between time points 5 minutes, 10 minutes and 15 minutes after amino acid readdition compared to the starting time (0 minutes).

Supplementary Data 3 Text mining input and results for the detection of molecular event partners of AMPK reported in scientific texts (Medline and PubMed Central). Genes and proteins were mapped to their respective UniProt ID to avoid ambiguity. The event partners as well as the textual contexts of the events themselves are listed.

Supplementary Data 4 Phosphoproteomic identification data. Contains excerpts from the output files "proteinGroups" including information on protein group identification and quantification, "peptides" including information about peptide identification and quantification and "PhosphoSTY" containing information about phosphopeptide identification and quantification as well as on phosphorylation site localization.

Supplementary Software 1 SBML model including only the canonical amino acid input on mTORC1.

Supplementary Software 2 SBML model including four amino acids input in the network (simple p70-S6K module).

Supplementary Software 3 SBML model similar to Supplementary Model 2, but including a more complex p70-S6K module.

## Figures and Tables

**Table 1:**
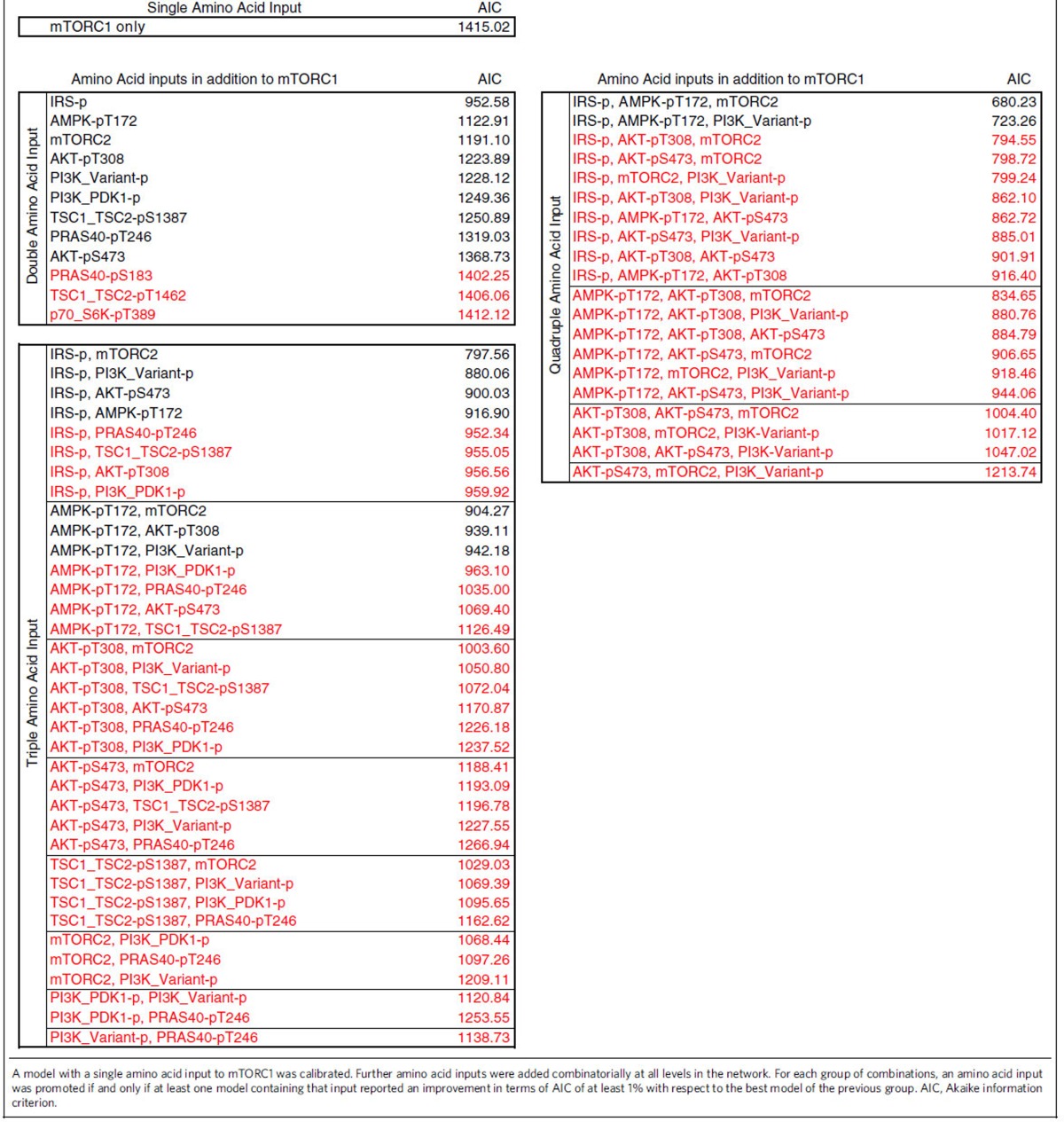
Model prediction of multiple amino acid inputs.
