# Supplementary material for: A systems study reveals concurrent activation of AMPK and mTOR by amino acids
Source: Nat Commun. 2016 Nov 21;7:13254. doi: 10.1038/ncomms13254 (PMC5121333; doi:10.1038/ncomms13254)
Supplement: Supplementary Information — Supplementary Figures 1-39, Supplementary Tables 1-7, Supplementary References. [file ncomms13254-s1.pdf]

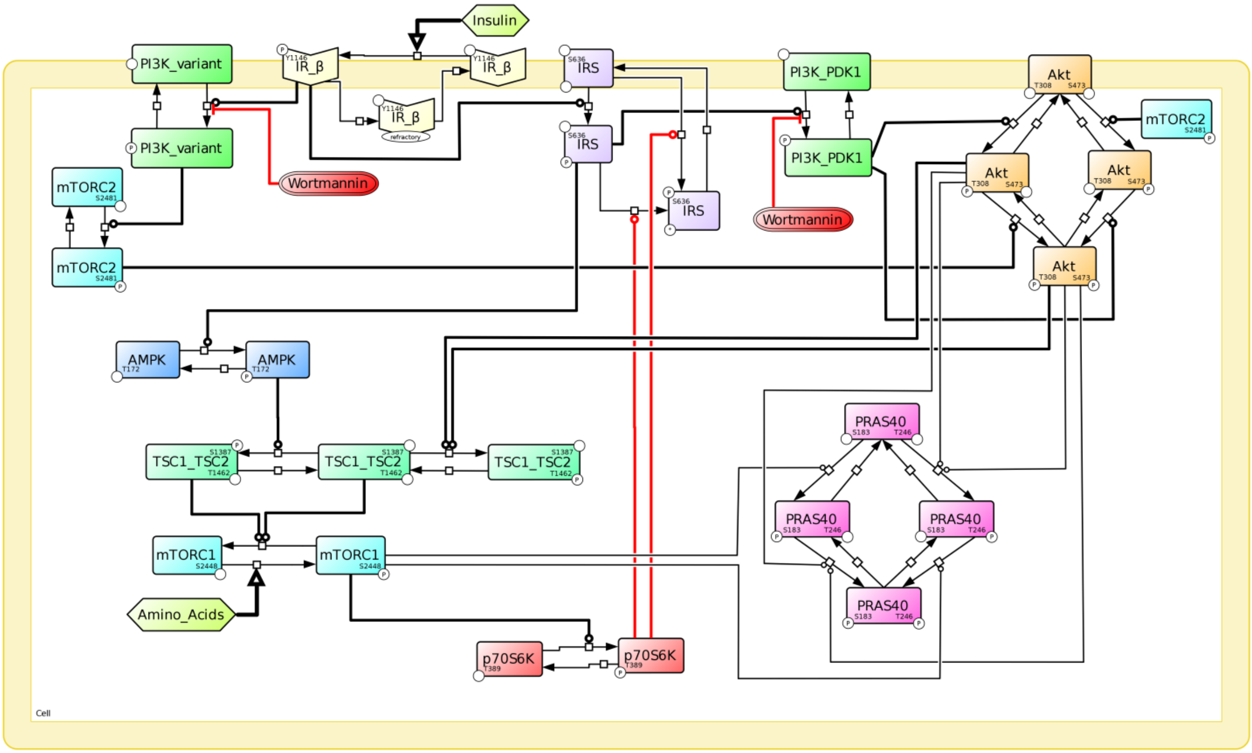

Supplementary Figure 1. The mTOR-AMPK network

Graphical model including only the canonical amino acid input on mTORC1.This model represented the default structure from which all the alternative hypotheses had been defined and evaluated. In particular, the aa-input only affected mTORC1, abstracting the Rag GTPase pathway. The red connections from p70-S6K to IRS represent the negative feedback loop (NFL).

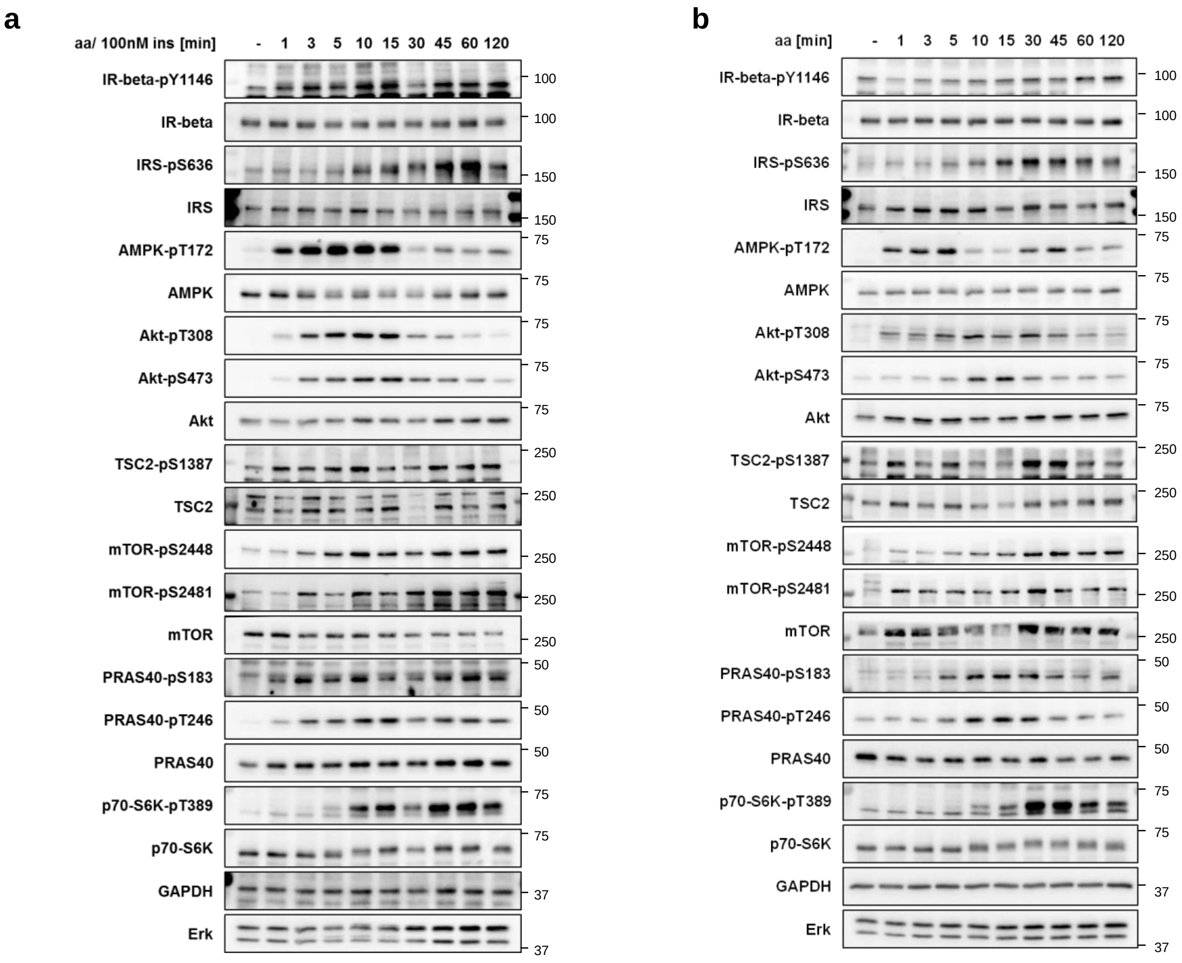

Supplementary Figure 2. Immunoblot data used for calibrating the model

(a) Dynamic semi-quantitative time course data acquisition. mTOR network activation by amino acids (aa) and 100 nM insulin was followed over time by measuring phosphorylation dynamics of central network components. A representative experiment of three biological replicates is shown. (b) Dynamic semi-quantitative time course data acquisition. mTOR network activation by aa alone. A representative experiment of three biological replicates is shown.

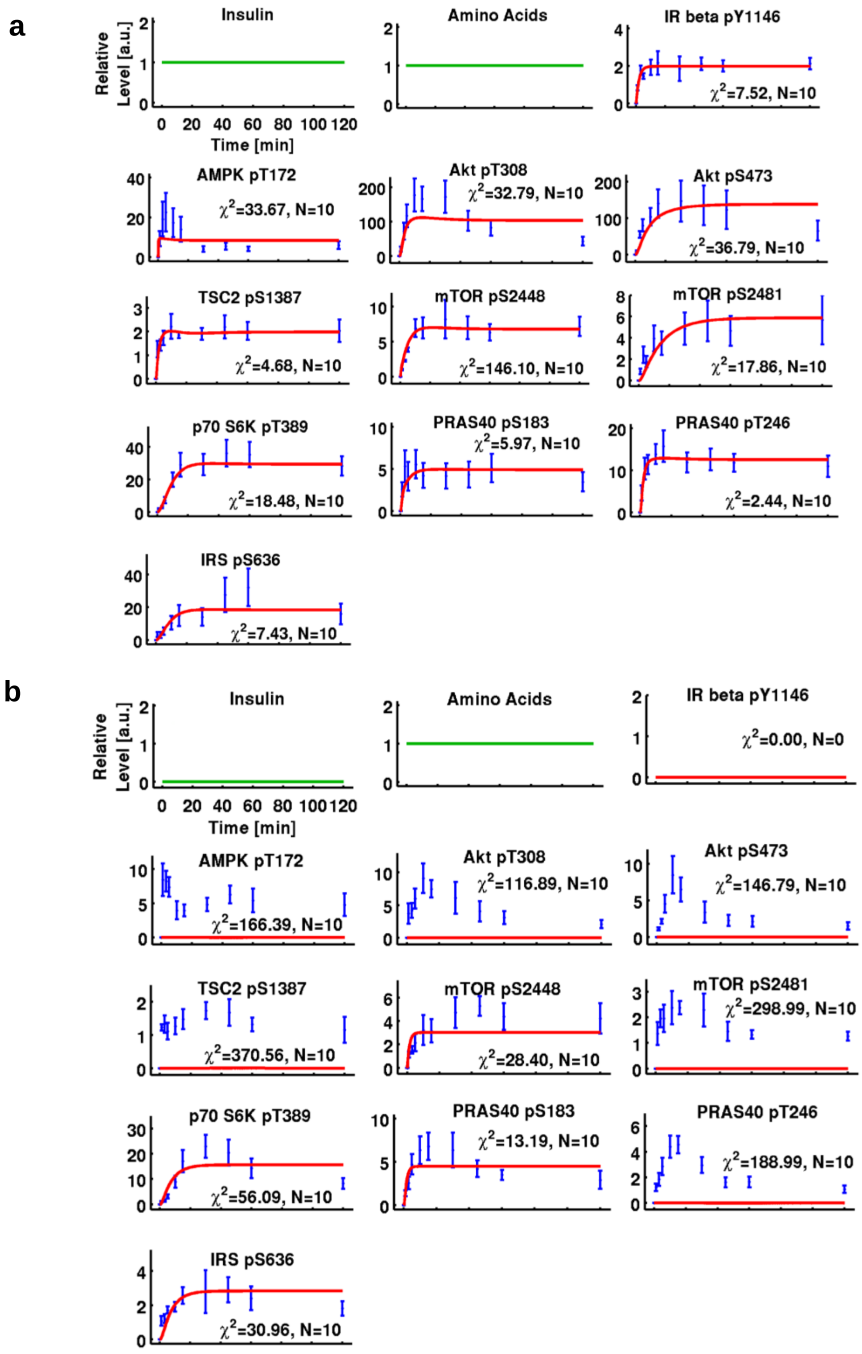

Supplementary Figure 3. Time courses for the model including only the canonical amino acid (aa) input on mTORC1

The canonical model shown in **Supplementary Fig. 1** was calibrated using two datasets: (a) aa + insulin and (b) aa only (see immunoblot data in **Supplementary Fig. 2**). Although it was possible to fit the first data set (a), a complete parameter estimation including both data sets proved impossible (b). In fact, the model was not able to fit the experimental data for AMPK-pT172, TSC2-pS1387, Akt-pT308, Akt-pS473 and PRAS40-pT246. This indicated missing regulations upon aa-only stimulation. Depicted are simulated time courses (solid lines) and the experimental time courses (points and dotted error bars represent mean and SEM) within [0, 120] min.

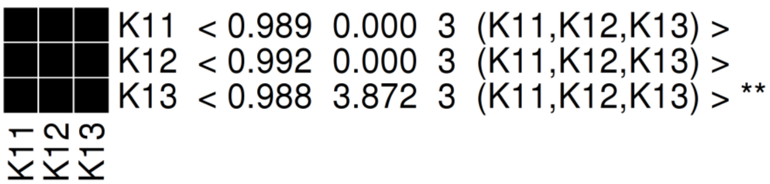

Supplementary Figure 4. MOTA identifiability matrix of the first calibration round for the insulin receptor

At the first round the kinetic rate constants K11 and K12 were fixed since identifiable using MOTA identifiability analysis. * : Correlation Coefficient (CC) > 0.9 and Coefficient of Variation (CV) > 0.15; ** : Correlation Coefficient (CC) > 0.9, Coefficient of Variation (CV) > 0.15 and number of tuples showing this correlation (#) > 1. Format: ParameterCode < CC CV # (“Tuple of related parameters”)>.

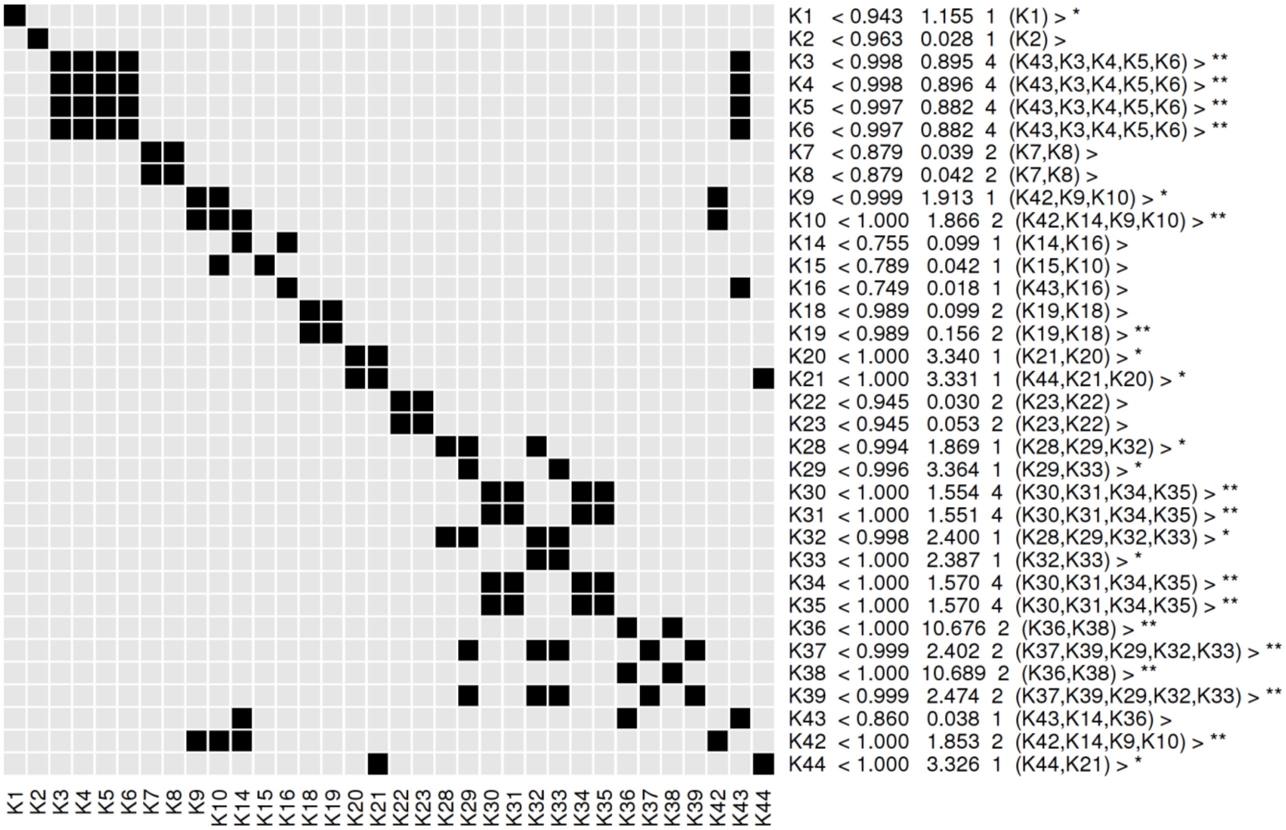

Supplementary Figure 5. MOTA identifiability matrix of the 1st calibration round for the complete model

In this round 10 parameters were fixed as reported in **Supplementary Table 4**. See **Supplementary Fig. 4** for legend description.

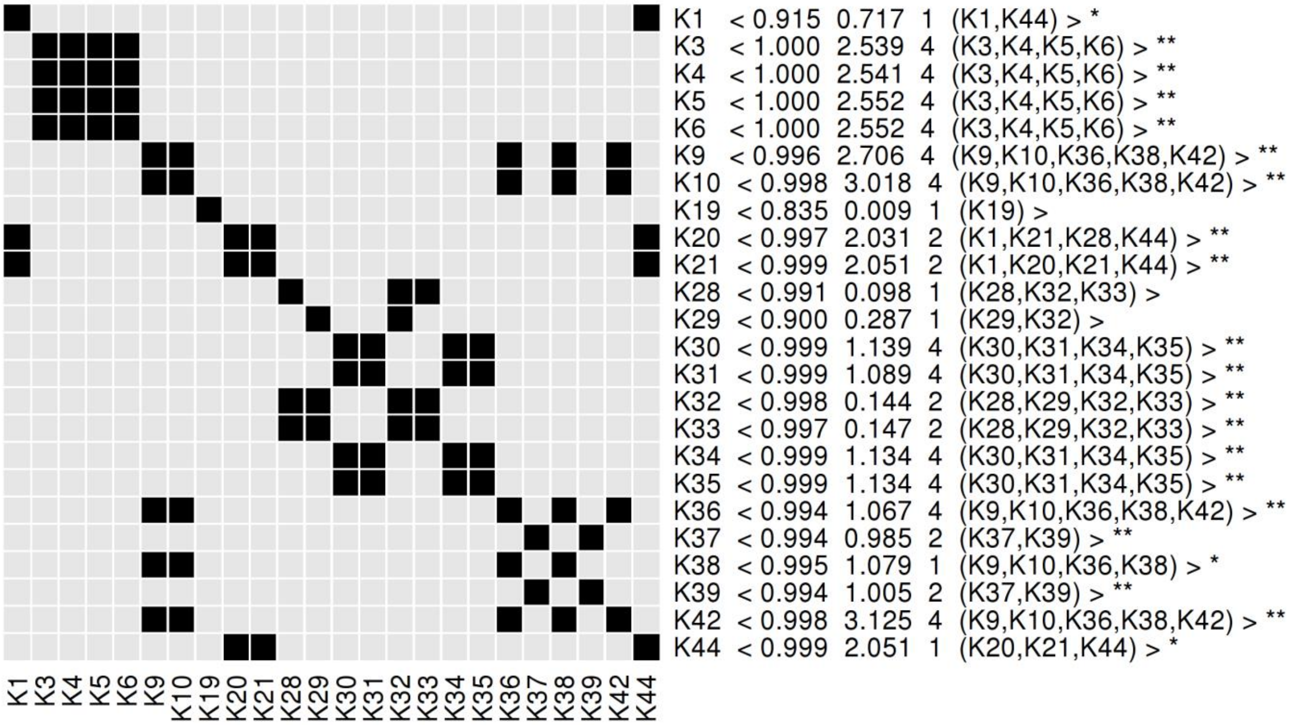

Supplementary Figure 6. MOTA identifiability matrix of the 2nd calibration round for the complete model

In this round 3 parameters were fixed as reported in **Supplementary Table 4**. See **Supplementary Fig. 4** for legend description.

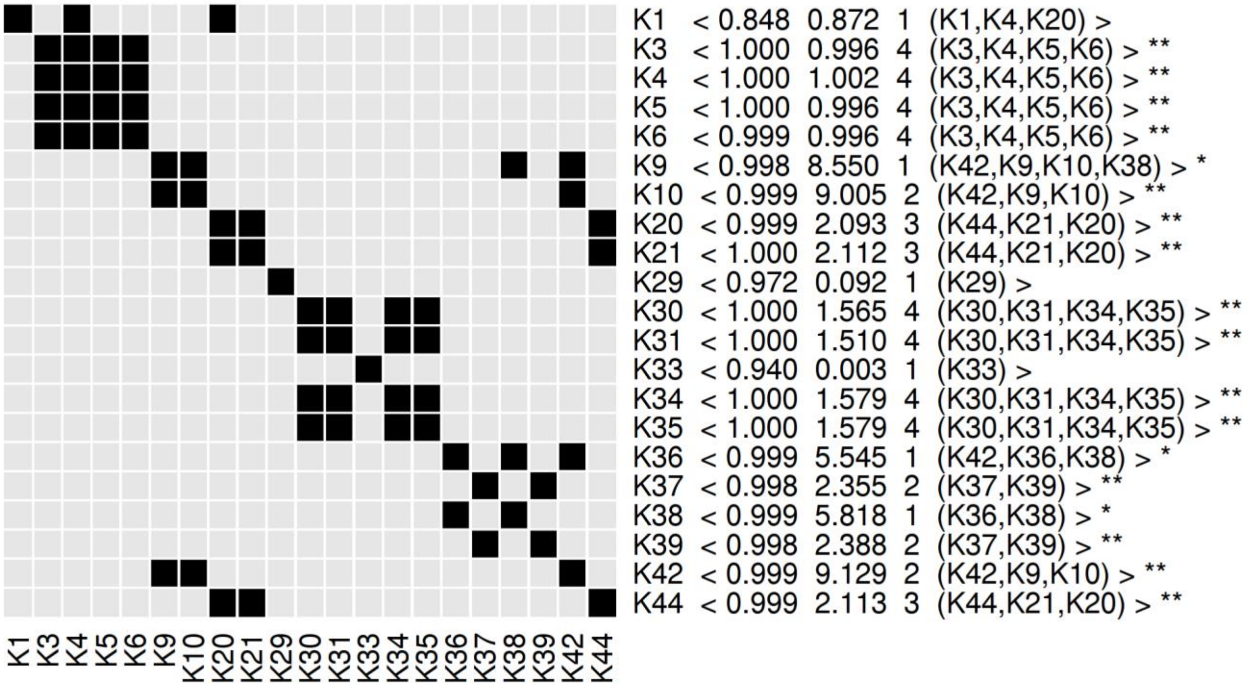

Supplementary Figure 7. MOTA identifiability matrix of the 3rd calibration round for the complete model

In this round 3 parameters were fixed as reported in **Supplementary Table 4**. See **Supplementary Fig. 4** for legend description.

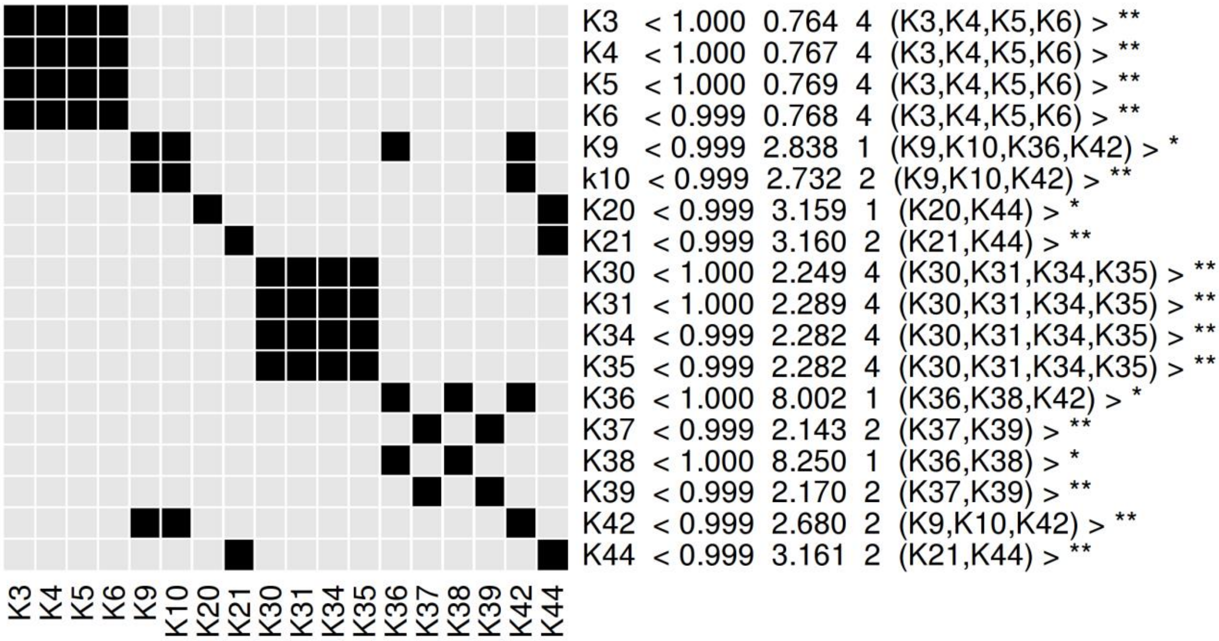

Supplementary Figure 8. MOTA identifiability matrix of the 4th calibration round for the complete model
In this round 1 parameter (K3) were locked as reported in **Supplementary Table 4**. This parameter was part of a quadruplette with K4, K5, K6 which only related to each other and therefore formed a locally defined correlation. See **Supplementary Fig. 4** for legend description.

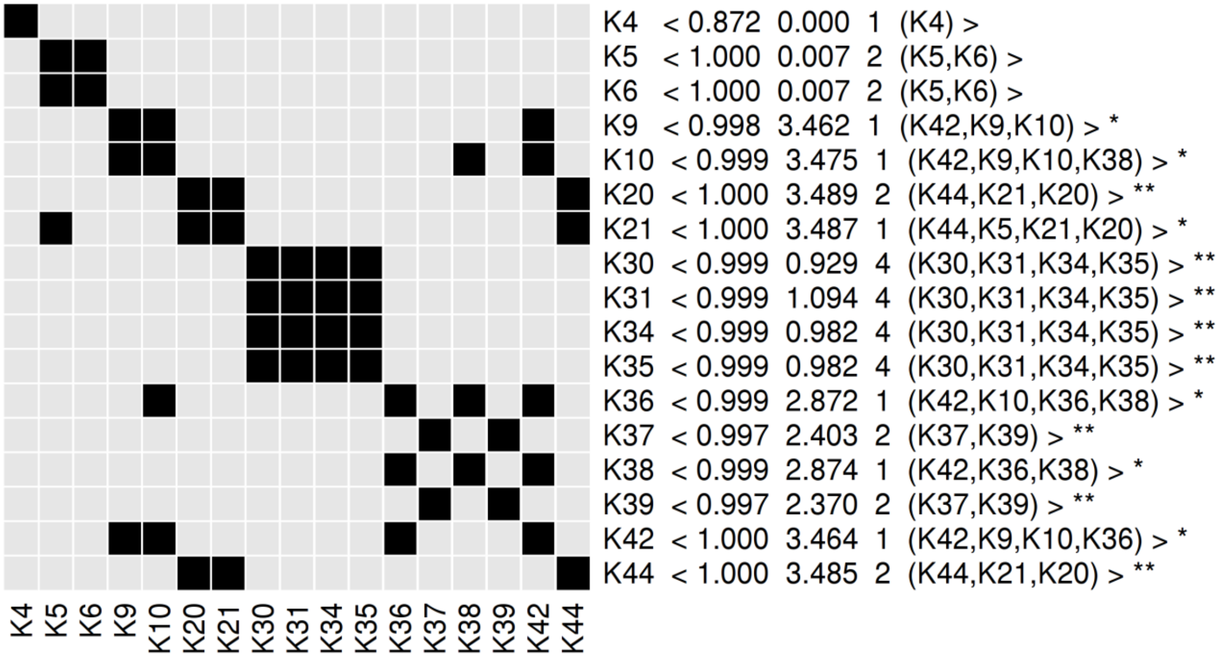

Supplementary Figure 9. MOTA identifiability matrix of the 5th calibration round for the complete model

In this round 3 parameters were fixed and 1 (K30) was locked as reported in **Supplementary Table 4**. This parameter was part of a quadruplette with K31, K34, K35 which only related to each other and therefore formed a locally defined correlation. See **Supplementary Fig. 4** for legend description.

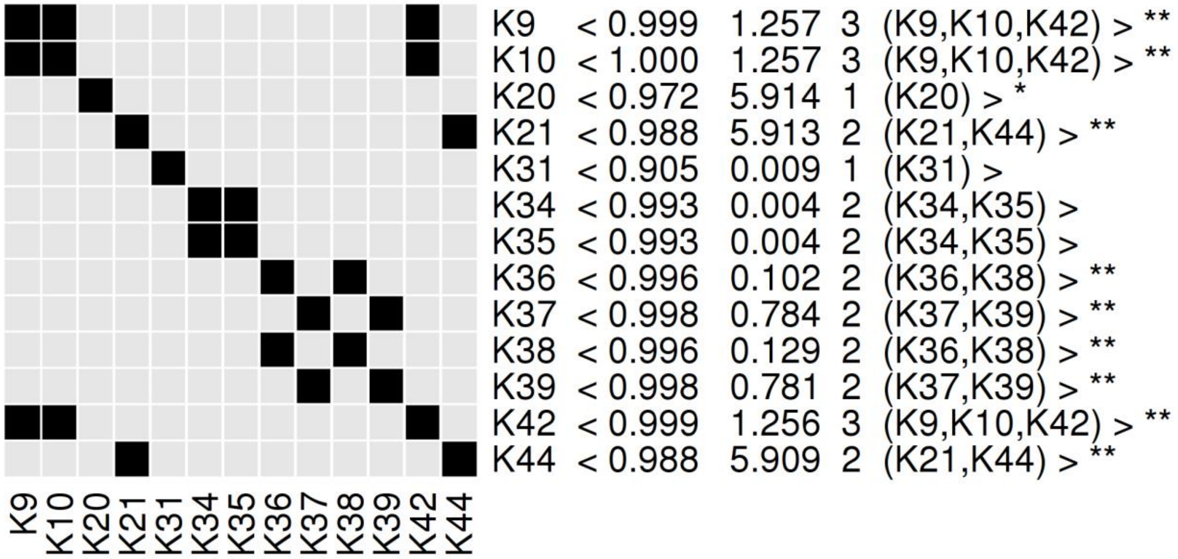

Supplementary Figure 10. MOTA identifiability matrix of the 6th calibration round for the complete model

In this round 3 parameters were fixed and 2 (K38, K39) were locked as reported in **Supplementary Table 4**. These parameters were part of two couples of related parameters. The former K38 with K36, the latter K39 with K37. All these two couples only related internally with each other and therefore formed two locally defined correlations. See **Supplementary Fig. 4** for legend description.

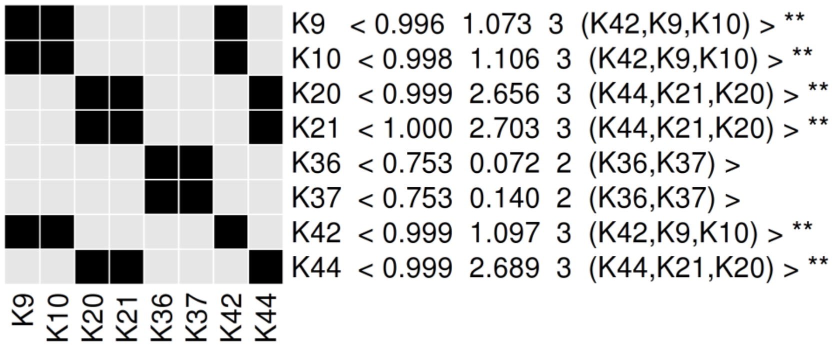

Supplementary Figure 11. MOTA identifiability matrix of the 7th calibration round for the complete model

In this round 2 parameters were fixed and 2 (K10, K21) were locked as reported in **Supplementary Table 4**. These parameters were part of two triplettes of related parameters. The former K10 with K42 and K9, the latter K21 with K44 and K20. All these two couples only related internally with each other and therefore formed two locally defined correlations. See **Supplementary Fig. 4** for legend description.

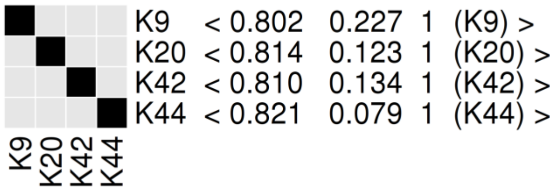

Supplementary Figure 12. MOTA identifiability matrix of the 8th calibration round for the complete model

In this round the last 4 parameters were fixed as reported in **Supplementary Table 4**. See **Supplementary Fig. 4** for legend description.

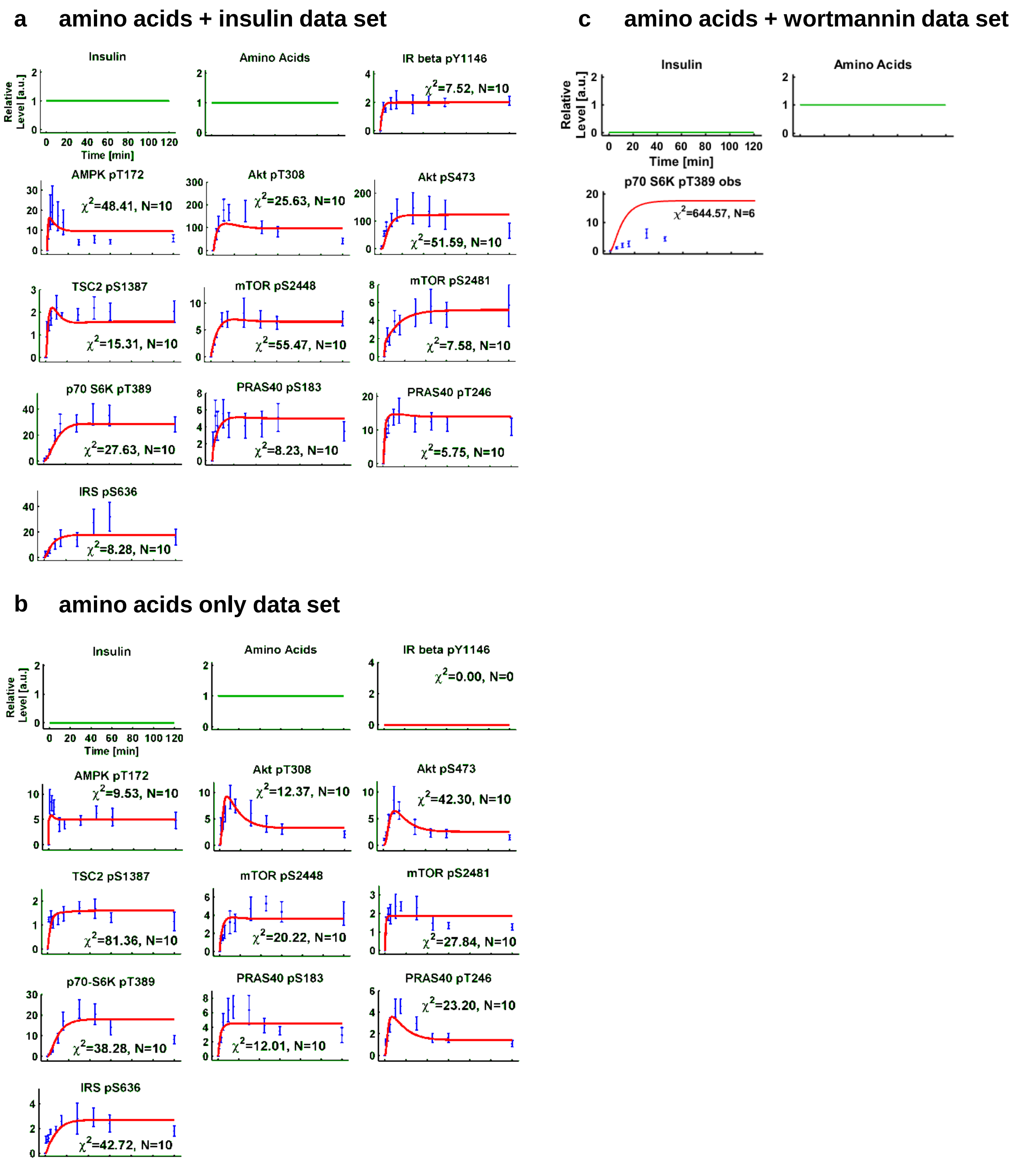

Supplementary Figure 13. Time courses for the model including the four amino acid (aa) inputs Comparison between the simulated time courses (solid lines) and the experimental time courses (points and dotted error bars represent mean and SEM) within [0, 120] min of aa and insulin. This new model including four aa inputs correctly fits the experimental calibration data under the condition of aa + ins (a) and aa only (b).

Also shown is a comparison between simulated and experimentally determined dynamics of p70-S6K-pT389 upon aa stimulation with wortmannin treatment (c). This representation is equivalent to p70-S6K-pT389 simulations and experimental data shown in **Figures 1b, c, d, Supplementary Fig. 14**. The model is unable to predict the effect of wortmannin on p70-S6K-pT389 dynamics.

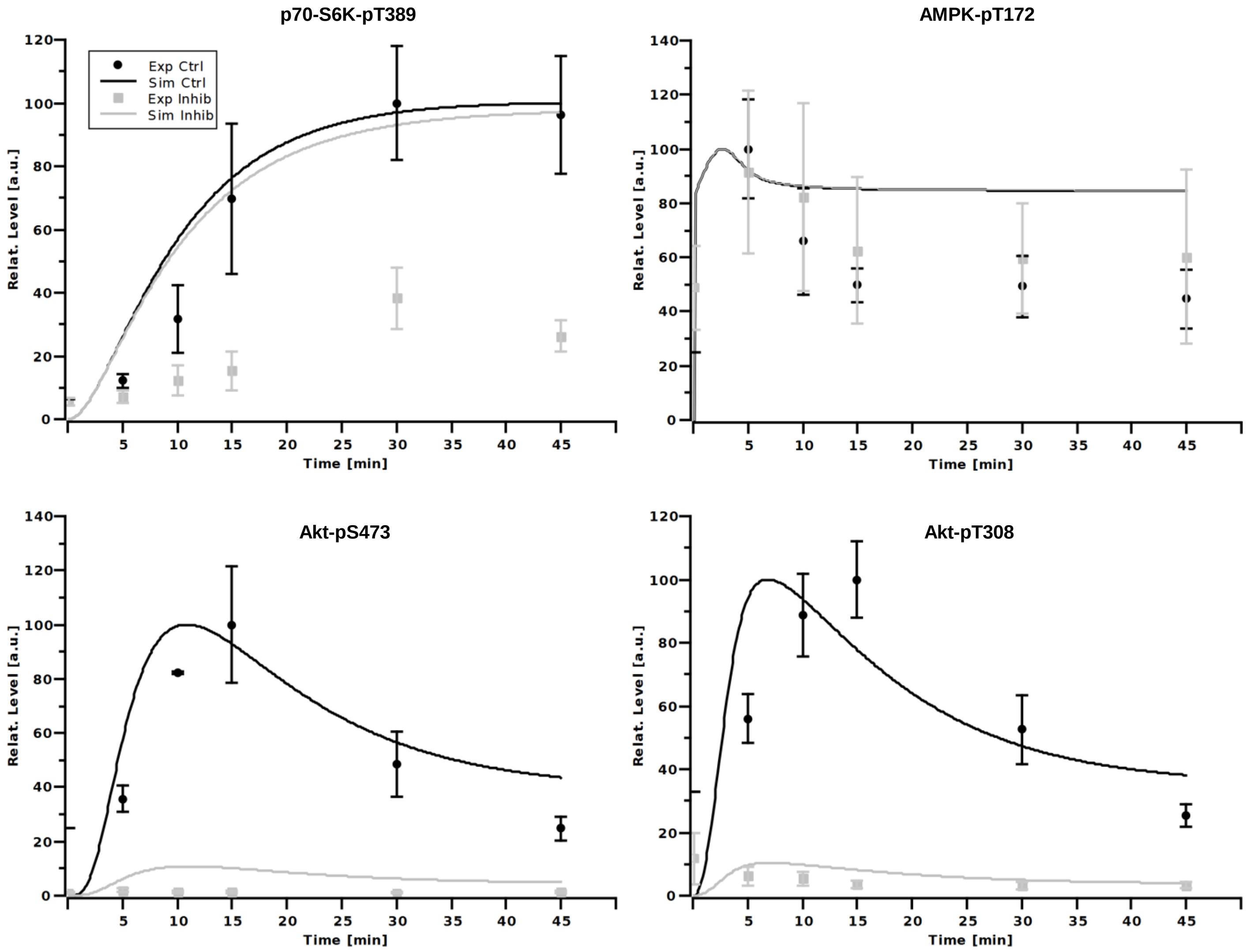

Supplementary Figure 14. Scatter and line graph representations of PI3K inhibition

Equivalent to **Figure 1d**; quantitative representations of simulated (lines) and experimentally (dots) determined dynamics of p70-S6K-pT389, AMPK-pT172, Akt-pS473, and Akt-pT308 upon stimulation with aa, without (black) or with (grey) PI3K inhibition (wortmannin). Shown for the experimental data are the average (dots) and SEM (error bars), N=3. Abbreviations: Exp Ctrl, experimental control condition (DMSO); Exp Inhib, experimental PI3K inhibition (wortmannin); Sim Ctrl, simulated control condition; Sim Inhib, simulated PI3K perturbation.

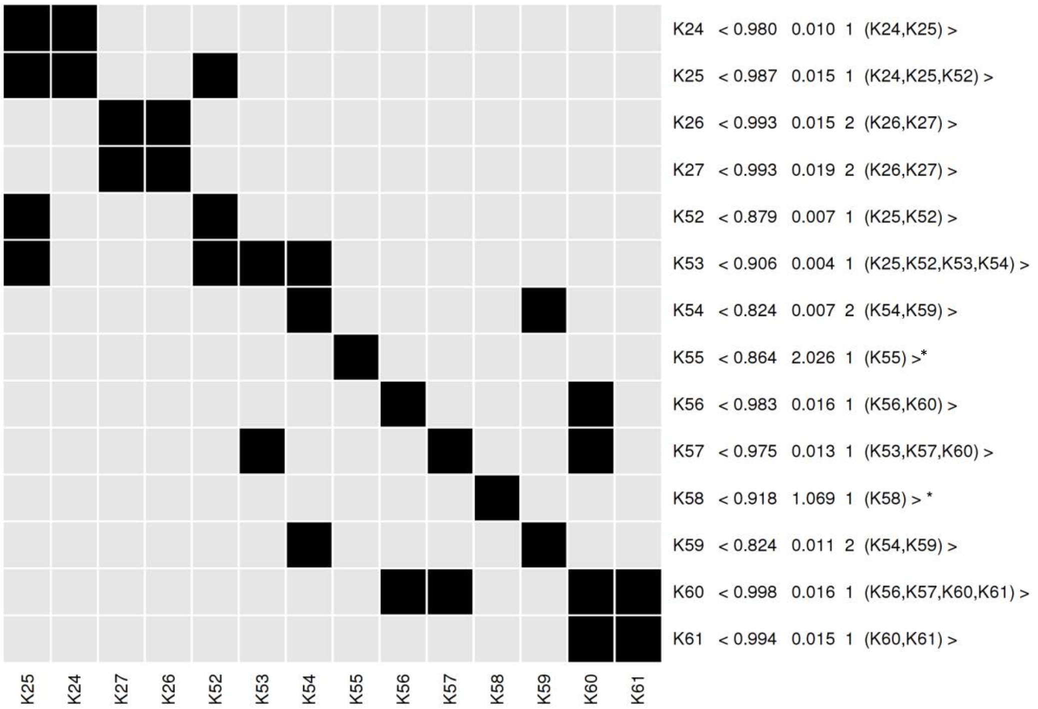

Supplementary Figure 15. MOTA identifiability matrix of the 1st calibration round for the complete model including the new p70-S6K module

In this round the last 12 parameters were fixed as reported in **Supplementary Table 7**. The remaining parameters (K55, K58) were re-estimated in a second round. See **Supplementary Fig. 4** for legend description.

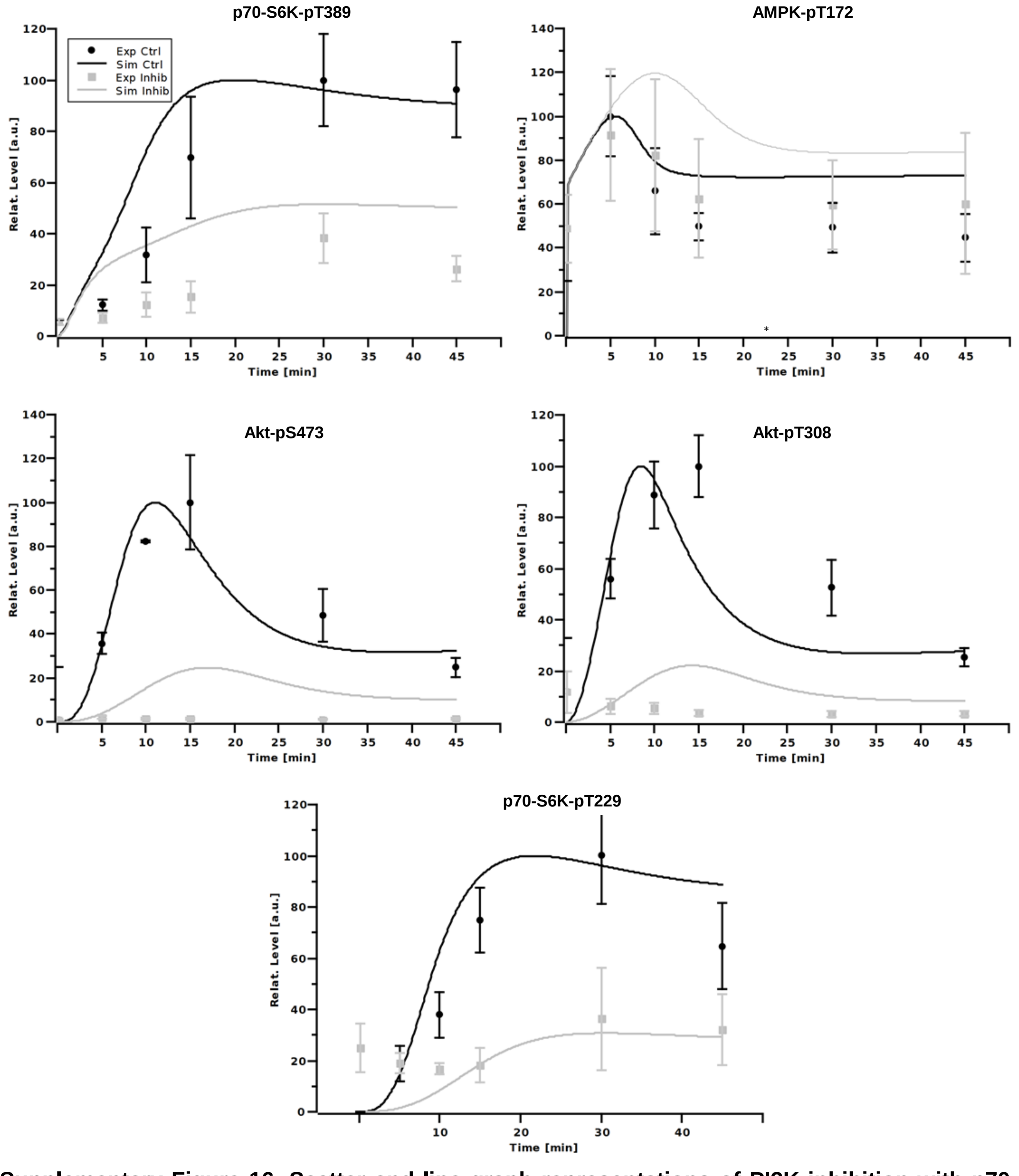

Supplementary Figure 16. Scatter and line graph representations of PI3K inhibition with p70-S6K-pT229 activation

Equivalent to **Figure 3b**; quantitative representations as shown in **Figure 1d** and **S14**, but using the model including the new p70-S6K extended module. Shown are simulated (lines) and experimentally (dots) determined dynamics of p70-S6K-pT389, AMPK-pT172, Akt-pS473, Akt-pT308, and p70-S6K-pT229 upon stimulation with aa, without (black) or with (grey) PI3K inhibition (wortmannin). Simulated quantifications are based on data from **Figure 3a**. Experimental data is the same as shown in **Figure 1c, d** and reported here to compare it with the new model. Shown for the experimental data are the average (dots) and SEM (error bars), N=3. Abbreviations: Exp Ctrl, experimental control condition (DMSO); Exp Inhib, experimental PI3K inhibition (wortmannin); Sim Ctrl, simulated control condition; Sim Inhib, simulated PI3K perturbation.

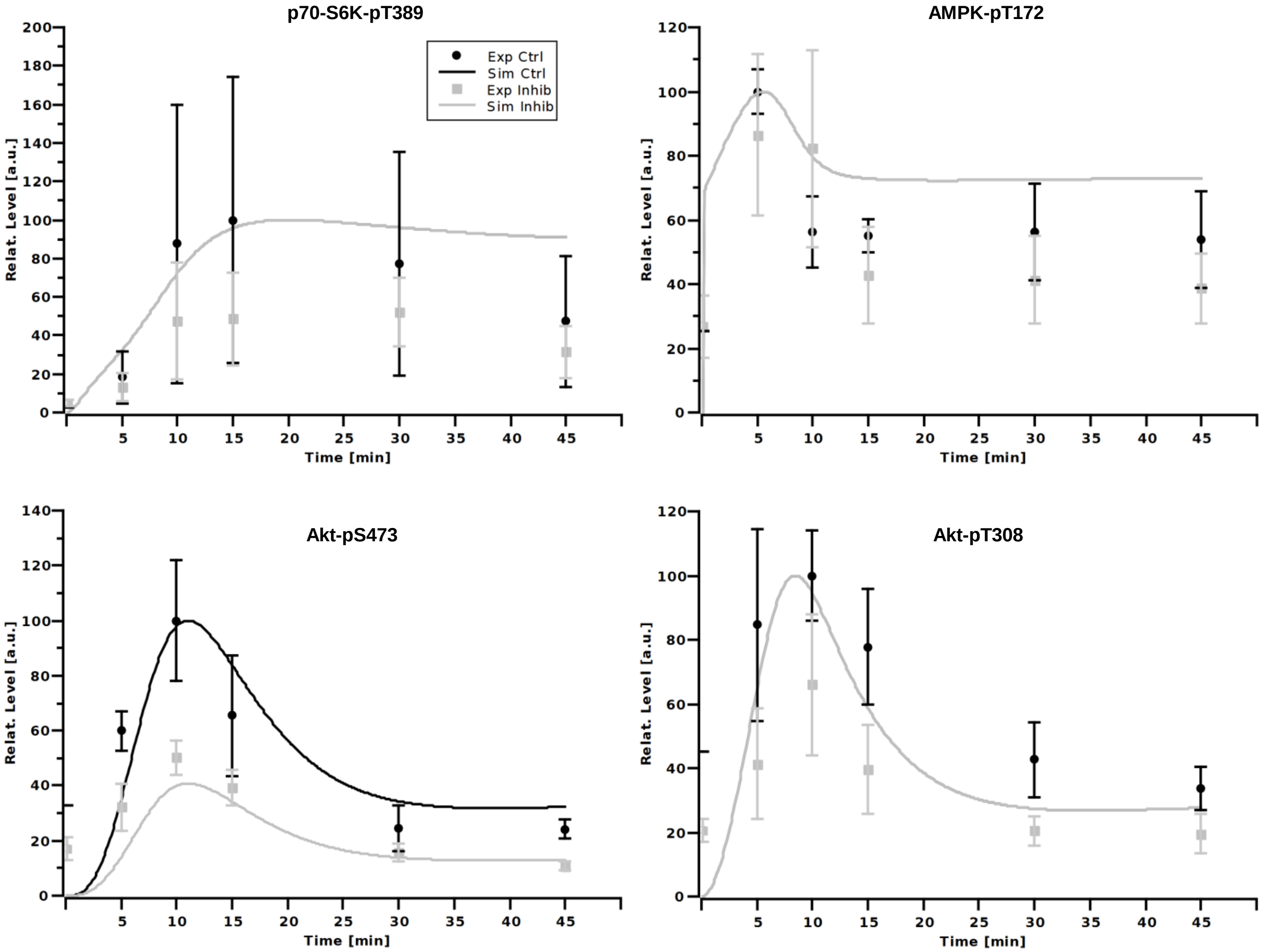

Supplementary Figure 17. Scatter and line graph representations of mTORC2 inhibition
Equivalent to **Figure 4c**; quantitative representations of simulated (lines) and experimentally (dots) determined dynamics of p70-S6K-pT389, AMPK-pT172, Akt-pS473, and Akt-pT308 upon stimulation with aa without (black) or with (grey) mTORC2 inhibition using shSin1. Shown for the experimental data are the average (dots) and SEM (error bars), N=3. Abbreviations: Exp Ctrl, experimental control condition (non-silencing); Exp Inhib, experimental mTORC2 inhibition (shSin1); Sim Ctrl, simulated control condition; Sim Inhib, simulated mTORC2 perturbation.

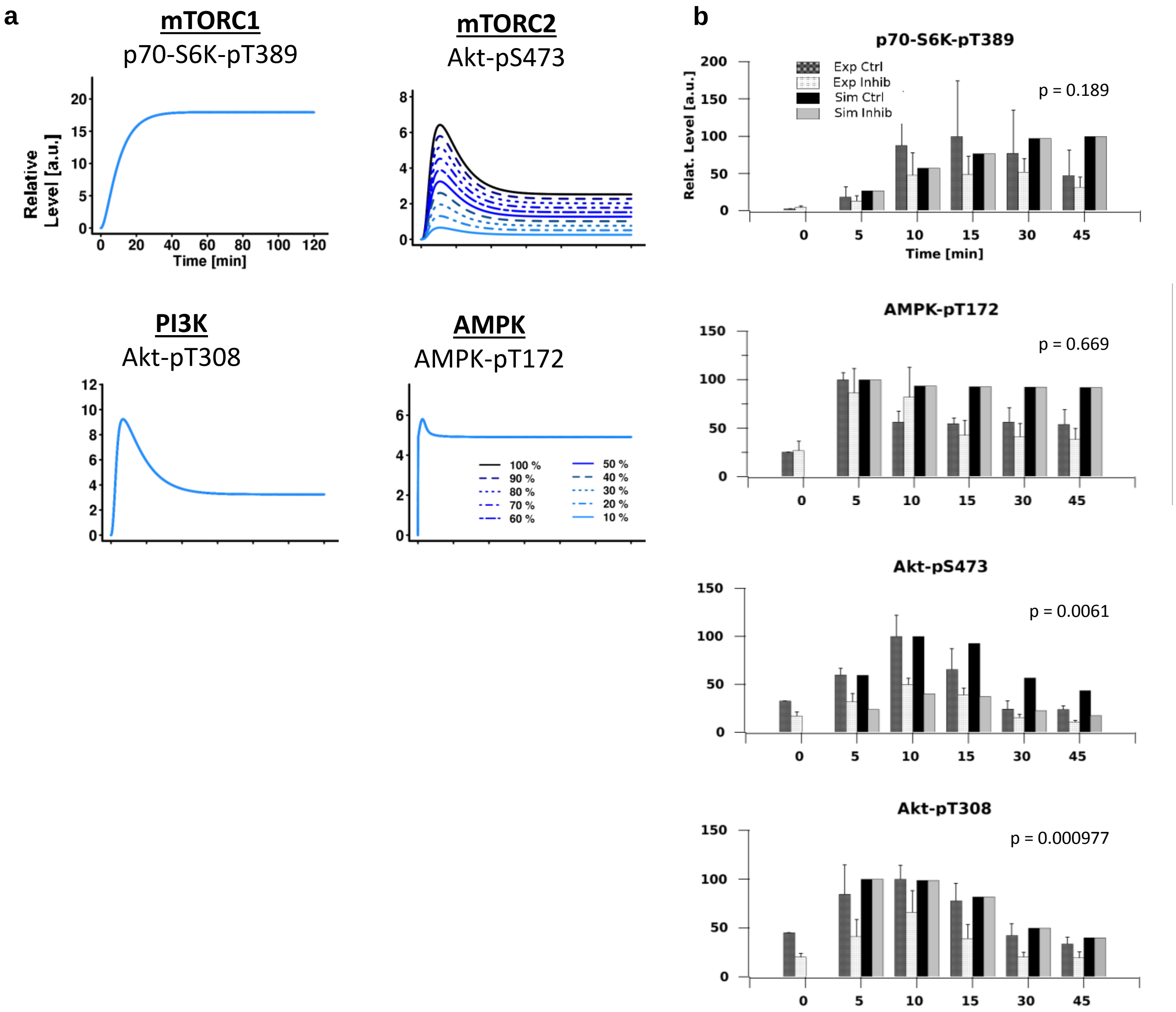

Supplementary Figure 18. mSin1 inhibition data comparison using the simple p70-S6K module (corresponding to Figure 4)

Simulated perturbation of mTORC2 using the model with a simplified p70-S6K module (**Supplementary Table 4**, **Supplementary Fig. 13**), and comparison with the experimental quantification data. The prediction is consistent with **Figure 4**, although slightly less accurate.

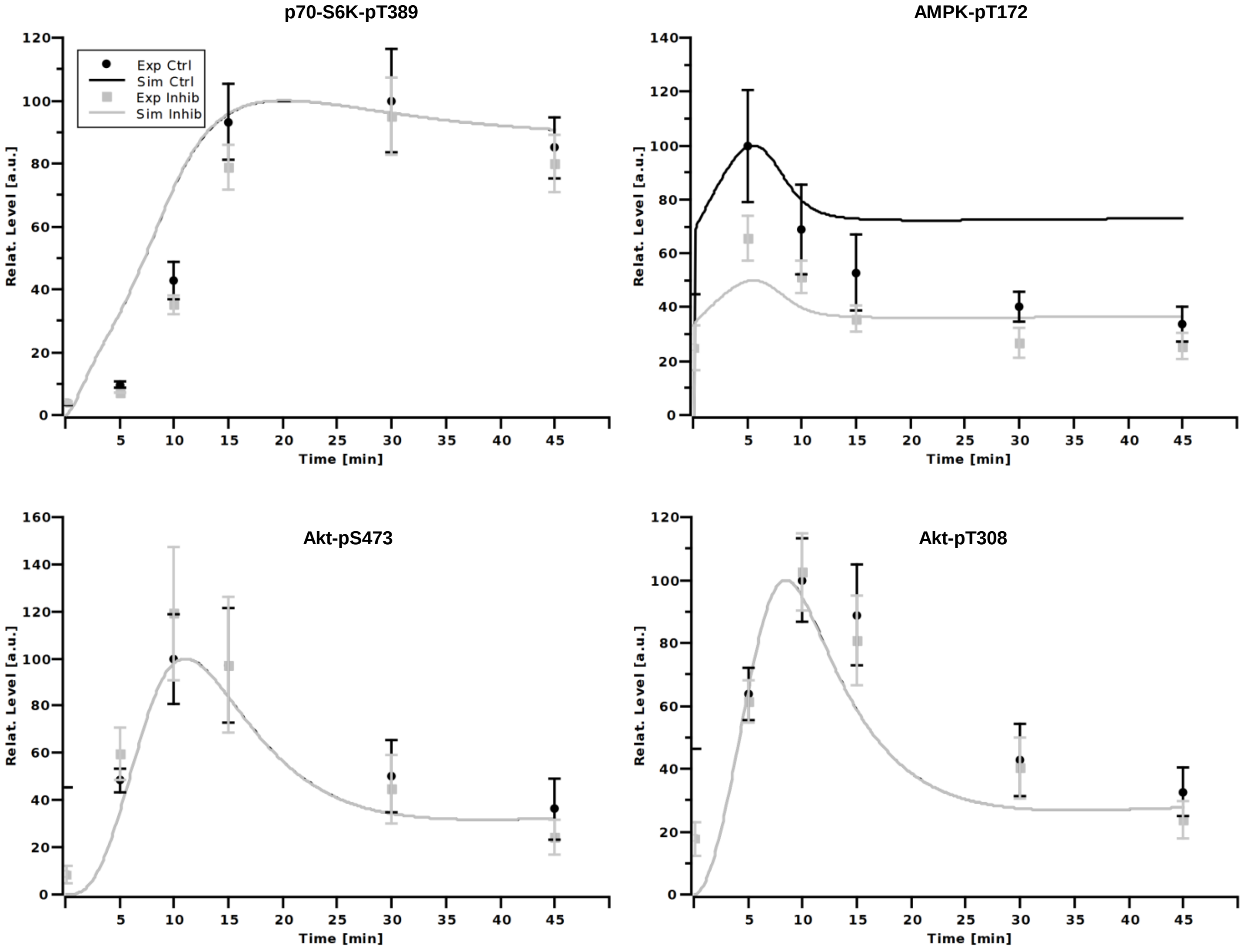

Supplementary Figure 19. Scatter and line graph representations of AMPK inhibition
Equivalent to **Figure 5e**; quantitative representations of simulated (lines) and experimentally (dots) determined dynamics of p70-S6K-pT389, AMPK-pT172, Akt pS473, and Akt pT308 upon stimulation with aa without (black) or with (grey) CamKKβ inhibition by STO-609. Shown for the experimental data are the average (dots) and SEM (error bars), N=4. Abbreviations: Exp Ctrl, experimental control condition (DMSO); Exp Inhib, experimental AMPK perturbation by CaMKKβ inhibition (STO-609); Sim Ctrl, simulated control condition; Sim Inhib, simulated AMPK perturbation.

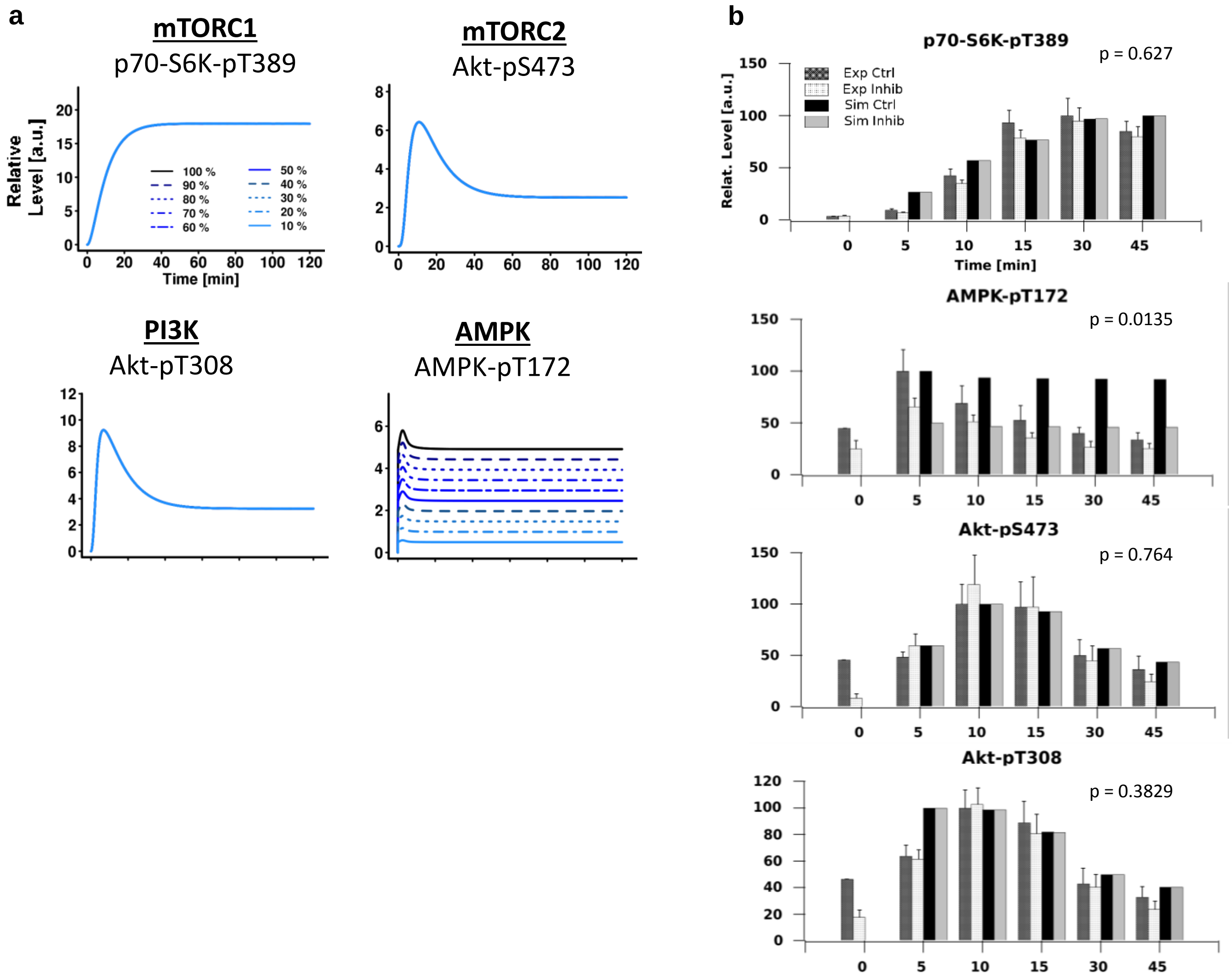

Supplementary Figure 20. AMPK inhibition data comparison using the simple p70-S6K module (corresponding to Figure 5)

Simulated perturbation of AMPK using the model with a simplified p70-S6K module (**Supplementary Table 4**, **Supplementary Fig. 13**), and comparison with the experimental quantification data. The prediction is consistent with **Figure 5**, although slightly less accurate.

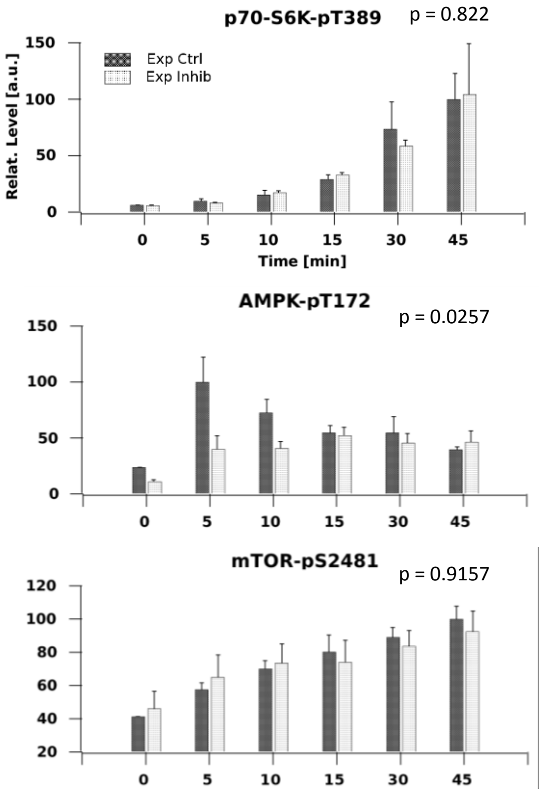

Supplementary Figure 21. Amino acids activate mTORC1, mTORC2, and PI3K independently of AMPK (related to Figure 5f)

Quantitative representations of experimentally determined dynamics of p70-S6K-pT389, AMPK-pT172, and mTOR-pS2481 dynamics upon siAMPKα1+2 and induction with aa. Data are the SEM, N=3. Abbreviations: Exp Ctrl, experimental control condition (siCtrl); Exp Inhib, experimental AMPK inhibition (siAMPKα1+2).

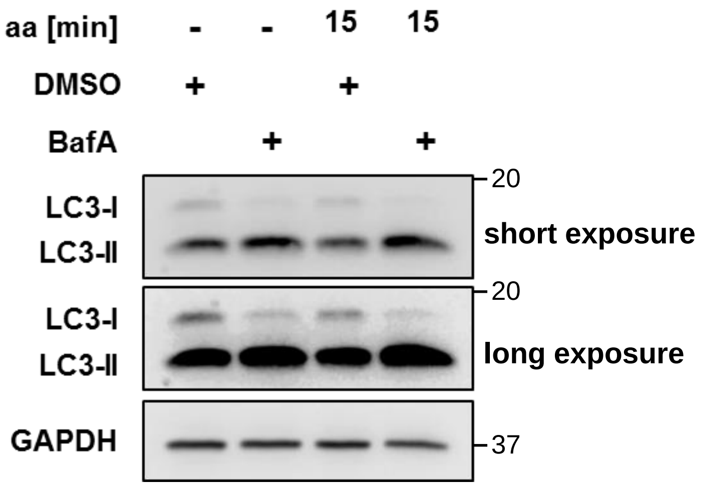

Supplementary Figure 22. Bafilomycin A1 (BafA) enhances LC3-II accumulation in starved and amino acids (aa) stimulated cells

Bafilomycin A1 (BafA) enhances LC3-II accumulation in starved and aa-stimulated cells. Shown are immunoblot results of aa-stimulated and aa-starved C2C12 cells in the presence or absence of BafA. Data are representative of 3 experiments.

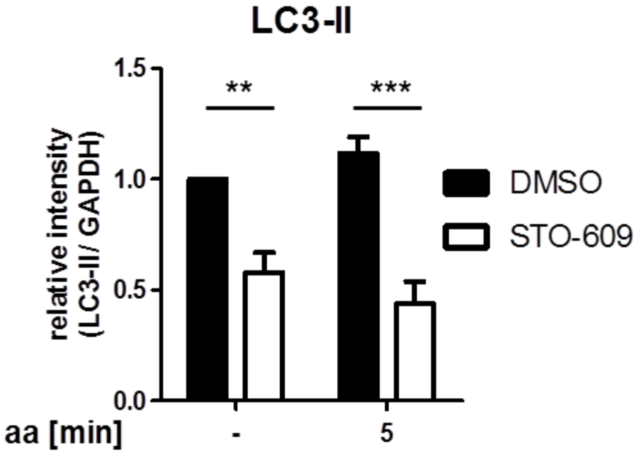

Supplementary Figure 23. The CaMKKβ inhibitor STO-609 reduces levels of the autophagy marker LC3-II in amino acids (aa)-stimulated cells (related to Figure 5j)

Quantitative representations of LC3-II levels upon aa-stimulation in presence or absence of the CaMKKβ inhibitor STO-609. Data were normalized to 1 for the aa-starvation/ DMSO condition and represented as mean and SEM, N=3 . A repeated measurement two-way ANOVA followed by Bonferroni's multiple comparison test was applied. **, p<0,01; ***, p<0,001

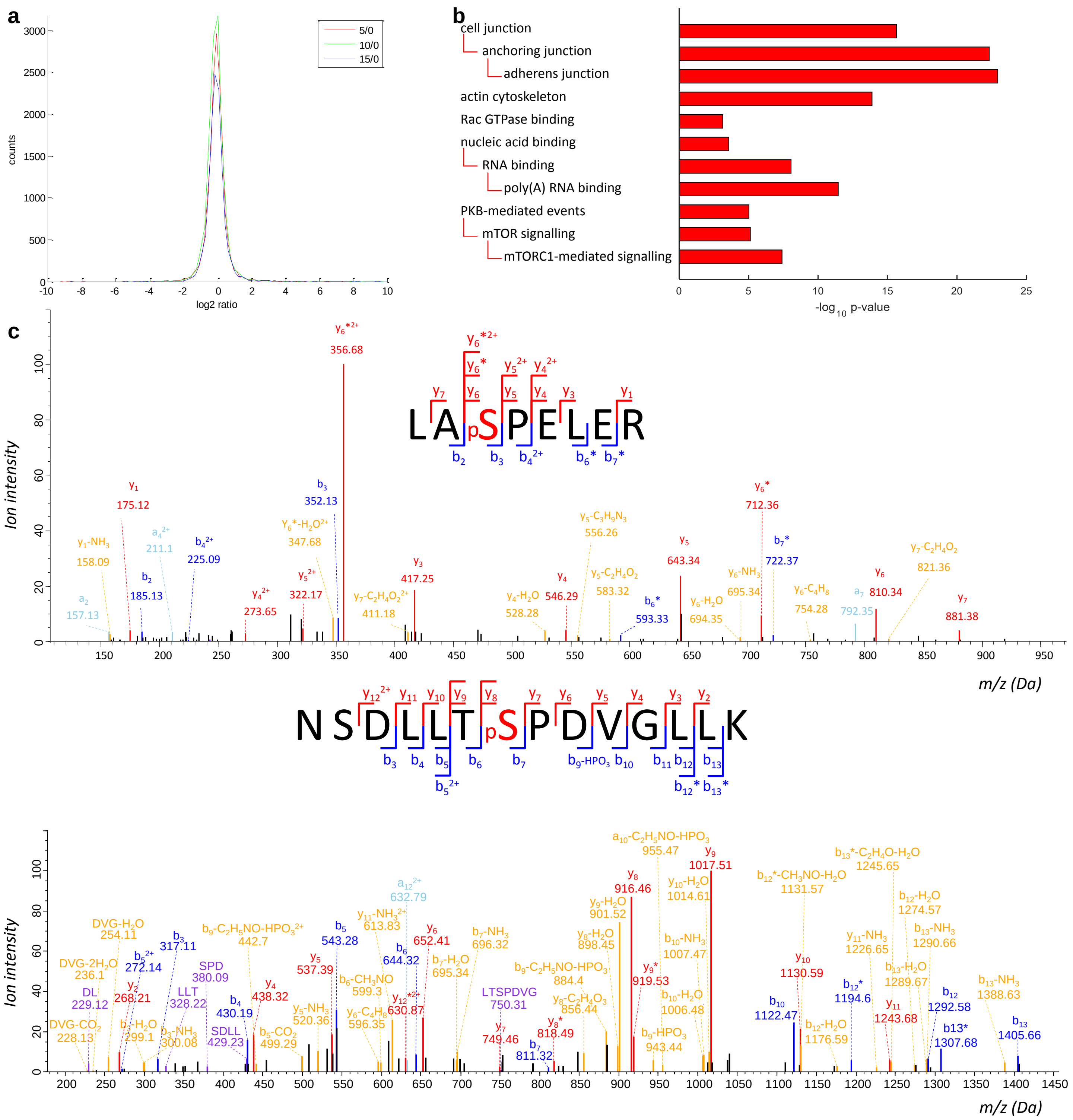

Supplementary Figure 24. Quantitative SILAC-based phosphoproteome analysis of C2C12 cells under aa stimulation

(a) Distribution of SILAC ratios for 5, 10 and 15 minutes versus 0 minutes aa-readdition. The high symmetry and similarity of the SILAC ratio distributions for the different time points reflect the high quality of quantification data. 5/0, ratio 5 versus 0 minutes; 10/0, ratio 10 versus 0 minutes; 15/0, ratio 15 versus 0 minutes based on “Phospho (STY)Sites.txt”.

(b) GO-term analysis performed with gene lists of differentially regulated phosphosites (**Supplementary Data 1,** 2-fold regulated) using the gProfiler tool[1,2] (c) Annotated MS/MS spectra of c-Jun phosphopeptides containing the sites c-Jun-pS73 (sequence: LASPELER) and c-Jun-p-S63 (sequence: NSDLLTSPDVGLLK) (**Figure 6c**). Ion fragments with * hold phosphoryl group, and red residue with p prefix show the phosphorylation localisation in the peptide sequence.

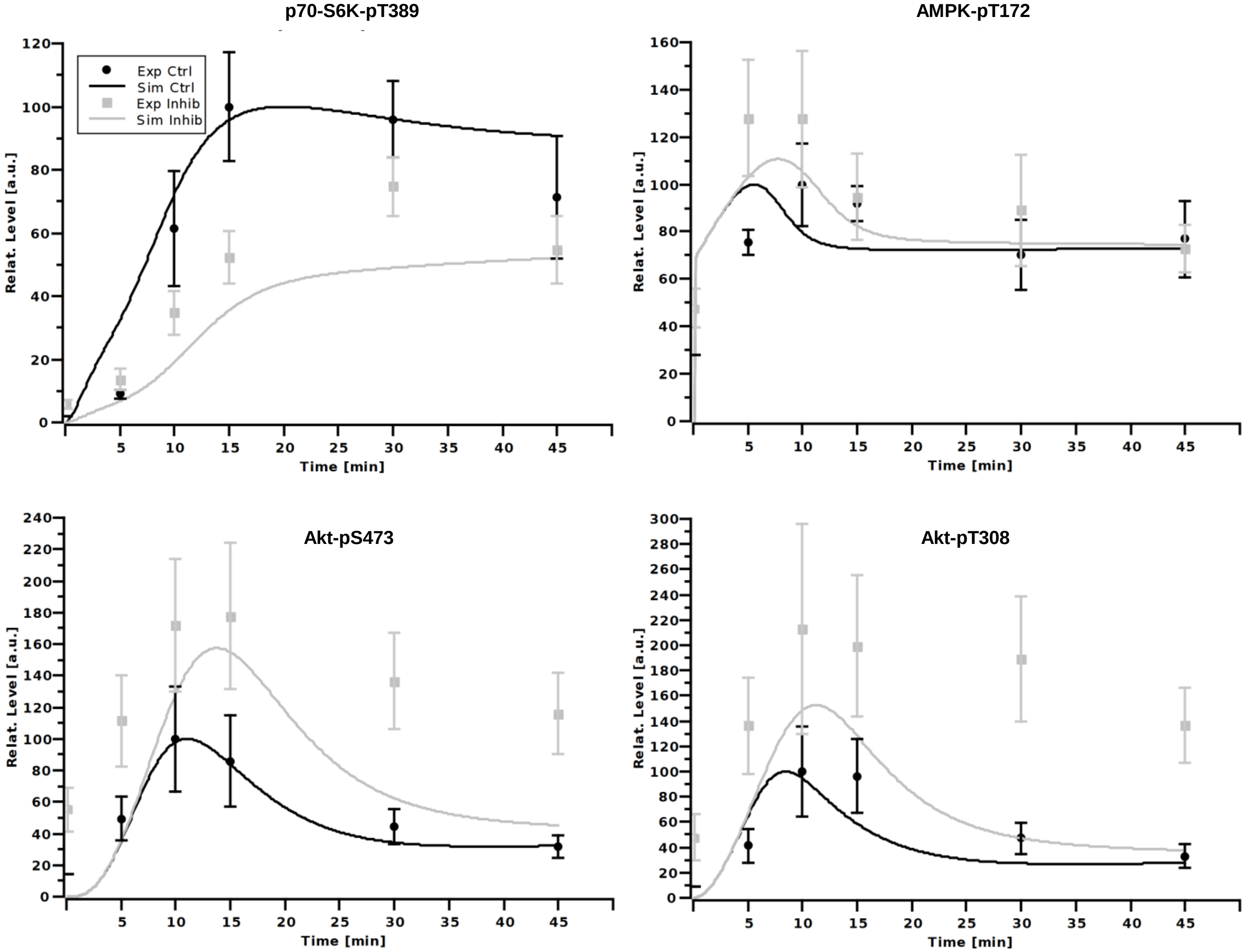

Supplementary Figure 25. Scatter and line graph representations of mTORC1 inhibition
Equivalent to **Figure 7c**; quantitative representations of simulated (lines) and experimentally (dots) determined dynamics of p70-S6K-pT389, AMPK-pT172, Akt pS473, and Akt pT308 upon stimulation with aa without (black) or with (grey) mTORC1 inhibition using shRaptor. Shown for the experimental data are the average (dots) and SEM (error bars), N=4. Abbreviations: Exp Ctrl, experimental control condition (shScramble); Exp Inhib, experimental mTORC1 inhibition (shRaptor); Sim Ctrl, simulated control condition; Sim Inhib, simulated mTORC1 perturbation.

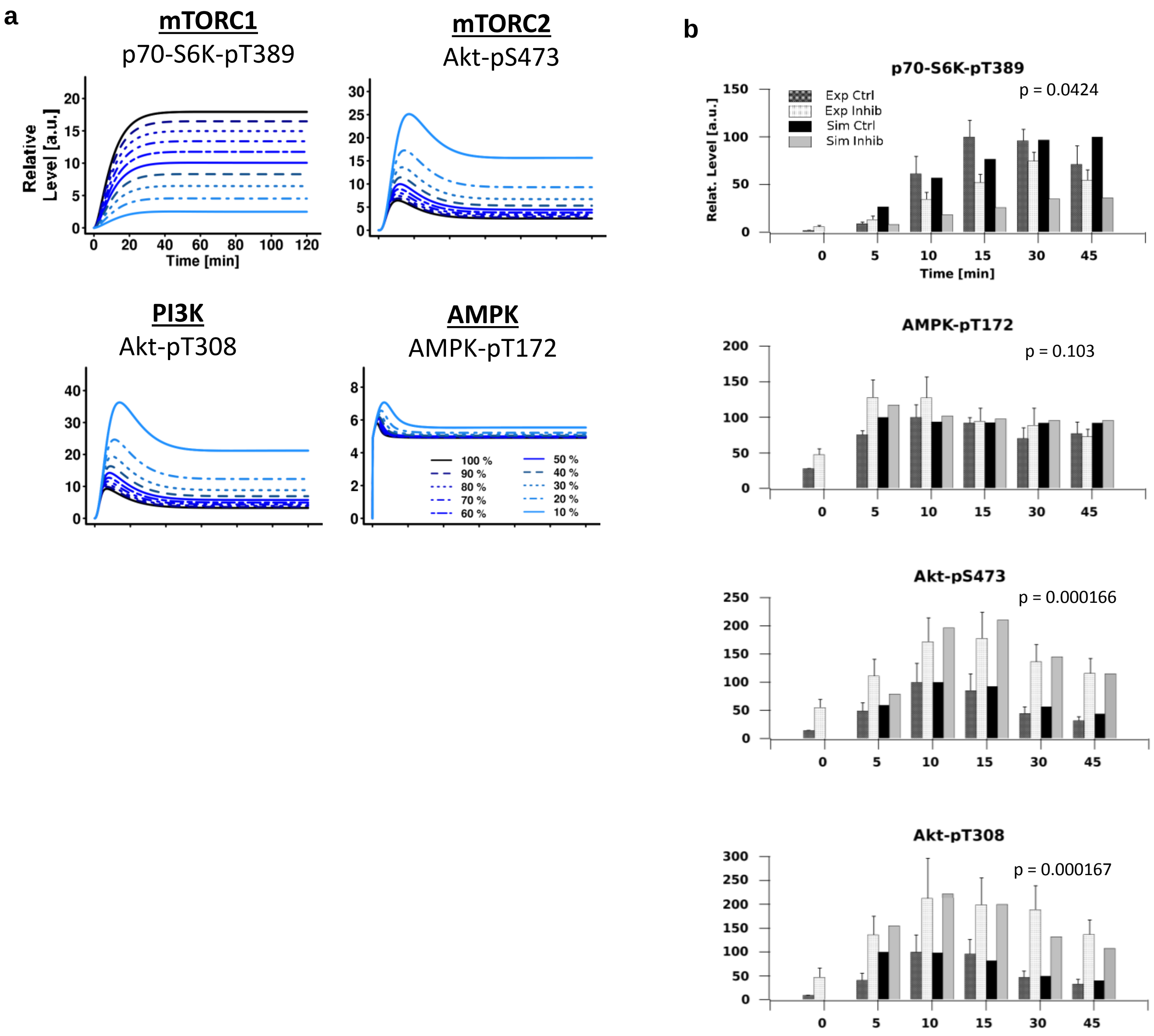

Supplementary Figure 26. Raptor inhibition data comparison using the simple p70-S6K module (corresponding to Figure 7)

Simulated perturbation of mTORC1 using the model with a simplified p70-S6K module (**Supplementary Table 4**, **Supplementary Fig. 13**), and comparison with the experimental quantification data. The prediction is consistent with **Figure 7**, although slightly less accurate.

a

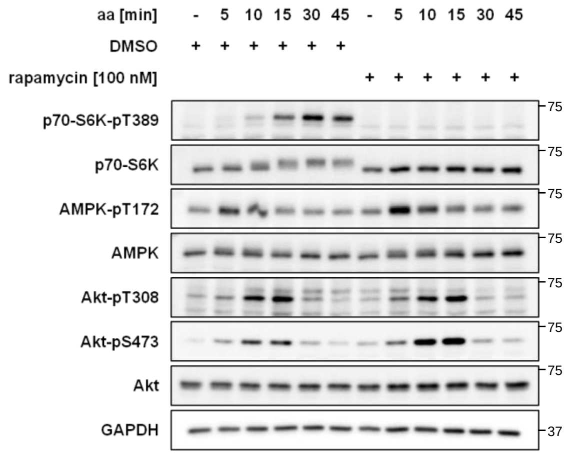

b

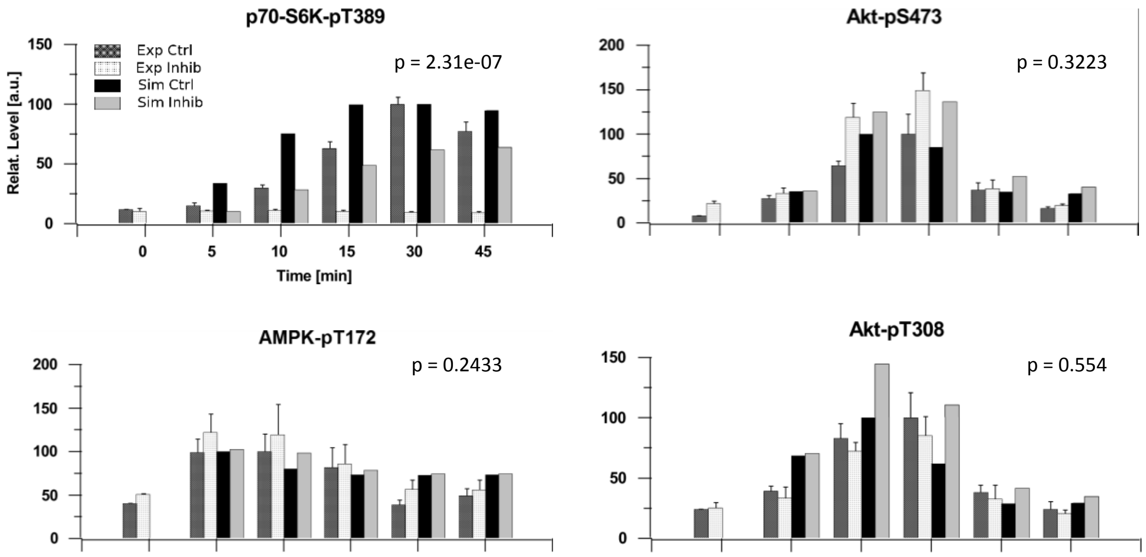

Supplementary Figure 27. Rapamycin does not prevent amino acids (aa)-stimulated increase of AMPK-pT172, Akt-pT308 and Akt-pS473 (related to Figure 7)

(a) Aa enhance AMPK-pT172, Akt-pT308, and Akt-pS473 upon mTORC1 inhibition with rapamycin. Representative immunoblot results of aa-stimulated C2C12 cells in the presence or absence of 100 nM rapamycin. Data are representative of 3 experiments.

(b) Quantitative representations of simulated (mTORC1 inhibition: 25%) and experimentally determined dynamics of p70-S6K-pT389, Akt-pS473, Akt-pT308, and AMPK-pT172 upon stimulation with aa with our without rapamycin. Data are the average and SEM. N=3. Abbreviations: Exp Ctrl, experimental control condition (DMSO); Exp Inhib, experimental mTORC1 inhibition (rapamycin); Sim Ctrl, simulated control condition; Sim Inhib, simulated mTORC1 perturbation.

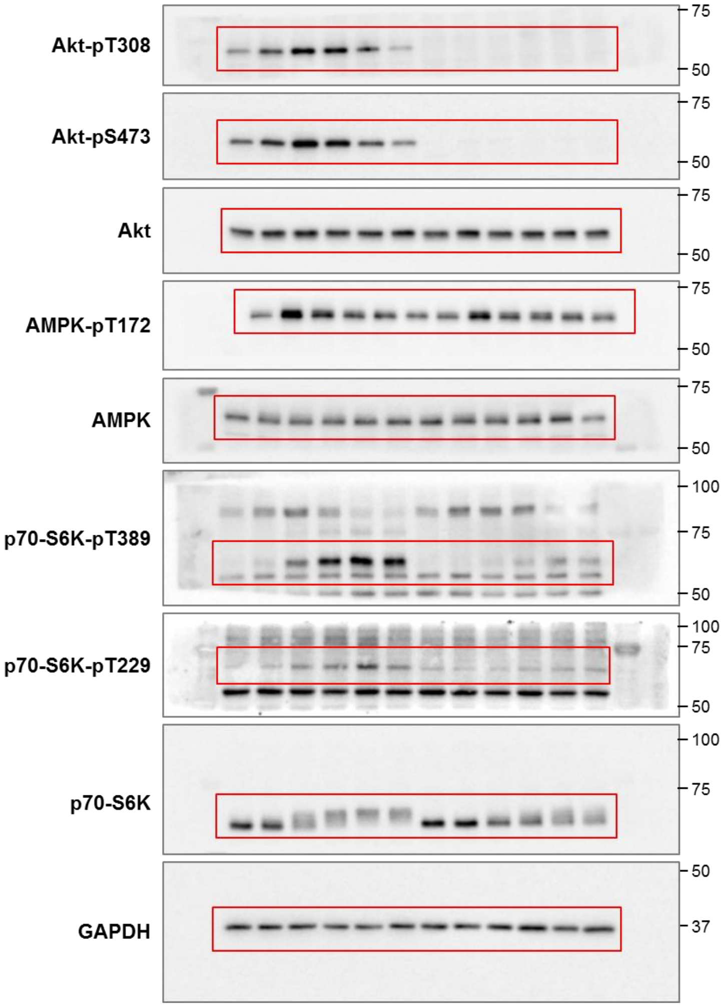

Supplementary Figure 28. Uncropped immunoblots for Figure 1c
Chemiluminiscence detections of the whole membranes for each protein detected are shown. The molecular weight is indicated in kDa on the right side. The red squares indicate the sections shown in **Figure 1c**.

a

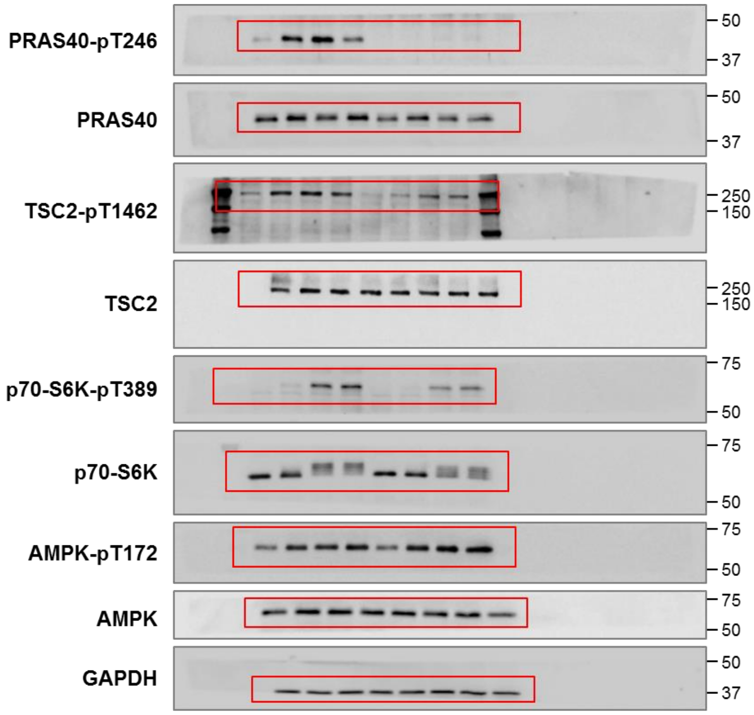

b

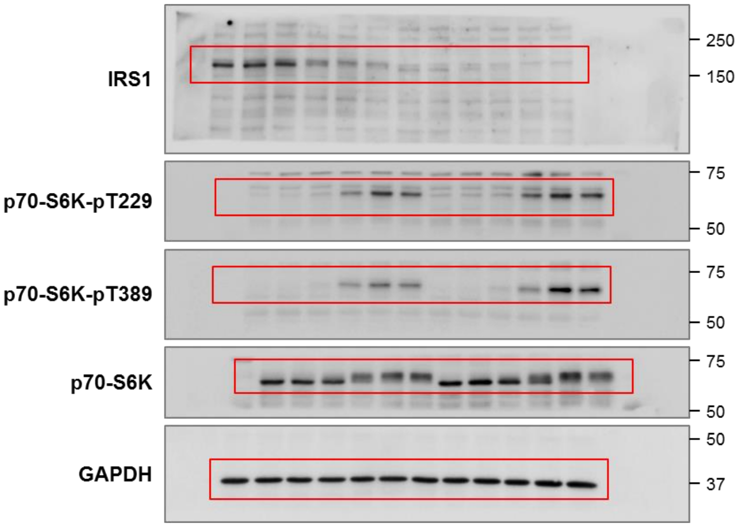

Supplementary Figure 29. Uncropped immunoblots for Figure 3
Chemiluminiscence detections of the whole membranes for each protein detected are shown. The molecular weight is indicated in kDa on the right side. The red squares indicate the sections shown in (a) **Figure 3c**, (b) **Figure 3d**.

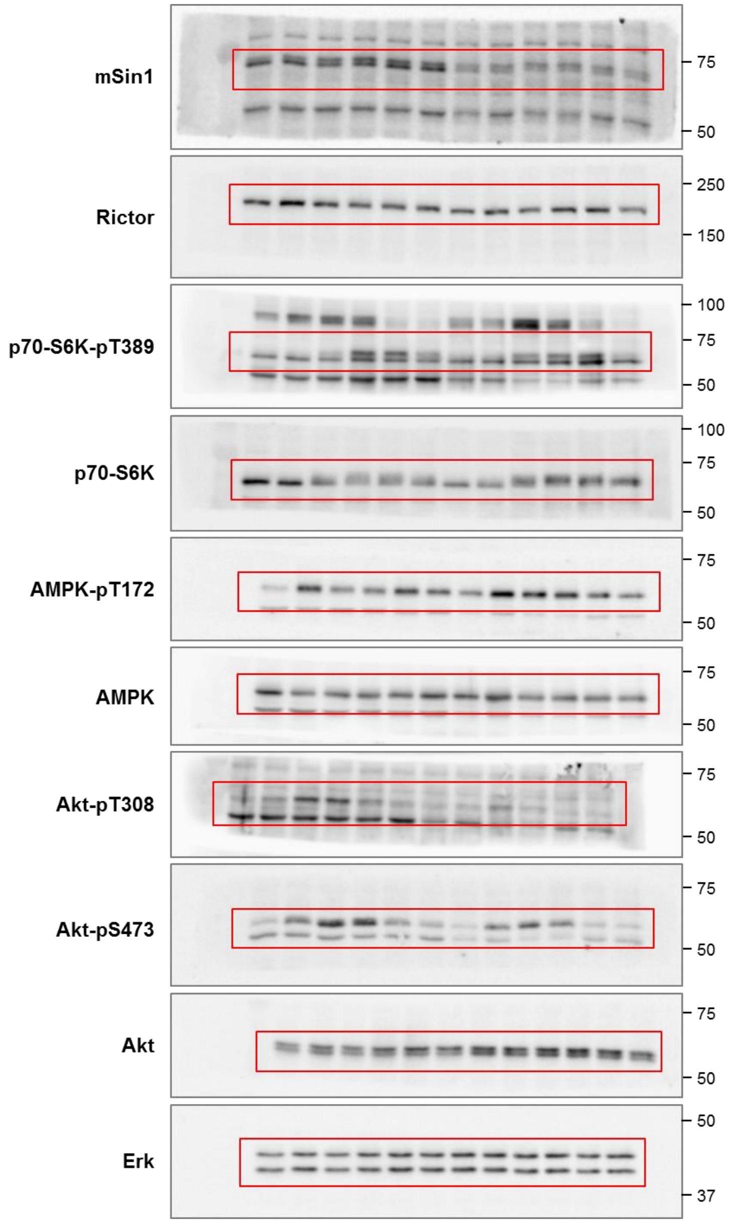

Supplementary Figure 30. Uncropped immunoblots for Figure 4b

Chemiluminiscence detections of the whole membranes for each protein detected are shown. The molecular weight is indicated in kDa on the right side. The red squares indicate the sections shown in **Figure 4b**.

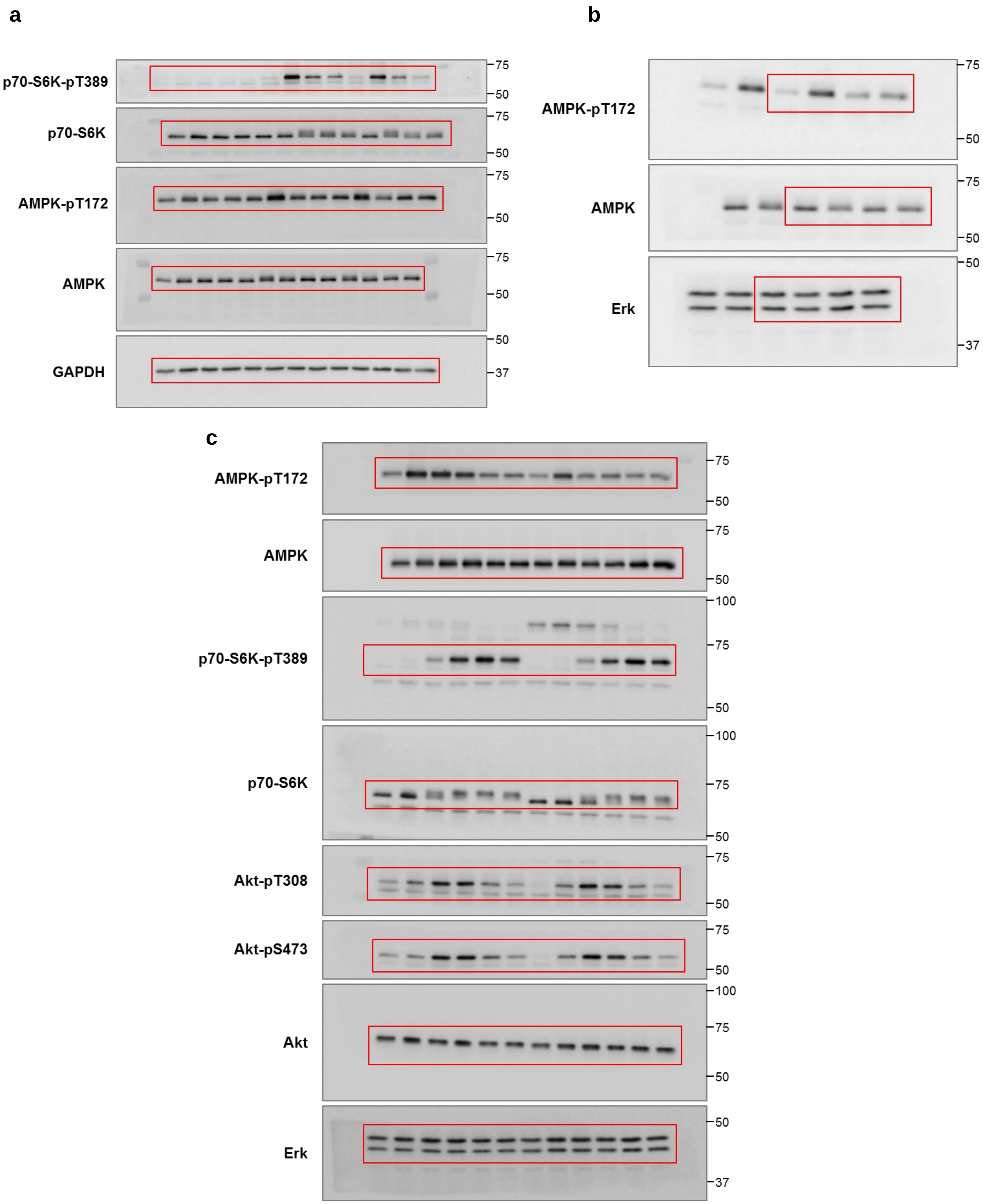

Supplementary Figure 31. Uncropped immunoblots for Figure 5a, 5b, and 5d
Chemiluminiscence detections of the whole membranes for each protein detected are shown. The molecular weight is indicated in kDa on the right side. The red squares indicate the sections shown in (a) **Figure 5a,** (b) **Figure 5b,** (c) **Figure 5d.**

.

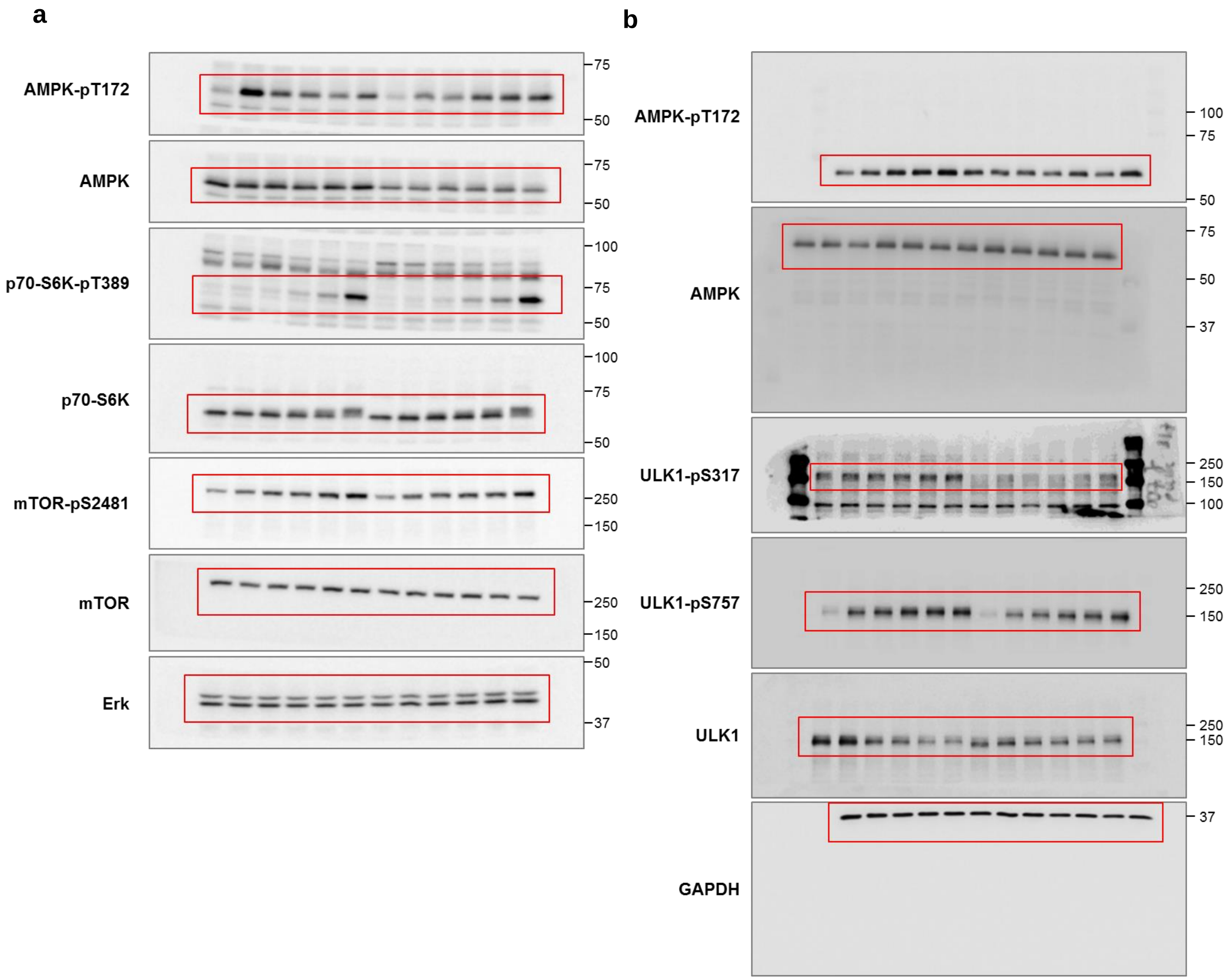

Supplementary Figure 32. Uncropped immunoblots for Figure 5f and 5g
Chemiluminiscence detections of the whole membranes for each protein detected are shown. The molecular weight is indicated in kDa on the right side. The red squares indicate the sections shown in (a) **Figure 5f**, (b) **Figure 5g**.

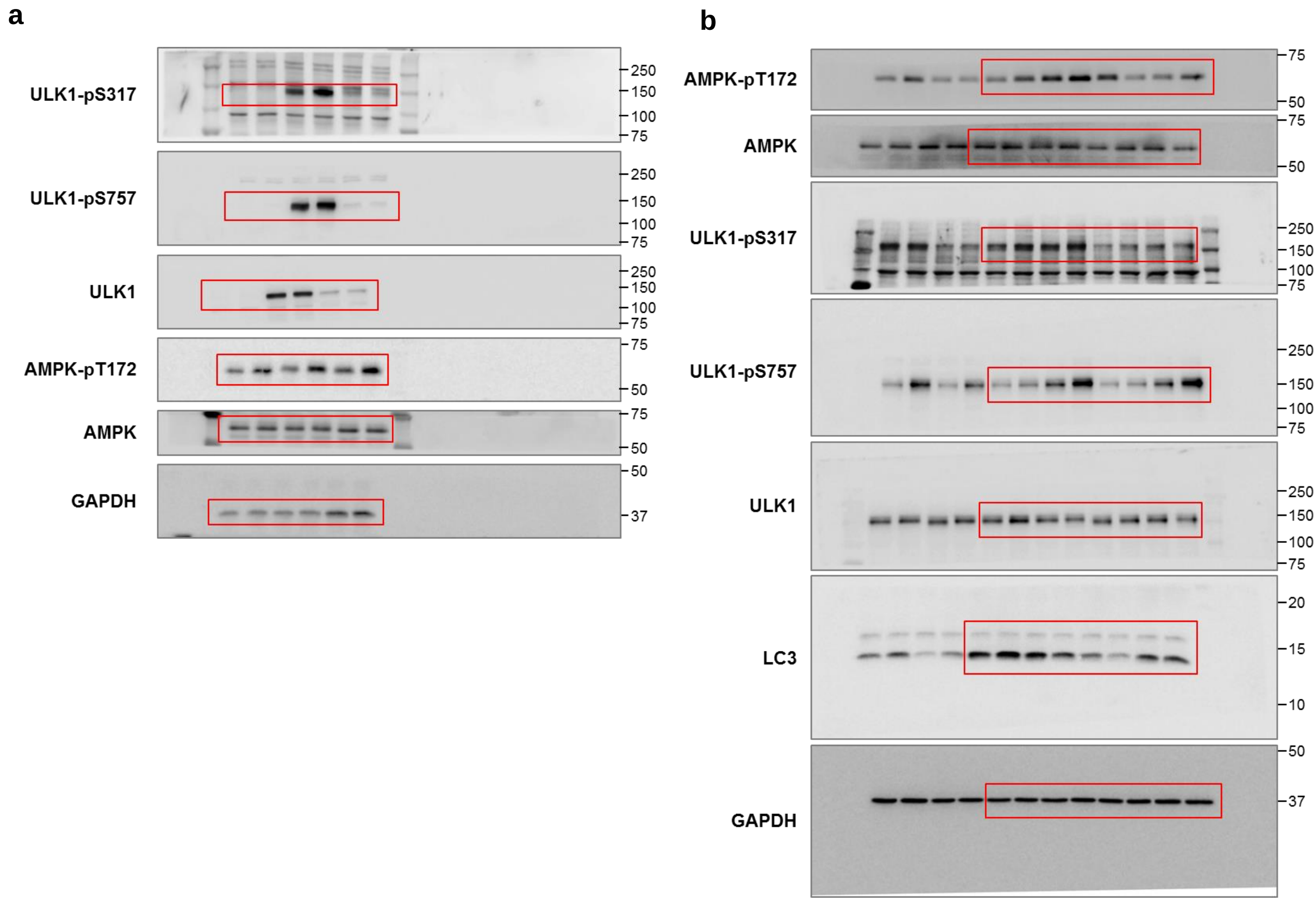

Supplementary Figure 33. Uncropped immunoblots for Figure 5h and 5j
Chemiluminiscence detections of the whole membranes for each protein detected are shown. The molecular weight is indicated in kDa on the right side. The red squares indicate the sections shown in (a) **Figure 5h**, (b) **Figure 5j**.

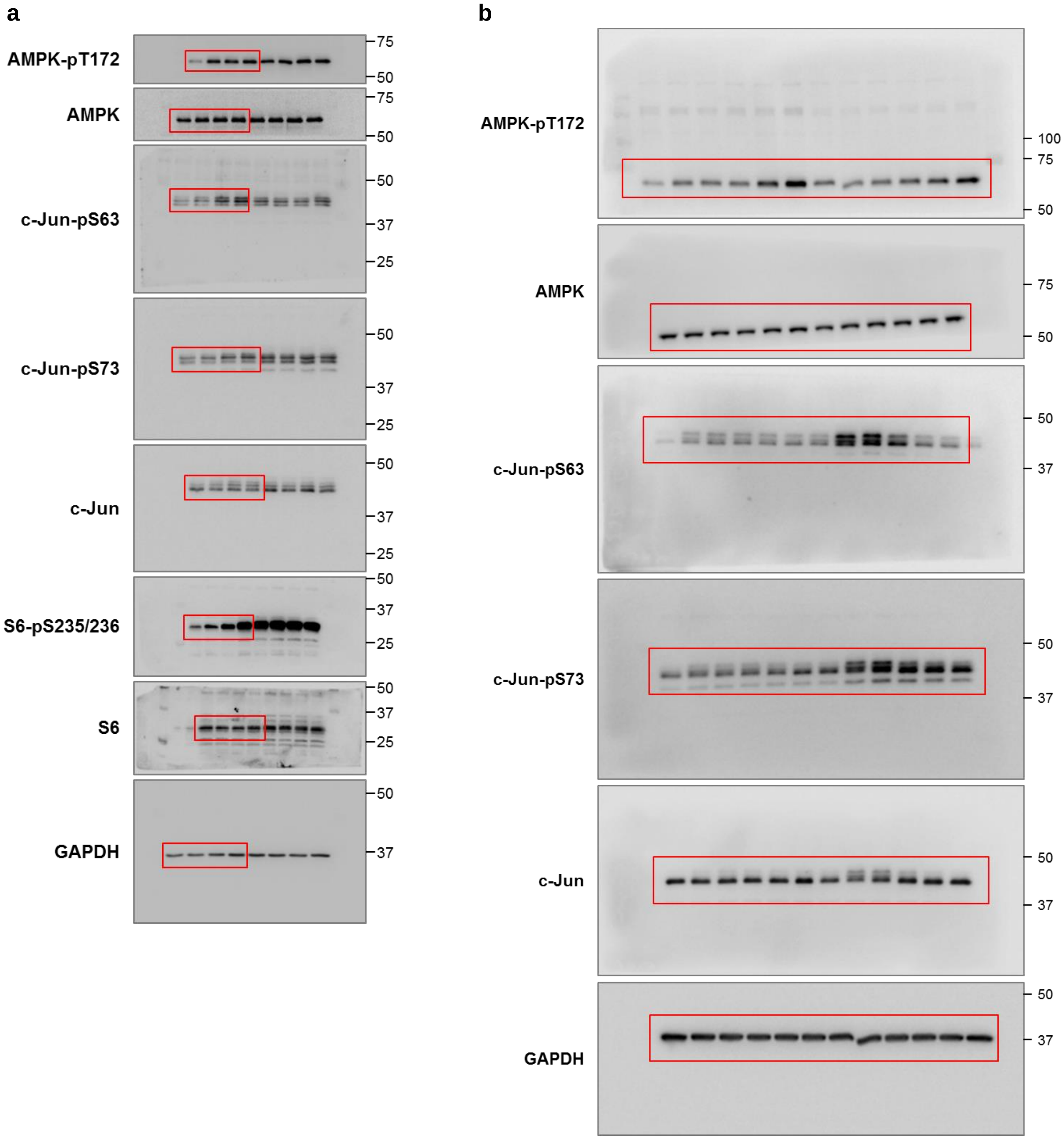

Supplementary Figure 34. Uncropped immunoblots for Figure 6d and 6e
Chemiluminiscence detections of the whole membranes for each protein detected are shown. The molecular weight is indicated in kDa on the right side. The red squares indicate the sections shown in (a) **Figure 6d**, (b) **Figure 6e**.

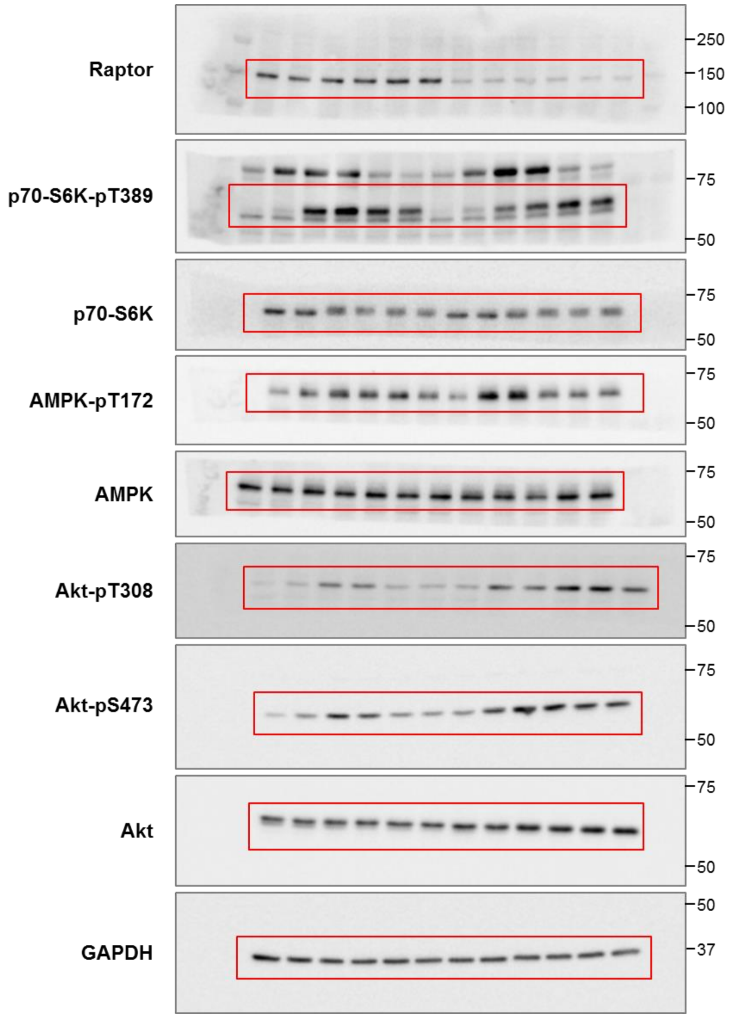

Supplementary Figure 35. Uncropped immunoblots for Figure 7b
Chemiluminiscence detections of the whole membranes for each protein detected are shown. The molecular weight is indicated in kDa on the right side. The red squares indicate the sections shown in **Figure 7b.**

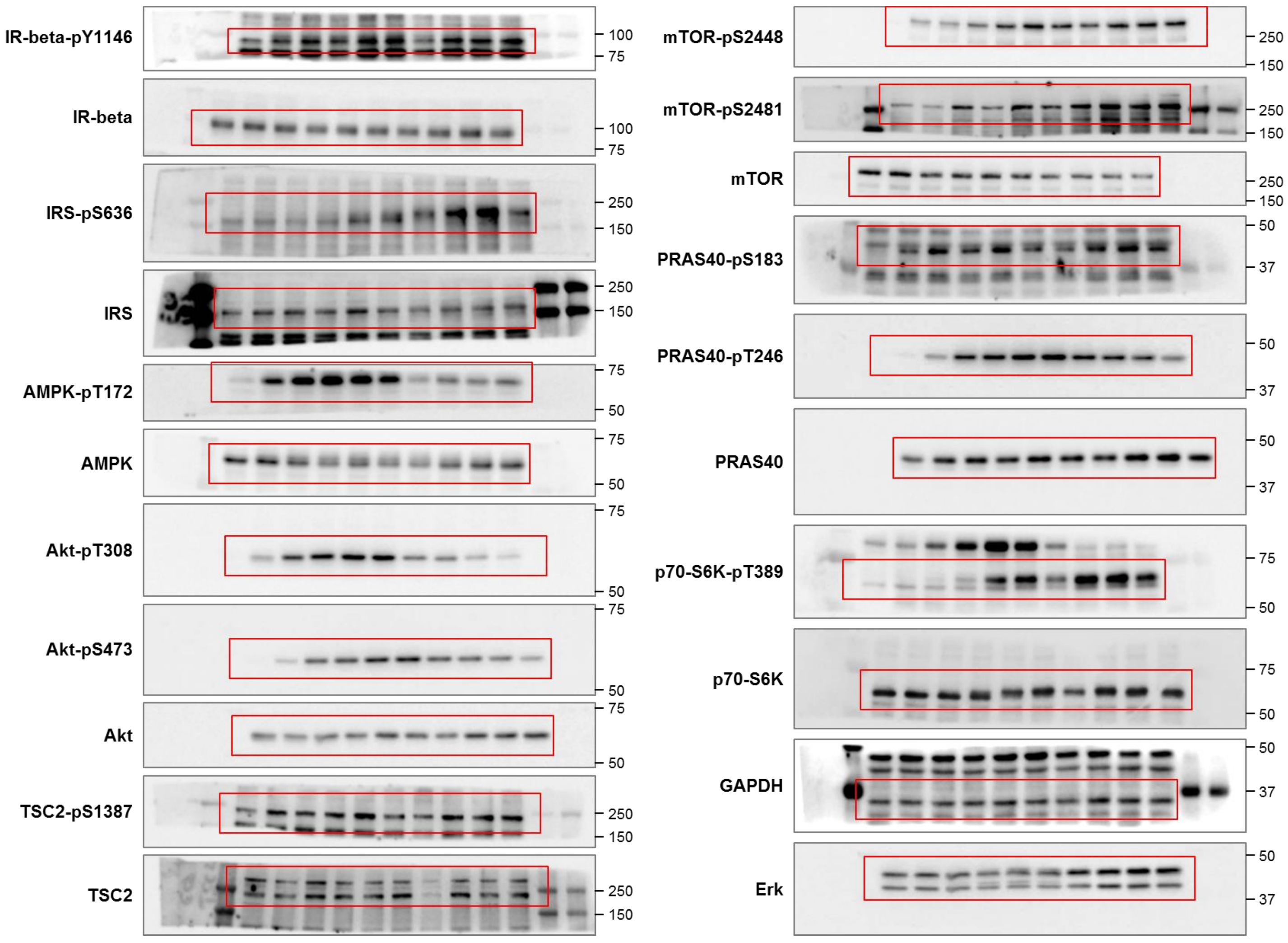

Supplementary Figure 36. Uncropped immunoblots for Supplementary Figure 2a
Chemiluminiscence detections of the whole membranes for each protein detected are shown. The molecular weight is indicated in kDa on the right side. The red squares indicate the sections shown in **Supplementary Fig. 2a.**

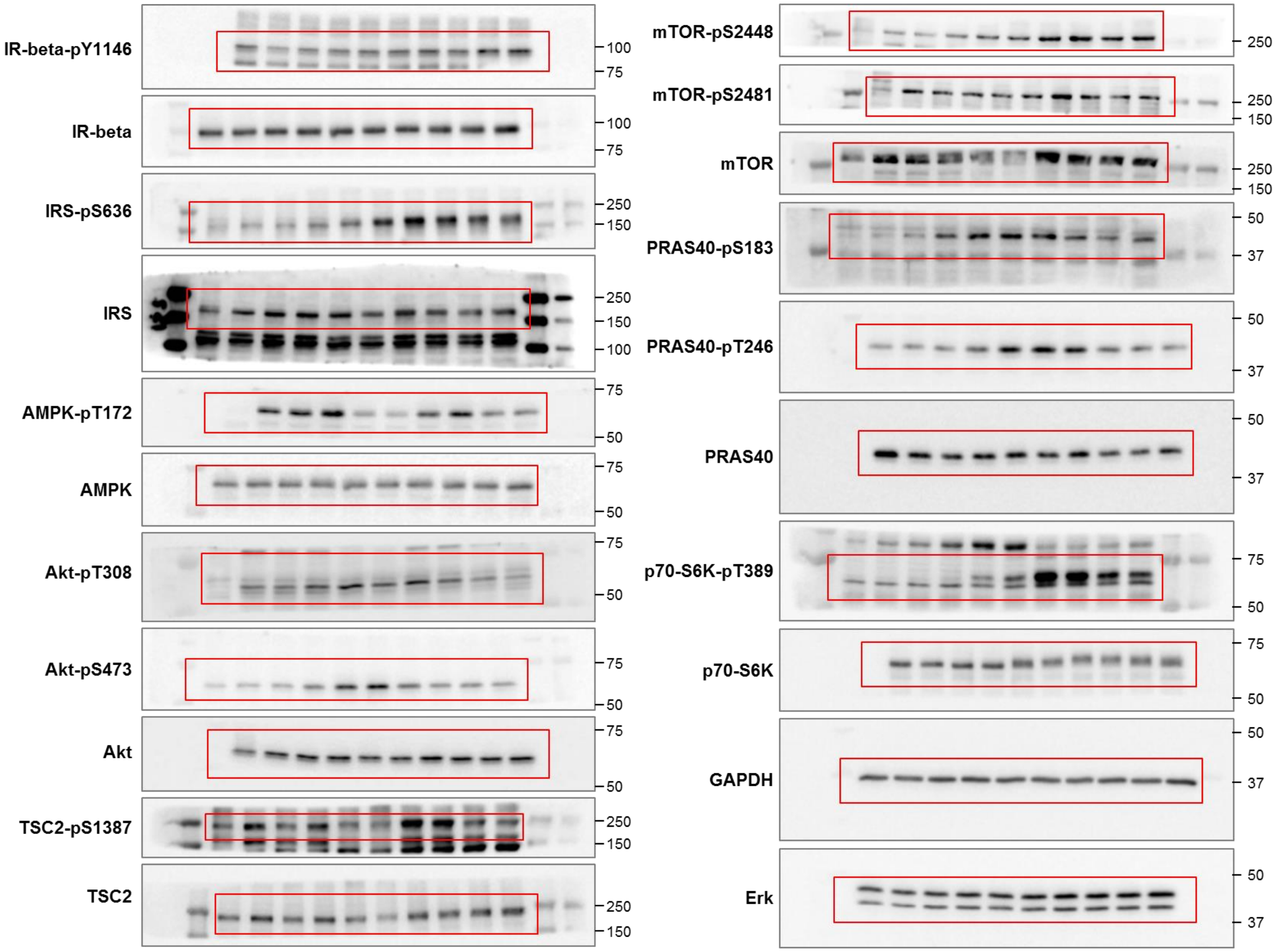

Supplementary Figure 37. Uncropped immunoblots for Supplementary Figure 2b
Chemiluminiscence detections of the whole membranes for each protein detected are shown. The molecular weight is indicated in kDa on the right side. The red squares indicate the sections shown in **Supplementary Fig. 2b.**

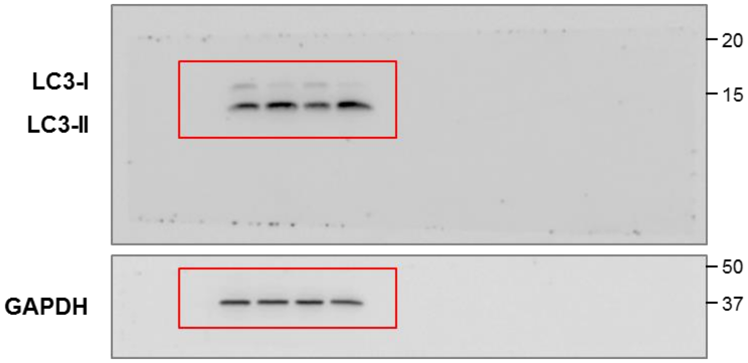

Supplementary Figure 38. Uncropped immunoblots for Supplementary Figure 22
Chemiluminiscence detections of the whole membranes for each protein detected are shown. The molecular weight is indicated in kDa on the right side. The red squares indicate the sections shown in **Supplementary Fig. 22.**

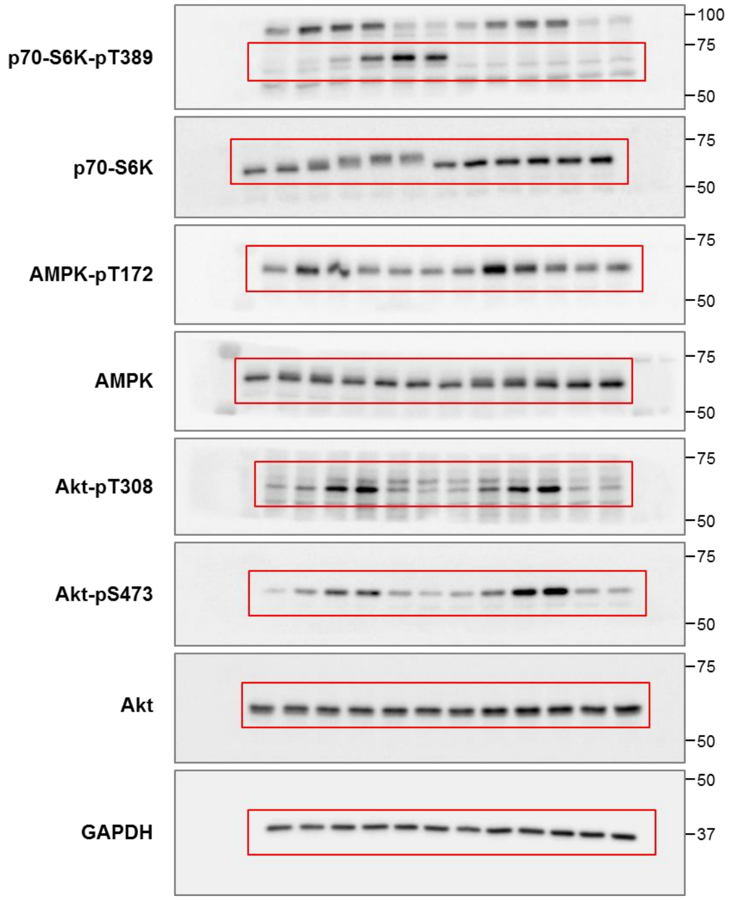

Supplementary Figure 39. Uncropped immunoblots for Supplementary Figure 27a
Chemiluminiscence detections of the whole membranes for each protein detected are shown. The molecular weight is indicated in kDa on the right side. The red squares indicate the sections shown in **Supplementary Fig. 27a.**

Parameters	
Akt_S473_phos_by_mTORC2_pS2481_first	K_1
Akt_S473_phos_by_mTORC2_pS2481_second	K_2
Akt_T308_phos_by_PI3K_p_PDK1_first	K_3
Akt_T308_phos_by_PI3K_p_PDK1_second	K_4
Akt_pT308_dephos_first	K_5
Akt_pT308_dephos_second	K_6
Akt_pS473_dephos_first	K_7
Akt_pS473_dephos_second	K_8
AMPK_T172_phos	K_9
AMPK_pT172_dephos	K_{10}
IR_beta_phos_by_Insulin:	K_{11}
IR_beta_pY1146_dephos	K_{12}
IR_beta_ready	K_{13}
IRS_phos_by_IR_beta_pY1146	K_{14}
IRS_p_phos_by_p70_S6K_pT389	K_{15}
IRS_phos_by_p70_S6K_pT389	K_{16}
IRS_pS636_turnover	K_{17}
mTORC1_S2448_activation_by_Amino_Acids	K_{18}
mTORC1_pS2448_dephos_by_TSC1_TSC2	K_{19}
mTORC2_S2481_phos_by_PI3K_variant_p	K_{20}
mTORC2_pS2481_dephos	K_{21}
p70_S6K_T389_phos_by_mTORC1_pS2448	K_{22}
p70_S6K_pT389_dephos	K_{23}
PI3K_PDK1_phos_by_IRS_p	K_{24}
PI3K_p_PDK1_dephos	K_{25}
PI3K_variant_phos_by_IR_beta_pY1146	K_{26}
PI3K_variant_p_dephos	K_{27}
PRAS40_S183_phos_by_mTORC1_pS2448_first	K_{28}
PRAS40_S183_phos_by_mTORC1_pS2448_second	K_{29}
PRAS40_T246_phos_by_Akt_pT308_first	K_{30}
PRAS40_T246_phos_by_Akt_pT308_second	K_{31}
PRAS40_pS183_dephos_first	K_{32}
PRAS40_pS183_dephos_second	K_{33}
PRAS40_pT246_dephos_first	K_{34}
PRAS40_pT246_dephos_second	K_{35}
TSC1_TSC2_S1387_phos_by_AMPK_pT172	K_{36}
TSC1_TSC2_T1462_phos_by_Akt_pT308	K_{37}
TSC1_TSC2_pS1387_dephos	K_{38}
TSC1_TSC2_pT1462_dephos	K_{39}

Parameters (AA Input)	
Akt_S473_phos_by_Amino_Acids	K_{40}
Akt_T308_phos_by_Amino_Acids	K_{41}
AMPK_T172_phos_by_Amino_Acids	K_{42}
IRS_phos_by_Amino_Acids	K_{43}
mTORC2_S2481_phos_by_Amino_Acids	K_{44}
p70_S6K_T389_phos_by_Amino_Acids	K_{45}
PI3K_PDK1_phos_by_Amino_Acids	K_{46}
PI3K_variant_phos_by_Amino_Acids	K_{47}
PRAS40_S183_phos_by_Amino_Acids	K_{48}
PRAS40_T246_phos_by_Amino_Acids	K_{49}
TSC1_TSC2_S1387_phos_by_Amino_Acids	K_{50}
TSC1_TSC2_T1462_phos_by_Amino_Acids	K_{51}

Scaling Factors		Value (fixed)
scale_Akt_pS473_obs	S_1	1
scale_Akt_pT308_obs	S_2	1
scale_AMPK_pT172_obs	S_3	1
scale_IR_beta_pY1146_obs	S_4	1
scale_IRS_pS636_obs	S_5	1
scale_mTOR_pS2448_obs	S_6	1
scale_mTOR_pS2481_obs	S_7	1
scale_p70_S6K_pT389_obs	S_8	1
scale_PRAS40_pS183_obs	S_9	1
scale_PRAS40_pT246_obs	S_{10}	1
scale_TSC1_TSC2_pS1387_obs	S_{11}	1

Observables		Value
Akt_pS473_obs	Y_1	$S_1 \cdot (X_3+X_4)$
Akt_pT308_obs	Y_2	$S_2 \cdot (X_2+X_4)$
AMPK_pT172_obs	Y_3	$S_3 \cdot X_7$
IR_beta_pY1146_obs	Y_4	$S_4 \cdot X_{10}$
IRS_pS636_obs	Y_5	$S_5 \cdot X_{14}$
mTOR_pS2448_obs	Y_6	$S_6 \cdot X_{16}$
mTOR_pS2481_obs	Y_7	$S_7 \cdot X_{18}$
p70_S6K_pT389_obs	Y_8	$S_8 \cdot X_{20}$
PRAS40_pS183_obs	Y_9	$S_9 \cdot (X_{26}+X_{28})$
PRAS40_pT246_obs	Y_{10}	$S_{10} \cdot (X_{27}+X_{28})$
TSC1_TSC2_pS1387_obs	Y_{11}	$S_{11} \cdot X_{30}$

Constraints	Value	λ
CS_1	$K_1 = K_2$	10
CS_2	$K_3 = K_4$	10
CS_3	$K_5 = K_6$	5
CS_4	$K_7 = K_8$	5
CS_5	$K_{15} = K_{16}$	5
CS_6	$K_{28} = K_{29}$	10
CS_7	$K_{30} = K_{31}$	10
CS_8	$K_{32} = K_{33}$	5
CS_9	$K_{34} = K_{35}$	5

Supplementary Table 1. Legend of the model variables

This table provides the unique codes for the model kinetic rate constants (Ki), scaling factors (Si), observables (Yi) and constraints (Csi). The block 'Parameters (AA Input)' shows the kinetic rate constants of the additional reactions used in the combinatorial expansion for determining the levels of additional aa inputs (see **Table 1**). The lambda term in the block 'Constraints' defines the constraint strength (higher values means harder constraints) as implemented in Potterswheel.

Variables	Initial Value	Ordinary Differential Equations
Akt	300	$\dot{X}_1 = + K_5 \cdot X_2 - K_1 \cdot X_1 \cdot X_{18} + K_7 \cdot X_3 - K_3 \cdot X_1 \cdot X_{22}$
Akt_pT308	0	$\dot{X}_2 = + K_3 \cdot X_1 \cdot X_{22} - K_2 \cdot X_2 \cdot X_{18} + K_8 \cdot X_4 - K_5 \cdot X_2$
Akt_pS473	0	$\dot{X}_3 = + K_1 \cdot X_1 \cdot X_{18} - K_4 \cdot X_3 \cdot X_{22} + K_6 \cdot X_4 - K_7 \cdot X_3$
Akt_pT308_pS473	0	$\dot{X}_4 = + K_2 \cdot X_2 \cdot X_{18} - K_6 \cdot X_4 + K_4 \cdot X_3 \cdot X_{22} - K_8 \cdot X_4$
Amino Acids (Input)	1	$\dot{X}_5 = const_{AA}$
AMPK	50	$\dot{X}_6 = + K_{10} \cdot X_7 - K_9 \cdot X_6 \cdot X_{13}$
AMPK_pT172	0	$\dot{X}_7 = + K_9 \cdot X_6 \cdot X_{13} - K_{10} \cdot X_7$
Insulin (Input)	1	$\dot{X}_8 = const_{Ins}$
IR_beta	50	$\dot{X}_9 = + K_{13} \cdot X_{11} - K_{11} \cdot X_9 \cdot X_8$
IR_beta_pY1146	0	$\dot{X}_{10} = + K_{11} \cdot X_9 \cdot X_8 - K_{12} \cdot X_{10}$
IR_beta_refractory	0	$\dot{X}_{11} = + K_{12} \cdot X_{10} - K_{13} \cdot X_{11}$
IRS	150	$\dot{X}_{12} = + K_{17} \cdot X_{14} - K_{14} \cdot X_{12} \cdot X_{10} - K_{16} \cdot X_{12} \cdot X_{20}$
IRS_p	0	$\dot{X}_{13} = + K_{14} \cdot X_{12} \cdot X_{10} - K_{15} \cdot X_{13} \cdot X_{20}$
IRS_pS636	0	$\dot{X}_{14} = + K_{15} \cdot X_{13} \cdot X_{20} - K_{17} \cdot X_{14} + K_{16} \cdot X_{12} \cdot X_{20}$
mTORC1	100	$\dot{X}_{15} = + K_{19} \cdot X_{16} \cdot (X_{29} + X_{30}) - K_{18} \cdot X_{15} \cdot X_5$
mTORC1_pS2448	0	$\dot{X}_{16} = + K_{18} \cdot X_{15} \cdot X_5 - K_{19} \cdot X_{16} \cdot (X_{29} + X_{30})$
mTORC2	100	$\dot{X}_{17} = + K_{21} \cdot X_{18} - K_{20} \cdot X_{17} \cdot X_{24}$
mTORC2_pS2481	0	$\dot{X}_{18} = + K_{20} \cdot X_{17} \cdot X_{24} - K_{21} \cdot X_{18}$
p70_S6K	100	$\dot{X}_{19} = + K_{23} \cdot X_{20} - K_{22} \cdot X_{19} \cdot X_{16}$
p70_S6K_pT389	0	$\dot{X}_{20} = + K_{22} \cdot X_{19} \cdot X_{16} - K_{23} \cdot X_{20}$
PI3K_PDK1	50	$\dot{X}_{21} = + K_{25} \cdot X_{22} - K_{24} \cdot X_{21} \cdot X_{13}$
PI3K_p_PDK1	0	$\dot{X}_{22} = + K_{24} \cdot X_{21} \cdot X_{13} - K_{25} \cdot X_{22}$
PI3K_Variant	50	$\dot{X}_{23} = + K_{27} \cdot X_{24} - K_{26} \cdot X_{23} \cdot X_{10}$
PI3K_Variant_p	0	$\dot{X}_{24} = + K_{26} \cdot X_{23} \cdot X_{10} - K_{27} \cdot X_{24}$
PRAS40	20	$\dot{X}_{25} = + K_{32} \cdot X_{26} - K_{28} \cdot X_{25} \cdot X_{16} + K_{34} \cdot X_{27} - K_{30} \cdot X_{25} \cdot (X_2 + X_4)$
PRAS40_pS183	0	$\dot{X}_{26} = + K_{28} \cdot X_{25} \cdot X_{16} - K_{31} \cdot X_{26} \cdot (X_2 + X_4) + K_{35} \cdot X_{28} - K_{32} \cdot X_{26}$
PRAS40_pT246	0	$\dot{X}_{27} = + K_{30} \cdot X_{25} \cdot (X_2 + X_4) - K_{29} \cdot X_{27} \cdot X_{16} + K_{33} \cdot X_{28} - K_{34} \cdot X_{27}$
PRAS40_pT246_pS183	0	$\dot{X}_{28} = + K_{29} \cdot X_{27} \cdot X_{16} - X_{33} \cdot X_{28} + K_{31} \cdot X_{26} \cdot (X_2 + X_4) - K_{35} \cdot X_{28}$
TSC1_TSC2	50	$\dot{X}_{29} = + K_{38} \cdot X_{30} - K_{36} \cdot X_{29} \cdot X_7 + K_{39} \cdot X_{31} - K_{37} \cdot X_{29} \cdot (X_2 + X_4)$
TSC1_TSC2_pS1387	0	$\dot{X}_{30} = + K_{36} \cdot X_{29} \cdot X_7 - K_{38} \cdot X_{30}$
TSC1_TSC2_pT1462	0	$\dot{X}_{31} = + K_{37} \cdot X_{29} \cdot (X_2 + X_4) - K_{39} \cdot X_{31}$

Supplementary Table 2. ODEs table of the model assuming a single amino acid input directly on mTORC1

The Ordinary Differential Equations (ODEs) and the initial values of the species are provided in this table. This information is shared among all the model variants presented in **Table 1** as well as for the final model.

Replacement ODEs for AA Input Parameters (Hypothesis Expansions)

AA => AKT-pS473	$\dot{X}_1 = + K_5 \cdot X_2 - K_1 \cdot X_1 \cdot X_{18} + K_7 \cdot X_3 - K_3 \cdot X_1 \cdot X_{22} - K_{40} \cdot X_1 \cdot X_5$
	$\dot{X}_2 = + K_3 \cdot X_1 \cdot X_{22} - K_2 \cdot X_2 \cdot X_{18} + K_8 \cdot X_4 - K_5 \cdot X_2 - K_{40} \cdot X_2 \cdot X_5$
	$\dot{X}_3 = + K_1 \cdot X_1 \cdot X_{18} - K_4 \cdot X_3 \cdot X_{22} + K_6 \cdot X_4 - K_7 \cdot X_3 + K_{40} \cdot X_1 \cdot X_5$
	$\dot{X}_4 = + K_2 \cdot X_2 \cdot X_{18} - K_6 \cdot X_4 + K_4 \cdot X_3 \cdot X_{22} - K_8 \cdot X_4 + K_{40} \cdot X_2 \cdot X_5$
AA => AKT-pT308	$\dot{X}_1 = + K_5 \cdot X_2 - K_1 \cdot X_1 \cdot X_{18} + K_7 \cdot X_3 - K_3 \cdot X_1 \cdot X_{22} - K_{41} \cdot X_1 \cdot X_5$
	$\dot{X}_2 = + K_3 \cdot X_1 \cdot X_{22} - K_2 \cdot X_2 \cdot X_{18} + K_8 \cdot X_4 - K_5 \cdot X_2 + K_{41} \cdot X_1 \cdot X_5$
	$\dot{X}_3 = + K_1 \cdot X_1 \cdot X_{18} - K_4 \cdot X_3 \cdot X_{22} + K_6 \cdot X_4 - K_7 \cdot X_3 - K_{41} \cdot X_3 \cdot X_5$
	$\dot{X}_4 = + K_2 \cdot X_2 \cdot X_{18} - K_6 \cdot X_4 + K_4 \cdot X_3 \cdot X_{22} - K_8 \cdot X_4 + K_{41} \cdot X_3 \cdot X_5$
AA => AKT-pS473	$\dot{X}_1 = + K_5 \cdot X_2 - K_1 \cdot X_1 \cdot X_{18} + K_7 \cdot X_3 - K_3 \cdot X_1 \cdot X_{22} - K_{40} \cdot X_1 \cdot X_5 - K_{41} \cdot X_1 \cdot X_5$
AA => AKT-pT308	$\dot{X}_2 = + K_3 \cdot X_1 \cdot X_{22} - K_2 \cdot X_2 \cdot X_{18} + K_8 \cdot X_4 - K_5 \cdot X_2 + K_{41} \cdot X_1 \cdot X_5 - K_{40} \cdot X_2 \cdot X_5$
	$\dot{X}_3 = + K_1 \cdot X_1 \cdot X_{18} - K_4 \cdot X_3 \cdot X_{22} + K_6 \cdot X_4 - K_7 \cdot X_3 + K_{40} \cdot X_1 \cdot X_5 - K_{41} \cdot X_3 \cdot X_5$
	$\dot{X}_4 = + K_2 \cdot X_2 \cdot X_{18} - K_6 \cdot X_4 + K_4 \cdot X_3 \cdot X_{22} - K_8 \cdot X_4 + K_{40} \cdot X_2 \cdot X_5 + K_{41} \cdot X_3 \cdot X_5$
AA => AMPK-pT172	$\dot{X}_6 = + K_{10} \cdot X_7 - K_9 \cdot X_6 \cdot X_{13} - K_{42} \cdot X_6 \cdot X_5$
	$\dot{X}_7 = + K_9 \cdot X_6 \cdot X_{13} - K_{10} \cdot X_7 + K_{42} \cdot X_6 \cdot X_5$
AA => IRS-p	$\dot{X}_{12} = + K_{17} \cdot X_{14} - K_{14} \cdot X_{12} \cdot X_{10} - K_{16} \cdot X_{12} \cdot X_{20} - K_{43} \cdot X_{12} \cdot X_5$
	$\dot{X}_{13} = + K_{14} \cdot X_{12} \cdot X_{10} - K_{15} \cdot X_{13} \cdot X_{20} + K_{43} \cdot X_{12} \cdot X_5$
AA => mTORC2-pS2481	$\dot{X}_{17} = + K_{21} \cdot X_{18} - K_{20} \cdot X_{17} \cdot X_{24} - K_{44} \cdot X_{17} \cdot X_5$
	$\dot{X}_{18} = + K_{20} \cdot X_{17} \cdot X_{24} - K_{21} \cdot X_{18} + K_{44} \cdot X_{17} \cdot X_5$
AA => p70_S6K-pT389	$\dot{X}_{19} = + K_{23} \cdot X_{20} - K_{22} \cdot X_{19} \cdot X_{16} - K_{45} \cdot X_{19} \cdot X_5$
	$\dot{X}_{20} = + K_{22} \cdot X_{19} \cdot X_{16} - K_{23} \cdot X_{20} + K_{45} \cdot X_{19} \cdot X_5$
AA => PI3K_PDK1-p	$\dot{X}_{21} = + K_{25} \cdot X_{22} - K_{24} \cdot X_{21} \cdot X_{13} - K_{46} \cdot X_{21} \cdot X_5$
	$\dot{X}_{22} = + K_{24} \cdot X_{21} \cdot X_{13} - K_{25} \cdot X_{22} + K_{46} \cdot X_{21} \cdot X_5$
AA => PI3K_Variant-p	$\dot{X}_{23} = + K_{27} \cdot X_{24} - K_{26} \cdot X_{23} \cdot X_{10} - K_{47} \cdot X_{23} \cdot X_5$
	$\dot{X}_{24} = + K_{26} \cdot X_{23} \cdot X_{10} - K_{27} \cdot X_{24} + K_{47} \cdot X_{23} \cdot X_5$
AA => PRAS40-pS183	$\dot{X}_{25} = + K_{32} \cdot X_{26} - K_{28} \cdot X_{25} \cdot X_{16} + K_{34} \cdot X_{27} - K_{30} \cdot X_{25} \cdot (X_2+X_4) - K_{48} \cdot X_{25} \cdot X_5$
	$\dot{X}_{26} = + K_{28} \cdot X_{25} \cdot X_{16} - K_{31} \cdot X_{26} \cdot (X_2+X_4) + K_{35} \cdot X_{28} - K_{32} \cdot X_{26} + K_{48} \cdot X_{25} \cdot X_5$
	$\dot{X}_{27} = + K_{30} \cdot X_{25} \cdot (X_2+X_4) - K_{29} \cdot X_{27} \cdot X_{16} + K_{33} \cdot X_{28} - K_{34} \cdot X_{27} - K_{48} \cdot X_{27} \cdot X_5$
	$\dot{X}_{28} = + K_{29} \cdot X_{27} \cdot X_{16} - K_{33} \cdot X_{28} + K_{31} \cdot X_{26} \cdot (X_2+X_4) - K_{35} \cdot X_{28} + K_{48} \cdot X_{27} \cdot X_5$
AA => PRAS40-pT246	$\dot{X}_{25} = + K_{32} \cdot X_{26} - K_{28} \cdot X_{25} \cdot X_{16} + K_{34} \cdot X_{27} - K_{30} \cdot X_{25} \cdot (X_2+X_4) - K_{49} \cdot X_{25} \cdot X_5$
	$\dot{X}_{26} = + K_{28} \cdot X_{25} \cdot X_{16} - K_{31} \cdot X_{26} \cdot (X_2+X_4) + K_{35} \cdot X_{28} - K_{32} \cdot X_{26} - K_{49} \cdot X_{26} \cdot X_5$
	$\dot{X}_{27} = + K_{30} \cdot X_{25} \cdot (X_2+X_4) - K_{29} \cdot X_{27} \cdot X_{16} + K_{33} \cdot X_{28} - K_{34} \cdot X_{27} + K_{49} \cdot X_{25} \cdot X_5$
	$\dot{X}_{28} = + K_{29} \cdot X_{27} \cdot X_{16} - K_{33} \cdot X_{28} + K_{31} \cdot X_{26} \cdot (X_2+X_4) - K_{35} \cdot X_{28} + K_{49} \cdot X_{26} \cdot X_5$
AA => TSC1_TSC2-pS1387	$\dot{X}_{29} = + K_{38} \cdot X_{30} - K_{36} \cdot X_{29} \cdot X_7 + K_{39} \cdot X_{31} - K_{37} \cdot X_{29} \cdot (X_2+X_4) - K_{50} \cdot X_{29} \cdot X_5$
	$\dot{X}_{30} = + K_{36} \cdot X_{29} \cdot X_7 - K_{38} \cdot X_{30} + K_{50} \cdot X_{29} \cdot X_5$
AA => TSC1_TSC2-pT1462	$\dot{X}_{29} = + K_{38} \cdot X_{30} - K_{36} \cdot X_{29} \cdot X_7 + K_{39} \cdot X_{31} - K_{37} \cdot X_{29} \cdot (X_2+X_4) - K_{51} \cdot X_{29} \cdot X_5$
	$\dot{X}_{31} = + K_{37} \cdot X_{29} \cdot (X_2+X_4) - K_{39} \cdot X_{31} + K_{51} \cdot X_{29} \cdot X_5$

Supplementary Table 3. Replaced ODEs for the additional model variants

Each model variant presented in **Table 1** contains a specific set of additional aa inputs besides the canonical mTORC1 input. Each of these inputs was implemented by simply replacing the corresponding group of ODEs to the canonical model described in **Supplementary Table 2**. For instance, the model including the additional aa-input to IRS contained all the ODEs listed in **Supplementary Table 2**, but replaces the ODEs X12 and X13 with those presented in this table.

Code	Kinetic rate parameter names (min^{-1})	Rounds of parameter estimation + MOTA non-identifiability analysis										Final parameter values			PLE 68.27% point-wise CI		
		IR-beta module		*Complete model*													
		Round 1	Round 2	Round 1	Round 2	Round 3	Round 4	Round 5	Round 6	Round 7	Round 8	Value	Mean	StdDev	Lower PL	Upper PL	ID Flag
K_{1}	Akt_S473_phos_by_mTORC2_pS2481_first					Fixed						1.32E-05	1.5E-05	0.000001395 (9%)	0	0.000619	pra. nID
K_{2}	Akt_S473_phos_by_mTORC2_pS2481_second			Fixed								0.159093	0.166623	0.00471646 (3%)	0.145	0.2137	
K_{3}	Akt_T308_phos_by_PI3K_p_PDK1_first						Locked					7.47437	3.33613	2.55011 (76%)	1.3	15.39	
K_{4}	Akt_T308_phos_by_PI3K_p_PDK1_second							Fixed				7.47345	7.47247	0.00287411 (0%)	0.905	11.73	
K_{5}	Akt_pT308_dephos_first							Fixed				88.9654	88.2913	0.607374 (1%)	68.93	Inf+	pra. nID
K_{6}	Akt_pT308_dephos_second							Fixed				88.9639	88.2915	0.60802 (1%)	69.4	103.4	
K_{7}	Akt_pS473_dephos_first			Fixed								0.376999	0.4067	0.01575 (4%)	0.3513	0.4241	
K_{8}	Akt_pS473_dephos_second			Fixed								0.380005	0.40107	0.0168014 (4%)	0.2819	0.4885	
K_{9}	AMPK_T172_phos										Fixed	0.490602	0.515641	0.117259 (23%)	0.01635	0.5217	
K_{10}	AMPK_pT172_dephos									Locked		165.704	34.7613	38.4358 (111%)	9.449	9105	
K_{11}	IR_beta_phos_by_Insulin	Fixed										0.0203796	0.020375	4.31382e-06 (0%)	0.01359	0.03129	
K_{12}	IR_beta_pY1146_dephos	Fixed										0.493514	0.493221	0.00013662 (0%)	0.2917	0.7886	
K_{13}	IR_beta_ready		Locked									323.611	162.013	65.7521 (41%)	0.07377	Inf+	pra. nID
K_{14}	IRS_phos_by_IR_beta_pY1146			Fixed								2.11894	2.44782	0.241862 (10%)	1.696	2.344	
K_{15}	IRS_p_phos_by_p70_S6K_pT389			Fixed								0.311275	0.282068	0.0119665 (4%)	0.2808	0.3312	
K_{16}	IRS_phos_by_p70_S6K_pT389			Fixed								0.0238067	0.023534	0.000412484 (2%)	0.02251	0.02659	
K_{17}	IRS_pS636_turnover			Assumed								25	-	-	-	-	-
K_{18}	mTORC1_S2448_activation_by_Amino_Acids			Fixed								0.0156992	0.015978	0.00163941 (10%)	0.01413	0.0166	
K_{19}	mTORC1_pS2448_dephos_by_TSC1_TSC2				Fixed							0.0086977	0.008558	7.41006e-05 (1%)	0.006942	0.01052	
K_{20}	mTORC2_S2481_phos_by_PI3K_variant_p										Fixed	0.120736	0.120637	0.0148594 (12%)	0.1067	0.2757	
K_{21}	mTORC2_pS2481_dephos									Locked		1.42511	12.3457	33.3652 (270%)	1.352	1.487	
K_{22}	p70_S6K_T389_phos_by_mTORC1_pS2448			Fixed								0.005379	0.005383	0.000159511 (3%)	0.005199	0.005852	
K_{23}	p70_S6K_pT389_dephos			Fixed								0.0884553	0.090215	0.00474616 (5%)	0.0841	0.0934	
K_{24}	PI3K_PDK1_phos_by_IRS_p			Assumed								0.0003	-	-	-	-	-
K_{25}	PI3K_p_PDK1_dephos			Assumed								0.1	-	-	-	-	-
K_{26}	PI3K_variant_phos_by_IR_beta_pY1146			Assumed								0.0003	-	-	-	-	-
K_{27}	PI3K_variant_p_dephos			Assumed								0.07	-	-	-	-	-
K_{28}	PRAS40_S183_phos_by_mTORC1_pS2448_first				Fixed							0.15881	0.163347	0.0182618 (11%)	0.1318	0.1726	
K_{29}	PRAS40_S183_phos_by_mTORC1_pS2448_second					Fixed						0.0683009	0.064447	0.00595447 (9%)	0.04603	0.07975	
K_{30}	PRAS40_T246_phos_by_Akt_pT308_first							Locked				0.279344	0.171288	0.159113 (93%)	0.007077	Inf+	pra. nID
K_{31}	PRAS40_T246_phos_by_Akt_pT308_second								Fixed			0.279401	0.280084	0.0024506 (1%)	0	7.04	pra. nID
K_{32}	PRAS40_pS183_dephos_first				Fixed							1.8706	1.93544	0.279498 (14%)	1.256	3.83	
K_{33}	PRAS40_pS183_dephos_second					Fixed						1.88453	1.88415	0.00642049 (0%)	1.809	3.42	
K_{34}	PRAS40_pT246_dephos_first								Fixed			11.8759	11.8834	0.0448292 (0%)	0.9042	23.21	
K_{35}	PRAS40_pT246_dephos_second								Fixed			11.876	11.8839	0.0473507 (0%)	2.798	25.98	
K_{36}	TSC1_TSC2_S1387_phos_by_AMPK_pT172									Fixed		0.0017577	0.00176	0.000127567 (7%)	0.001355	0.00241	
K_{37}	TSC1_TSC2_T1462_phos_by_Akt_pT308									Fixed		1.52417	1.60111	0.223493 (14%)	0.03067	1.681	
K_{38}	TSC1_TSC2_pS1387_dephos								Locked			0.25319	0.285245	0.0367119 (13%)	0.1927	0.2692	
K_{39}	TSC1_TSC2_pT1462_dephos								Locked			147.239	123.742	96.6807 (78%)	135.9	Inf+	pra. nID
K_{42}	AMPK_T172_phos_by_Amino_Acids										Fixed	17.6284	18.5468	2.47774 (13%)	12.42	447.7	
K_{43}	IRS_phos_by_Amino_Acids			Fixed								0.0331672	0.035685	0.00135759 (4%)	0.03178	0.04467	
K_{44}	mTORC2_S2481_phos_by_Amino_Acids										Fixed	0.0268658	0.028132	0.00222685 (8%)	0.02558	0.04363	

Supplementary Table 4. Parameter table for the final model including the quadruple amino acid inputs

Multiple rounds of alternate parameter estimation and MOTA identifiability analysis were performed in order to determine the model kinetic rate constant parameters. MOTA correlation plots are provided in **Supplementary Figures 4-12**. The insulin receptor (IR-beta) module (codes: K11, K12 and K13) was estimated separately a priori in two rounds, whereas the remaining parameters were successively estimated within 8 rounds. The internal round columns include the following labels: Assumed, Fixed and Locked. A parameter was said to be assumed when it was assumed a priori, and therefore was not estimated. The only assumed parameters were related to the PI3K dynamics, as data was lacking. A parameter was said fixed when it could be estimated and identified based on MOTA analysis within a confidence of variance lower than 15% or a correlation coefficient lower than 0.9. A parameter was said locked when it was fixed without being completely identifiable according to MOTA analysis. This was done only when it belonged to a tuple of related parameters where each parameter in this tuple only related to the same parameter tuple. Since this correlation was local and completely confined to the tuple parameters, it did not affect the other unrelated parameters. The parameters in the tuple were only linearly affected. For each parameter, the final value, mean, standard deviation and coefficient of variance are reported as computed from the best 50% of 2500 fits (see Materials and Methods for more details). PLE identifiability analysis was also computed reporting practical non-identifiability for 6 parameters. These either involved species of secondary importance to our study (e.g PRAS40) or phosphatases for which we did not have data. Interestingly, PLE analysis sometimes revealed close confidence intervals, whereas the corresponding statistical deviation computed from the group of best fit showed significantly high variances (e.g. mTORC2-p2481-dephos). Conversely, PLE analyses reported two parameters as practically non-identifiable (PRAS40-pT246 first and second phosphorylation), respectively on the left and on the right confidence interval, whereas these two parameters were constrained together, and therefore the intersected PLE should have been 0.008 and 7.04 for both. Hence, we conclude that using both the analyses in combination increased the confidence of our results.

New/Replacement Parameters, Scaling Factors, Observables and Constraints (p70_S6K_pT229 activation by PI3K_p_PDK1)

Parameters	
IRS_p_phos_by_p70_S6K_pT229_pT389	K_{52}
IRS_phos_by_p70_S6K_pT229_pT389	K_{53}
p70_S6K_T229_phos_by_PI3K_p_PDK1_first	K_{54}
p70_S6K_T229_phos_by_PI3K_p_PDK1_second	K_{55}
p70_S6K_T389_phos_by_mTORC1_pS2448_first	K_{56}
p70_S6K_T389_phos_by_mTORC1_pS2448_second	K_{57}
p70_S6K_pT229_dephos_first	K_{58}
p70_S6K_pT229_dephos_second	K_{59}
p70_S6K_pT389_dephos_first	K_{60}
p70_S6K_pT389_dephos_second	K_{61}

Scaling Factors		Value (fixed)
scale_p70_S6K_pT229_obs	S_{12}	1

Observables		Value
p70_S6K_pT389_obs	Y_8	$S_8 \cdot (X_{20}+X_{32})$
p70_S6K_pT229_obs	Y_{12}	$S_{12} \cdot (X_{31}+X_{32})$

Constraints	Value	λ
CS_{10}	$K_{54} = K_{55}$	10
CS_{11}	$K_{55} = K_{56}$	10
CS_{12}	$K_{58} = K_{59}$	5
CS_{13}	$K_{60} = K_{61}$	5

Supplementary Table 5. Legend of the model variables for the new p70-S6K module

This table provides the unique codes for the model kinetic rate constants (Ki), scaling factors (Si), observables (Yi) and constraints (Csi), representing the new p70-S6K module. This new module was added to the model presented in **Supplementary Table 1**. The lambda term in the block 'Constraints' defines the constraint strength (higher values means harder constraints) as implemented in Potterswheel.

New/Replacement ODEs (p70_S6K_pT229 activation by PI3K_p_PDK1)

Variables	Starting Value	Ordinary Differential Equations
IRS	150	$\dot{X}_{12} = + K_{17} \cdot X_{14} - K_{14} \cdot X_{12} \cdot X_{10} - K_{53} \cdot X_{12} \cdot X_{32}$
IRS_p	0	$\dot{X}_{13} = + K_{14} \cdot X_{12} \cdot X_{10} - K_{52} \cdot X_{13} \cdot X_{32}$
IRS_pS636	0	$\dot{X}_{14} = + K_{52} \cdot X_{13} \cdot X_{20} - K_{17} \cdot X_{14} + K_{53} \cdot X_{12} \cdot X_{32}$
p70_S6K	100	$\dot{X}_{19} = + K_{58} \cdot X_{32} - K_{54} \cdot X_{19} \cdot X_{22} + K_{60} \cdot X_{20} - K_{56} \cdot X_{19} \cdot X_{16}$
p70_S6K_pT389	0	$\dot{X}_{20} = + K_{56} \cdot X_{19} \cdot X_{16} - K_{55} \cdot X_{20} \cdot X_{22} + K_{59} \cdot X_{33} - K_{60} \cdot X_{20}$
p70_S6K_pT229	0	$\dot{X}_{32} = + K_{54} \cdot X_{19} \cdot X_{22} - K_{57} \cdot X_{32} \cdot X_{16} + K_{61} \cdot X_{33} - K_{59} \cdot X_{32}$
p70_S6K_pT229_pT389	0	$\dot{X}_{33} = + K_{55} \cdot X_{20} \cdot X_{22} - K_{59} \cdot X_{33} + K_{57} \cdot X_{32} \cdot X_{16} - K_{61} \cdot X_{33}$

ODEs X32 and X33 are added to the model

Supplementary Table 6. ODEs table for the new p70-S6K module

The Ordinary Differential Equations (ODEs) and the initial values of the species for the new p70-S6K module which was added to the model presented in **Supplementary Table 1-2**.

Code	Kinetic rate parameter names (min^{-1})	Rounds of parameter estimation + MOTA non-identifiability analysis		Final parameter values			PLE 68.27% point-wise CI		
		Round 1	Round 2	Value	Mean	StdDev	Lower PL	Upper PL	ID Flag
K_{24}	PI3K_PDK1_phos_by_IRS_p	Fixed		0.0001872	0.000187907	0.00000179577 (1%)	0.0001596	0.0002221	-
K_{25}	PI3K_p_PDK1_dephos	Fixed		0.1891	0.190091	0.00291422 (2%)	0.1503	0.2469	-
K_{26}	PI3K_variant_phos_by_IR_beta_pY1146	Fixed		0.000549	0.000547628	0.00000817368 (1%)	0.0003933	0.0007829	-
K_{27}	PI3K_variant_p_dephos	Fixed		0.1081	0.107712	0.00200877 (2%)	0.0733	0.1647	-
K_{52}	IRS_p_phos_by_p70_S6K_pT229_pT389	Fixed		0.3389	0.338764	0.00220412 (1%)	0.287	0.4004	-
K_{53}	IRS_phos_by_p70_S6K_pT229_pT389	Fixed		0.08638	0.0867747	0.000333436 (<1%)	0.07567	0.09969	-
K_{54}	p70_S6K_T229_phos_by_PI3K_p_PDK1_first	Fixed		0.01335	0.0134896	0.0000881493 (1%)	0.01168	0.01527	-
K_{55}	p70_S6K_T229_phos_by_PI3K_p_PDK1_second		Fixed	0.000001	0.000052838	0.000107066 (203%)	0	0.01742	pra. nID
K_{56}	p70_S6K_T389_phos_by_mTORC1_pS2448_first	Fixed		0.002613	0.00263046	0.0000421442 (2%)	0.001751	0.003686	-
K_{57}	p70_S6K_T389_phos_by_mTORC1_pS2448_second	Fixed		0.1107	0.110894	0.00147783 (1%)	0.07935	0.1485	-
K_{58}	p70_S6K_pT229_dephos_first		Fixed	0.000001	0.000231779	0.000247762 (107%)	0	0.0115	pra. nID
K_{59}	p70_S6K_pT229_dephos_second	Fixed		0.1592	0.161798	0.00184032 (1%)	0.1236	0.1983	-
K_{60}	p70_S6K_pT389_dephos_first	Fixed		1.1	1.10813	0.017634 (2%)	0.7564	1.446	-
K_{61}	p70_S6K_pT389_dephos_second	Fixed		1.102	1.11072	0.0170689 (2%)	0.7752	1.491	-

Supplementary Table 7. Parameter table for the new p70-S6K module

Table of parameter estimation and identifiability analysis for the added (or re-estimated) parameters representing the new p70-S6K module. To successfully estimate the p70-S6K module parameters, we also re-estimated the NFL parameters (K52, K53) and the PI3K module, as these were affected by p70-S6K. The parameter estimation for PI3K was possible at this stage as all the remaining network parameters (see **Supplementary Table 4**) were not re-estimated, and this therefore strongly limited the solution space. PLE identifiability analysis identified only two parameters as practically non-identifiable, due to failure in defining a lower bound for the confidence interval. Interestingly, the estimation of these two parameters suggested that the phosphorylation of p70-S6K-pT229 occurs earlier than the phosphorylation of p70-S6K-pT389, which is in line with the experimental observation that p70-S6K-pT389 phosphorylation was reduced upon wortmannin treatment using amino acids only as input (see **Figure 1b+c**). MOTA identifiability analysis table is shown in **Supplementary Figure 14**.

Supplementary References

1 Reimand, J., Kull, M., Peterson, H., Hansen, J. & Vilo, J. g:Profiler—a web-based toolset for functional profiling of gene lists from large-scale experiments. *Nucleic Acids Research* **35**, W193-W200, doi:10.1093/nar/gkm226 (2007).

2 Reimand, J., Arak, T. & Vilo, J. g:Profiler—a web server for functional interpretation of gene lists (2011 update). *Nucleic Acids Research* **39**, W307-W315, doi:10.1093/nar/gkr378 (2011).
